# [^18^F]-Radiolabelled Nanoplatforms: A Critical Review of Their Intrinsic Characteristics, Radiolabelling Methods, and Purification Techniques

**DOI:** 10.3390/molecules29071537

**Published:** 2024-03-29

**Authors:** Maëlle Deleuziere, Éric Benoist, Isabelle Quelven, Emmanuel Gras, Catherine Amiens

**Affiliations:** 1 SPCMIB, CNRS UMR 5068, Université de Toulouse III Paul Sabatier, 118 Route de Narbonne, CEDEX 9, 31062 Toulouse, France; maelle.deleuziere@univ-tlse3.fr (M.D.); eric.benoist@univ-tlse3.fr (É.B.); 2 Toulouse NeuroImaging Center (ToNIC), INSERM/UPS UMR 1214, University Hospital of Toulouse-Purpan, CEDEX 3, 31024 Toulouse, France; quelven-bertin.i@chu-toulouse.fr; 3 LCC-CNRS, Université de Toulouse, CNRS, UPS, 31077 Toulouse, France; 4 Laboratoire Hétérochimie Fondamentale et Appliquée, UMR 5069, CNRS—Université de Toulouse, 118 Route de Narbonne, CEDEX 9, 31062 Toulouse, France; emmanuel.gras@univ-tlse3.fr

**Keywords:** nanoparticle, fluorine-18, radiofluorination, conjugation, characterization, purification, PET imaging, multimodal imaging

## Abstract

A wide range of nano-objects is found in many applications of our everyday life. Recognition of their peculiar properties and ease of functionalization has prompted their engineering into multifunctional platforms that are supposed to afford efficient tools for the development of biomedical applications. However, bridging the gap between bench to bedside cannot be expected without a good knowledge of their behaviour in vivo, which can be obtained through non-invasive imaging techniques, such as positron emission tomography (PET). Their radiolabelling with [^18^F]-fluorine, a technique already well established and widely used routinely for PET imaging, with [^18^F]-FDG for example, and in preclinical investigation using [^18^F]-radiolabelled biological macromolecules, has, therefore, been developed. In this context, this review highlights the various nano-objects studied so far, the reasons behind their radiolabelling, and main in vitro and/or in vivo results obtained thereof. Then, the methods developed to introduce the radioelement are presented. Detailed indications on the chemical steps involved are provided, and the stability of the radiolabelling is discussed. Emphasis is then made on the techniques used to purify and analyse the radiolabelled nano-objects, a point that is rarely discussed despite its technical relevance and importance for accurate imaging. The pros and cons of the different methods developed are finally discussed from which future work can develop.

## 1. Introduction

Nanoparticles can be found in a large range of morphologies (sizes, shapes, porosity, etc.), and present a large panel of physico-chemical properties (lipophilicity, surface charges, optical and magnetic properties, etc.). Their large surface over volume ratio enables the grafting of a variety of functional groups at their surface, which can further modulate the properties of the nanoparticles or endorse them with new complementary properties. They are, therefore, seen as versatile nanoplatforms that can be tailored at will depending on the application envisaged. Especially in nanomedicine, the possibility to adjust their surface state to modulate their uptake by the reticuloendothelial system and to incorporate targeting agents has prompted their investigation as imaging probes, therapeutic tools, and even for theranostic applications, among others. Approaches based on nanoparticles can also be used to modulate the biodistribution and bioavailability of molecular therapeutic agents in order to improve their therapeutic index.

However, despite a large number of in vitro and preclinical studies reporting on nanoparticles, the number of nanotherapeutics that reach clinical use remains relatively low. Several barriers exist that limit the effective preclinical evaluation and clinical translation of nanoparticles to therapy. Key barriers include an insufficient understanding of the nanoparticles’ in vivo biodistribution. This is associated with an inadequate translation from animal models to humans, as well as a high inter- and intra-patient variability. This is particularly illustrated in the Enhanced Permeability and Retention (EPR) effect in tumours [1,2]. Translational and clinical studies depend on the ability to access relevant information on nanoparticle pharmacokinetics, tissue accumulation, and therapy outcome in vivo. This critical information can be obtained owing to non-invasive imaging techniques, which are powerful tools to visualize nanoparticles in vivo, and hence bridge the gap between benchtop and bedside.

A large number of imaging techniques are available including magnetic resonance imaging (MRI), X-ray computed tomography (CT), and optical molecular imaging techniques, such as near-infrared fluorescence (NIRF) imaging or fluorescence molecular tomography (FMT), as well as nuclear imaging by positron emission tomography (PET) and single-photon emission computed tomography (SPECT). CT and MRI are both very powerful anatomical imaging techniques but exhibit poor sensitivity for visualizing exogenously administered contrast agents, requiring the administration of large amounts of exogenous agents. MRI has been employed to track nanoparticles in vivo by functionalizing them with paramagnetic (manganese, gadolinium) or superparamagnetic (iron oxide) contrast agents. However, given its poor sensitivity, rather large doses should be injected. Moreover, quantification is not straightforward, as the generated contrast not only depends on the agent’s concentration but is also affected by other factors, such as relaxivity, tissue properties, and magnetic field inhomogeneities. Optical molecular imaging techniques can provide valuable information on nanoparticles’ in vivo biodistribution. Nevertheless, emitted light’s limited tissue penetration, fluorophore bleaching and quenching, or tissue absorption hamper a proper quantification of the signal. Conversely, PET and SPECT are very sensitive, as they allow the detection of sub-pM tracer concentrations and enable accurate signal quantification [3]. Given the low amounts of imaging agent needed, the biological processes under study are, therefore, assumed to be undisturbed. Quantitative information about pharmacokinetic parameters, biodistribution profiles, or target site accumulation of nanoparticles can be obtained. So, during clinical evaluation, nuclear imaging can be used to select personalized treatment for patients. PET and SPECT, which provide functional images, are combined with anatomical imaging techniques, such as CT and lately also MRI, in hybrid imaging scanners to improve resolution and obtain truly quantitative data corrected for tissue photon attenuation.

While providing reliable information, SPECT is progressively replaced by the more accurately quantitative PET. Exact attenuation correction, precise scatter correction, and high sensitivity (combined with the possibility to perform true whole-body imaging in a reasonable time) constitute the key factors for the superiority of PET over SPECT.

Employing nuclear imaging to investigate nanoparticles in vivo behaviour requires their radiolabelling. A range of PET emitters are now routinely available with short-lived radionuclides, such as gallium-68 (67.7 min), fluorine-18 (110 min) or copper-64 (12.7 h), or long half-life ones, particularly iodine-124 (4.18 d) and zirconium-89 (78.4 h).

In addition to the radionuclide half-life, a large number of parameters must be taken into account to select a radionuclide. Among the factors of choice, cost, scalability, radiolabelling strategies, radiochemical yield and purity, and the reaction’s duration are key factors to consider.

In most targeted radiometal-based radiopharmaceuticals, the metal is bound to a pharmacophore by a bifunctional chelating agent (BFCA), which forms a stable covalent linkage between the label and the targeting ligand (vector) and ensures the stable complexation of the metal in vivo [4]. In many instances, the chelator and the vector are connected via a spacer moiety to separate the individual components of the conjugate to avoid potential interference. Chelators contain several functional groups for coordination to the radiometal of choice. In contrast, non-metal radionuclides are linked to the vector by a covalent bond, directly or via a linker [5]. The versatility and applicability of covalently bound radionuclides are enormous, especially as the chemical structure is not altered [^11^C], or only slightly [^18^F], compared with PET tracers based on a radiometal.

Fluorine exists in nature only as one stable isotope, namely [^19^F], with a 100% abundance. [^18^F], the radioactive isotope of fluorine, is easily accessible since it is produced in cyclotron from inexpensive precursors. It exhibits some peculiar physical properties such as (i) a highly pure decay by emission of a positron of relatively low energy (meaning high positron yield, small mean free path, and thus good resolution and signal over noise ratio); (ii) a half-life time of 110 min, inducing an acceptable dosimetry but also leaving enough time to perform chemistry and imaging. This imaging relies on the annihilation event between the emitted positron and one electron from the surrounding matter, releasing two γ photons of 511 keV at 180°, which are detected by the camera within a short timeframe. This allows to precisely determine the location of the annihilation event and thus the position of the [^18^F] decay. This radionuclide is easily accessible since it is produced in a cyclotron from inexpensive precursors and a large number of methods are available to bind [^18^F] to vectors. All these properties have promoted an extensive use of [^18^F] for years and make it one of the most frequently applied radionuclides in diagnosis [6].

**Figure 1:**
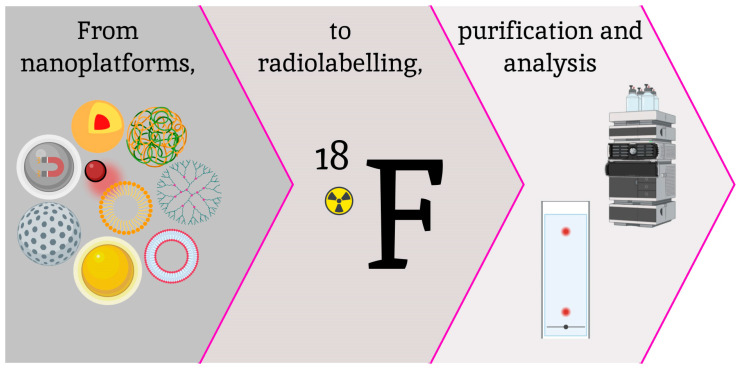
Overview of this report’s structure created with Figma.

Hence, based on a careful selection of original works found in the commonly accessed databases (SciFinder, Web of Science, PubMed, etc.), this work builds a critical review of the [^18^F]-radiolabelling of nanoplatforms. Section 2 outlines the nanoplatforms studied so far. They are either inert, to be used as carriers (such as polymers, liposomes, porous silica, etc.), or/and intrinsically active (iron-based oxides, gold, gadolinium-based oxides nanoparticles, etc.) to associate complementary diagnostics or therapeutic tools in the same object. From a chemical point of view, two main categories arise: organic nanoplatforms (block-copolymers, dendrimers, liposomes, etc.), and inorganic ones (which can be classified according to their core and surface chemical features). Section 3 provides the reader with detailed information on the various prosthetic groups used for radiofluorination, which are mentioned in the first chapter. The methods and techniques used to purify and analyse the radiolabelled nanoplatforms are then reviewed in Section 4 (Figure 1). Based on this overview, we then discuss the scope and limitations of the use of [^18^F]-labelled nanoplatforms and the expectations of their future development toward translational applications. At the end of the document, a list of abbreviations and definitions is provided to ease the reading for researchers from different fields.

## 2. [^18^F]-Radiolabelled Nanoplatforms

### 2.1. Organic Nanoplatforms

The first organic nanoplatforms investigated were natural macromolecules, such as albumin. They display a well-defined molecular structure, and their radiolabelling is now well mastered. This section will focus on statistical organic nano-objects, the physico-chemical descriptors of which, such as composition, size (geometrical and hydrodynamical) or surface charge, are only known as average values with standard deviations. Furthermore, some of these nano-objects (micelles, droplets, etc.) are highly dynamic nanostructures in which the physico-chemical properties depend on pH and ionic strength, among other biological parameters.

#### 2.1.1. Fluxional Micelles, Nanoemulsions, and Bubbles

*Perfluoro gas bubbles* encapsulated in self-assembled lipids, and albumin, were developed for ultrasound imaging. A higher ligand density at the surface of the bubbles was determined when albumin was used for their stabilization [7]. After functionalization with anti-VEGFR2 antibody, labelling of the albumin shell with [^18^F]-succinimidylfluorobenzoate ([^18^F]-SFB **38**, see Section 3.2.3) allowed to study their trafficking in vivo by both PET and echography. Given their size (from 0.5 to 6 μm, with an average hydrodynamic diameter of 1.98 μm), these bubbles were easily trapped in the lungs, then accumulated in the liver. Lower accumulation in liver was achieved upon conjugation with anti-VEGFR2 antibody to target breast cancer (17.7% ID/g compared to 41.8% ID/g). This illustrated the potential of such bubbles for the delivery of therapeutic agents to cancer tumours (ultrasound-assisted therapy).

*Perfluorocarbon* (PFCD) *droplets*, also used in echography, further exhibit exquisite properties for ^19^F MRI and as drug delivery vehicles [8,9]. Nanoemulsions of perfluorohexane stabilized by a perfluoro-surfactant (average size 175 nm) and nanoemulsions of perfluorooctylbromide stabilized by lipids (average size 260 nm) were radiolabelled to investigate their biodistribution in normal mice by PET [10]. This evidenced different tissue uptake and clearance pathways for the two nanoemulsions.

*Nanoemulsions* of vitamin E stabilized by sphingomyelin and stearylamine are a recent class of drug delivery vehicles suited for the transport of lipophilic drugs (e.g., curcumin and resveratrol) [11]. Their ease of modification allows incorporation of vectors, and functionalization towards [^18^F]-labelling. For example, the lipophilic C_18_ chain of SH-PEG_12_-C_18_ and RPMpeptide-PEG_8_-C_18_ (RPM peptide = CPIEDRPMC) allowed their integration into the nanoemulsion. Given their amphiphilic nature, they serve as additional surfactants affording surface RPM epetide as a vector and SH functions for further derivatization. The labelling process with [^18^F]-FBEM (4-[^18^F]fluorobenzamido-N-ethylmaleimide, see Section 3.3.2) was successfully accomplished utilizing the SH groups exposed at the surface of the nanoemulsion droplets. This nano-object (hydrodynamic size = 149 nm, ζ potential = +37 mV) produces a detectable signal for PET imaging 2 h post-injection. The pharmacokinetics and excretion routes were analysed through ex vivo biodistribution and in vivo imaging studies both in healthy and metastatic colorectal tumour-grafted mice. The results confirmed the benefits of surface decoration with RPM to target metastatic tumours, which were already observed with RPM-modified chitosan-stearic micelles [12,13].

*Self-assembly of amphiphilic molecules* into direct micelles allows the encapsulation of lipophilic drugs. However, micelles form only when the concentration of the amphiphile is above a certain threshold (the critical micellar concentration, c.m.c) and their stability depends on ionic strength and pH. Their persistence in vivo and thus their usefulness as delivery vehicles has been questioned, promoting the development of their radiolabelling to investigate their fate in vivo.

As an example, polyethylene glycol (PEG) 3400–distearoylphosphatidylethanolamine (DSPE) spontaneously form micelles (at concentrations above 10 μM) of approx. 10 nm diameter [14]. These micelles were tested as potential cargo for [^18^F]-dasatinib in comparison to phosphatidyl:cholesterol liposomes (see Section 2.1.1). After loading, the average micellar size increased to 50 nm. Loading densities of [^18^F]-dasatinib [drug/lipid (mol/mol) ratio] were found to be 1/233 and 1/2000 (mol/mol) for micelles and liposomes, respectively. PET investigations showed that the uptake in tumours (glioma-bearing mice), was significantly higher for drug-loaded micelles compared to liposomes. This result was explained by the smaller size of the micelles in comparison to that of the liposomes (120 nm). Local concentrations of the amphiphile were estimated to be close to that of the c.m.c., suggesting the easy release of the drug in vivo.

Triglyceride-rich lipoprotein self-assemblies (chylomicron nanoparticles) that display a lipophilic core have inspired the development of chylomicron-like radiotracers. For example, encapsulation of a fluorescent, [^18^F]-radiolabelled fatty acid inside chylomicron nanoparticles (average geometrical size 156 nm, and hydrodynamic diameter 164 nm) was performed. It allowed to study the brown adipose tissue in insulin-resistant conditions by a combination of fluorescence and PET imaging and generally outperformed [^18^F]-FDG [15].

Inorganic nanoparticles have also been encapsulated inside micelles to improve their biocompatibility and pharmacokinetics. Thus, encapsulation of radiolabelled THCPSi nanoparticles has been studied [16]. In an improved approach, late radiolabelling of the surfactant of the micelles encapsulating quantum dots was also investigated [17].

**Figure 2:**
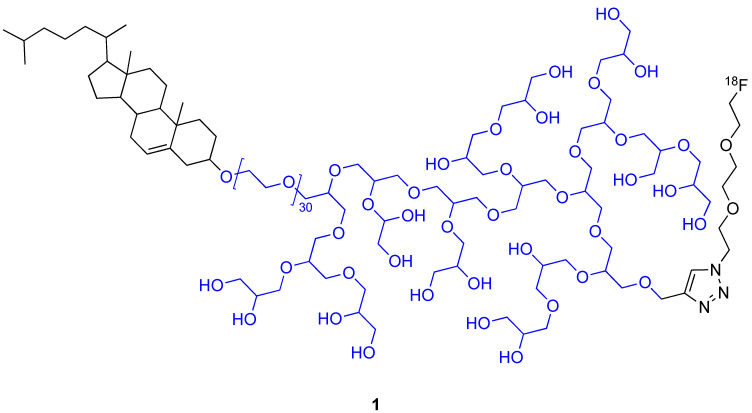
Topological description of the hyperbranched polyether.

Self-assembly of radiolabelled macromolecules enhances their in vivo imaging by PET owing to high [^18^F] concentration. This is exemplified by the investigation of the biodistribution of [^18^F]-hyperbranched polyether ligands **1** (Figure 2). Their self-assembly into micelles led to locally high concentration of [^18^F]. PET imaging showed the rapid renal excretion of these polyethers with full clearance reached after 1 h [18]. This feature is a high added value when efficient self-assembly uptake by tumour cells is achieved, [19,20] and it was recently applied to detect and monitor apoptosis in vivo [21].

**Figure 3:**
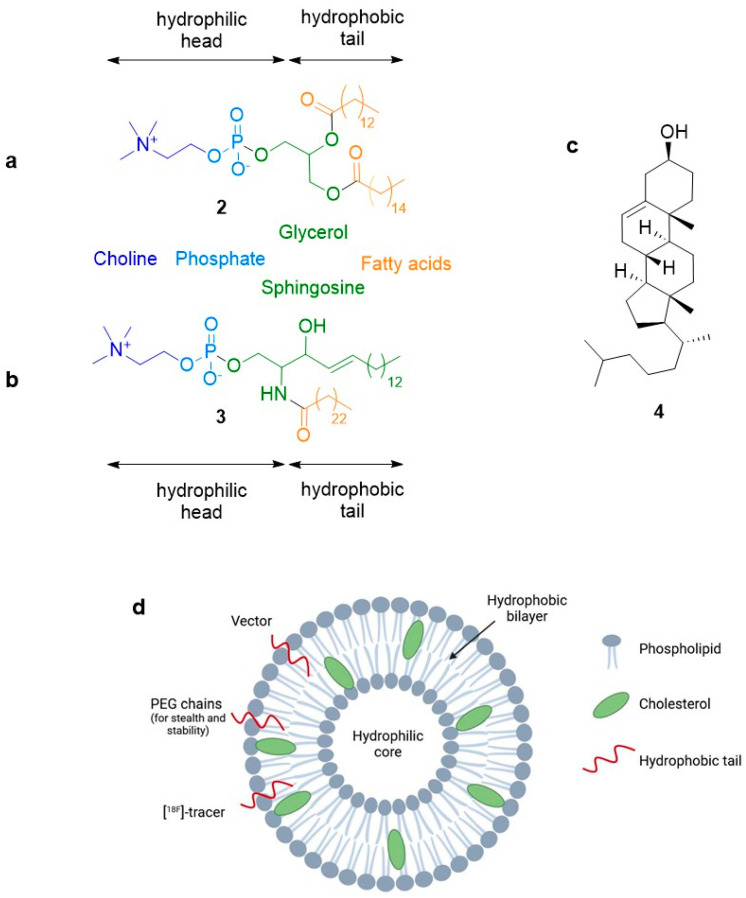
(**a**) Structural illustration of glycerolphospholipid. R_1_ and R_2_ can be saturated or unsaturated fatty acids, such as decanoic acid, lauric acid, palmitic acid, oleic acid, myristic acid, stearic acid, and erucic acid. R_3_ can be phosphatidylcholine, phosphatidyl ethanolamine, phosphatidyl serine, phosphatidyl inositols, phosphatidic acid, phosphatidylglycerol, and cardiolipin; (**b**) structure of sphingomyelin; (**c**) structure of cholesterol; and (**d**) schematic representation of a bilayer liposome and possible surface modifications (created with Biorender).

*Liposomes* are vesicles formed by the self-assembly of phospholipids, such as **2** and **3**, into spherical bilayers (see Figure 3) or onion-like multilayers. A wide range of phospholipids, natural or synthetic, can be used to afford liposomes of different properties (size, rigidity, fluidity, permeability, stability, and electrical charge). A surfactant can be added to the liposome formulation to increase its deformability. Cholesterol **4** is generally added to increase their fluidity (Figure 3c). As cell membrane mimics, liposomes are considerably more robust nano-objects than micelles. They can accommodate a large range of hydrophilic (in their aqueous core) or hydrophobic (in the lipidic bilayer) molecules. Liposomes can also be modified by incorporation of various polymers with lipidic segments in the membrane (Figure 3d). For example, linear or hyperbranched PEG polymers are frequently added to improve their pharmacokinetic profiles [22,23]. Their targeting can also be envisaged by the same method. Given their ease of preparation and functionalization, they are the first nano-carriers used for clinical applications and their development largely contributed to the development of nanomedicine. They are now found in many clinical applications [24,25]. These developments have necessitated the study of their trafficking in vivo. Many studies made use of radiolabelling for this purpose [26,27]. Table 1 reports some typical use of their [^18^F]-labelling. Three modes of introduction of the radiolabelled probes can be envisaged for the liposomes: (1) introducing a radiofluorinated cargo in the core; (2) introducing a radiolabelled unit in the bilayer (either a labelled cholesterol derivative or another radiolabelled hydrophobic derivative); and (3) labelling functional groups at the surface of the liposome. The location of the radiolabel is also given in Table 1.

#### 2.1.2. Polymers

Another approach to develop nano-carriers is to form rigid micelles by a careful design of biocompatible block-copolymers integrating both hydrophilic and lipophilic segments in their structure. [^18^F]-labelling has been implemented to study their biodistribution in vivo and especially to check the efficiency of their targeting prior to their application as drug delivery cargo. Block-copolymers studied so far comprise four main categories of polymeric segments: poly-N-(2-hydroxypropyl) methacrylamide (pHPMA), polyesters, polyethers, and polyoxazolines (POX).

##### Poly-N-(2-Hydroxypropyl)Methacrylamide (pHPMA)-Based Macromolecules

**Figure 4:**
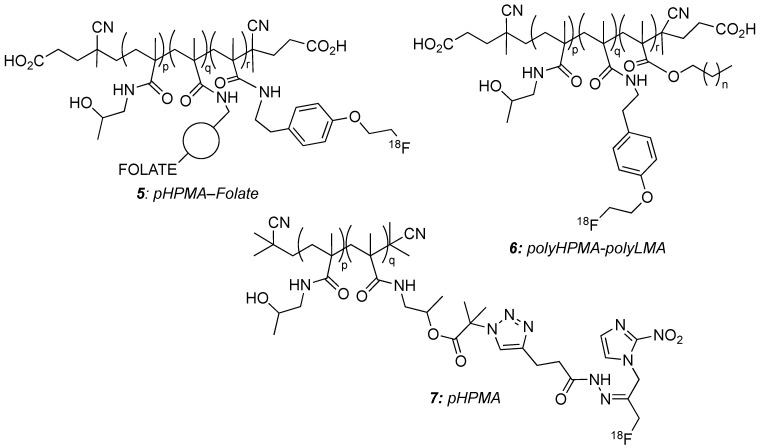
Poly-N-(2-hydroxypropyl)methacrylamide-based macromolecules.

pHPMA (Figure 4) is a non-immunogenic, nontoxic, and usually long-circulating polymer, which spontaneously forms micelles in physiological conditions and allows effective drug delivery. It is already applied in clinical practice [22]. Its conjugation to folic acid was investigated as a possible tool to target cancer cells. Study of the biodistribution of [^18^F]-pHPMA and [^18^F]-pHPMA-folate **5** showed a distinct difference between passive and active targeting, with a faster uptake for the targeted polymer independent of its size (10.5 kDa or 52.5 kDa) [40]. Copolymerization to yield random HMPA/LMA **6** (LMA = laurylmethacrylate) copolymers, or block-copolymers pHPMA-pLMA, was studied to impart lipophilicity to the macromolecule and facilitate its use for the delivery of lipophilic drugs [41,42]. All these copolymers formed stable micelles leading to nanomaterials with hydrodynamic radii of 30–40 nm (random copolymers) to 59–112 nm (block-copolymers) with high stability in serum. After [^18^F]-labelling, the biodistribution of copolymers with different molecular weights (from *ca* 13 to 134 kDa), structures (random or block), and monomer ratios was studied first in healthy rats. The difference was better observed in high molecular weight polymers, with the block-copolymers being much more retained in the liver and spleen than the random ones. Passive targeting of tumours was further assessed, with results being model dependant. The macromolecule architecture had no impact on its accumulation in AT1 prostate carcinoma, but random copolymers were shown to accumulate more than the block-copolymers in Walker-256 mammary carcinoma.

More recently, pHPMA was used as a [^18^F]-nitroimidazole tracer delivery cargo. Thus, **7** exhibits a linear pHPMA chain, which was [^18^F]-labelled with a pH sensitive linker. The study showed the specific release of the [^18^F]-radiotracer in the vicinity of cancer tumours that present lower pH. So, by this means, the uptake of the radiotracer in cancer cells, which grow in acidic conditions, was facilitated [43].

##### Polyester-Based Macromolecules

**Figure 5:**
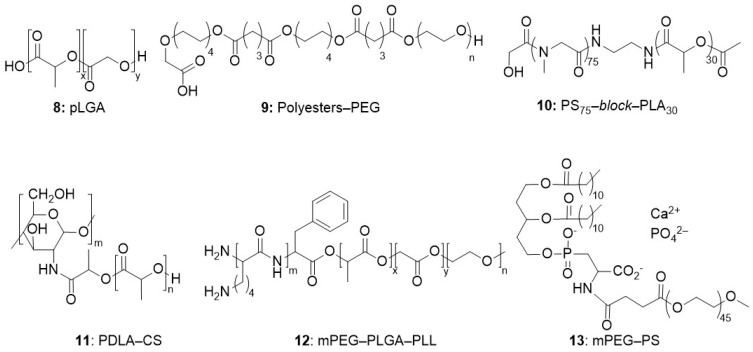
Polyester-based macromolecules.

The polyester segments are often associated to polyether or polyamide segments (Figure 5) to form amphiphilic structures that self-assemble into micelles with low c.m.c. value. The slow biodegradation of the polyester block ensures a continuous delivery of the drug at the site of interest when a proper targeting vector is installed at the surface of the polymeric micelles.

Poly(lactic-co-glycolic acid) (PLGA **8**, see Figure 5) nanoparticles are recognized as drug delivery vehicles capable of transporting a wide range of therapeutic agents (small molecules, proteins, and oligonucleotides, etc.) while protecting them from degradation and clearance. With proper surface modification of the PLGA nanoparticles, their diffusion in the extracellular matrix of the brain has been tuned (in rats) [44]. Given the fast distribution of the nanoparticles in vivo (from minutes to a few hours), radiolabelling with the short-live [^18^F] radioisotope was developed to minimise radiation exposure. Two sizes of polymeric nanoparticles were investigated (147 (±27) nm and 71 (±13) nm geometrical diameters). Nanoparticles were delivered directly and continuously to the rat striatum and their diffusion was monitored by PET. It confirmed that the diffusion region was always spherical, with a larger volume reached by the smaller particles.

A block-copolymer **9** comprising a polyester block (1,8-octanediol/glutaric acid) and a polyether PEG1500 block was designed and studied as a delivery vehicle for the hydrophobic anti-cancer drug Paclitaxel. The particles obtained from this polymeric structure displayed sizes below 200 nm with very low polydispersity index and negative ζ potential (−30 mV). The PEG block is expected to be exposed at the surface of the nanoparticles while the more hydrophobic polyester block accommodates the Paclitaxel in the core of the nanoparticle. After i.v. injection to rats, a low uptake in liver and renal clearance of the nanoparticles was observed by PET for nanoparticles exposing PEG chains at their surface, as expected [45].

Micelles of polylactic acid/polysarcosine (PS-PLA) block-copolymer **10** have also been evaluated as a potential radiotracer for in vivo PET imaging of solid tumours [46]. In this self-assembly, the sarcosine block of the polymers constitutes the hydrophilic, external part of the micelle. Once labelled, a hydrodynamic size of 41.5 nm was measured. Accumulation of the radiotracer in the tumour of HeLa-bearing mice was monitored by PET. A high tumour/muscle signal ratio was evidenced, which was associated to the EPR effect, as well as a low tumour/blood ratio that was associated to the high stability of the micelles in the blood stream and to their slow clearance from blood.

A comparative study was carried out on a series of block-copolymers of various structures: D-polylactide segments grafted on chitosan (PDLA-CS **11**, positively charged in physiological conditions), PEG modified poly(lactic-co-glycolic acid)-b-polylysine (PEG-PLGA-PLL **12**, also positively charged), and PEG-modified phosphatidylserine/calcium phosphate hybrids (PEG-PS-CaP **13**, negatively charged). (Figure 5) These block-copolymers self-assembled into micelles with hydrodynamic sizes and ζ potential values of 120 nm and +16.5 mV, 130 nm and +12.5 mV, and 100 nm and −12.5 mV, respectively [47]. Their biodistribution was followed by PET after injection in tumour-bearing mice. PET imaging revealed the higher impact of the PEG modification over surface charges, which allowed a faster accumulation of the nanoparticles in the tumour and longer residence time in the blood stream.

##### Poly(2-Alkyl-2-Oxazoline)s (POX)

**Figure 6:**
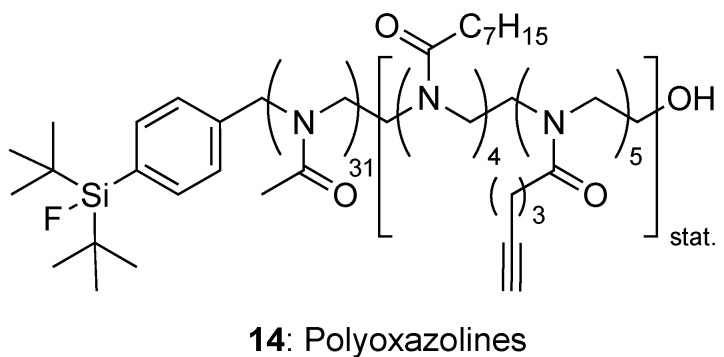
Polyoxazoline-based macromolecules.

POX **14** (Figure 6) are recognized as promising alternatives to PEG with good biocompatibility and stealth. Their easy synthesis by ring-opening polymerization allows the formation of block co-polymers from different oxazoline monomers, and hence a fine tuning of the properties of the final macromolecule. Especially, its hydrophobicity can be adjusted by varying the length of the alkyl residue on the 2-oxazoline monomer.

For example, heptyl- and pentynyl-substituted oxazoline monomers were used to prepare a block-copolymer where the alkyne pendant groups were used to cross-link the polymer chains and stabilize the nanoparticles formed [48]. Furthermore, an organosilicon fluoride acceptor could be used as the initiator in the ROP, allowing in fine the radiolabelling of the nanoparticles by isotope exchange (using a silicon–fluoride acceptor (SiFA) [^18^F]-labelling). Nanoparticles with distinct hydrodynamic diameters (20, 33, 45, and 72 nm) were prepared [49]. Their passive uptake in a murine mammary tumour model were studied depending on particle size. It showed that the 33 nm large nanoparticles outperformed the others, exhibiting the best tumour to muscle ratio.

##### Miscellaneous

**Figure 7:**
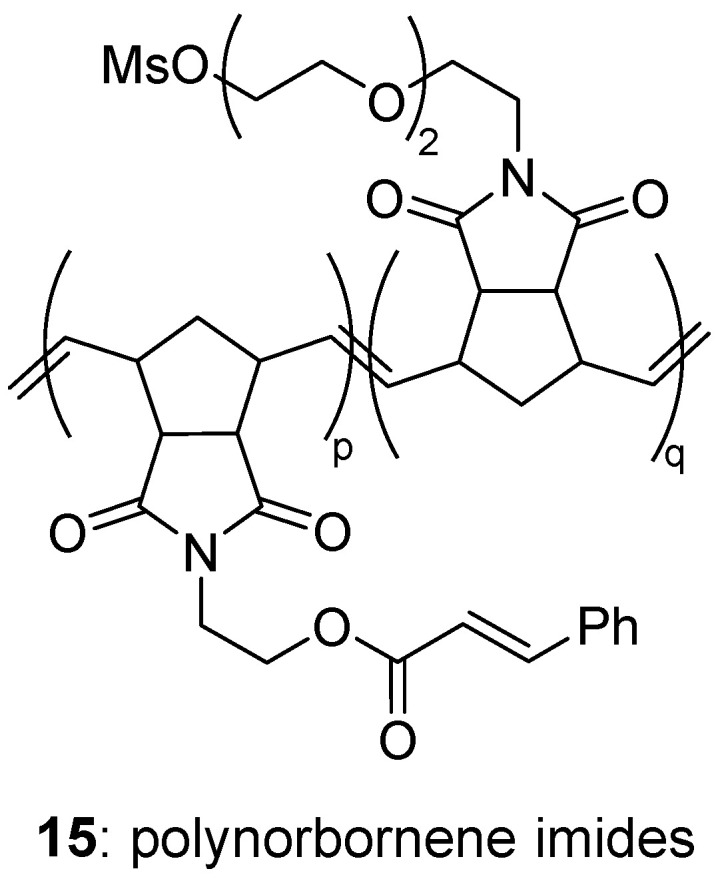
Polynorbornene imide-based macromolecules.

Ring-opening olefin metathesis was applied to a set of two norbornene imides (one with a hydrophobic cinnamoyl group for photocross-linking, and one with a hydrophilic PEG_600_ substituent for biocompatibility) to produce amphiphilic co-polymers **15** with precisely controlled molecular weight (Figure 7). Cross-linking was carried out to stabilize the micelles [50]. Micelles of four different geometrical sizes in the range 13 to 40 nm, and hydrodynamic sizes in the range 47 to 142 nm, respectively, were prepared. Their labelling by [^18^F] was efficiently achieved, offering perspectives for PET imaging, albeit no in vitro nor in vivo study was reported.

#### 2.1.3. Dendrimers and Hyperbranched Molecules

**Figure 8:**
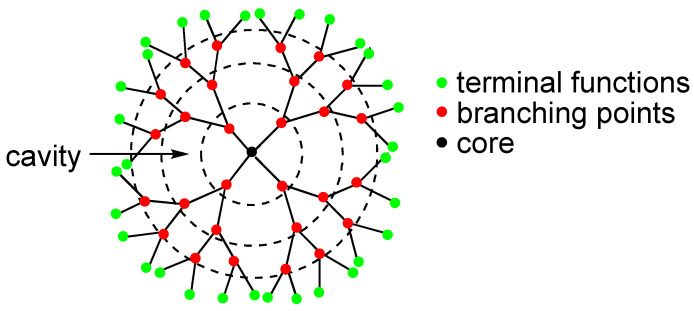
Schematic view of a dendrimer (from the core to the periphery the dashed circles represent 1st, 2nd, and 3rd generations).

Dendrimers are hyperbranched polymers that grow concentrically from a multifunctional core. Each starting branch corresponds to a dendron, itself ramified into new branches (Figure 8).

Dendrimers display a well-defined molecular architecture with many available functional groups located either in their core, along the branches or at the periphery of the different branches, and geometrical and hydrodynamic sizes that directly depend on the generation number (a generation corresponding to the number of iterations of the polymerization). It allows a fine tuning of their solubility and stability in biological medium, biocompatibility, pharmacokinetics, and facile functionalization with targeting ligands, and/or radioelements. They find many applications in nanomedicine as drugs or drug carriers [51].

Radiolabelling of dendrimers was the subject of a review in 2017 [52]. [^18^F]-radiolabelling of three generations of PAMAM (poly(amido amine)) dendrimers grown from a disulfide core was first reported. Conjugation to biotin released the half dendrimers (dendrons), each consisting of 4, 8, or 16 branches, with pendant amino groups that were further derivatized to expose arylboronic groups ready for radiolabelling as aryltrifluoroborates [53]. The impact of the size of the dendron on the biotin/avidin recognition was studied in vitro. PAMAM-NH_2_ dendrimers of sixth generation were later radiolabelled [54] by electrostatic interaction. More recently, it was observed that a hydroxyl-terminated PAMAM dendrimer made of repetitive branching units of methyl acrylate and ethylene diamine, with a molecular weight of ~15,000 Da, was selectively taken up by reactive macrophages/microglia. Its radiolabelling allowed to evaluate its ability to detect innate immune activation in mice following lipopolysaccharide challenge [55]. This family of dendrimers is currently under clinical trial for the study of neuroinflammatory diseases in humans [56].

Interestingly, dendrimers have been used to stabilize inorganic nanoparticles. Radiolabelling of these more complex architectures has been recently reviewed [57]; however there is no mention of [^18^F]-radiolabelling.

### 2.2. Inorganic Nanoplatforms

Like the organic nanoparticles described above, inorganic nanoparticles are radiolabelled first to study their pharmacokinetics and biodistribution in vivo. They occasionally serve as inert carriers of molecular drugs or radiotracers, thus modifying their pharmacokinetics and biodistribution. They can also bring complementary properties to molecular radiotracers anchored on their surface or sequestered in their core. Inorganic nanoparticles can be designed with a mixed core–shell structure. As reported below, the shell might also be implemented to favour surface functionalization. Alternatively, it may be of interest to stabilize the properties of the core (luminescence or magnetism) or to bring a complementary physico-chemical property and afford multimodal platforms (e.g., association between a magnetic core and a luminescent shell). The specific physico-chemical properties of inorganic nanoparticles associated with their ease of functionalization make them ideal nanoplatforms to develop multimodality imaging or theranostic agents. The nanomaterials of interest are hereafter categorized following the nature of the core material that governs their synthetic processes. When available, the main characteristics of the physico-chemistry of their surface are indicated.

#### 2.2.1. Metal Fluorides

Metal fluoride nanoparticles are a first class of studied nanomaterials, particularly those of rare earths, and closely related elements such as yttrium, of the type NaMF_4_ or KMF_4_ (M = Y^3+^, Gd^3+^, Lu^3+^), and MF_3_ (M = Y^3+^, Gd^3+^), or even bismuth. Their [^18^F]-radiolabelling is generally achieved by ion exchange (see Section 3.2.2).

Fluorine displays a strong affinity to rare earth elements, hence the solubility of these fluorine salts in aqueous phases is very low (e.g., the solubility constant of YF_3_ is as low as 8.62 × 10^−21^ (25 °C)) [58], which limits leakage of the heavy elements in vivo.

The exceptional optical and magnetic properties of rare earths, and their high X-ray absorption coefficients make them attractive for a wide range of biomedical applications both for imaging (fluorescence, MRI, and CT) [59] and light-driven therapies (PDT, light activated drug delivery, etc.) [60].

For example, owing to its high magnetic moment, Gd^3+^ in the form of gadolinium complexes is a well-used contrast agent for T1-weighted MRI, making the Gd^3+^/[^18^F] pair a commonly studied combination for multimodal MRI/PET imaging. Thus, GdF_3_ nanoparticles stabilized by human serum albumin (HSA) were developed, with geometric diameter 2.7 ± 0.4 nm, hydrodynamic size of 3.78 ± 0.8 nm, and a ζ potential of −21.00 ± 1.20 mV in agreement with the negative charge of HSA at neutral pH. The use of HSA presented the added advantage of providing a suitable hydrophilicity and biocompatibility to the GdF_3_ core. Given their moderate relaxivity (5.11 mM^−1^·s^−1^), T1-weighted MRI images with significant contrast could only be obtained 24 h after injection, when enough nanoparticles were accumulated in the tumour. Thus, in vivo radiolabelling of these nanoparticles was mandatory to follow their biodistribution in the early hours after injection (<3 h) [61].

**Figure 9:**
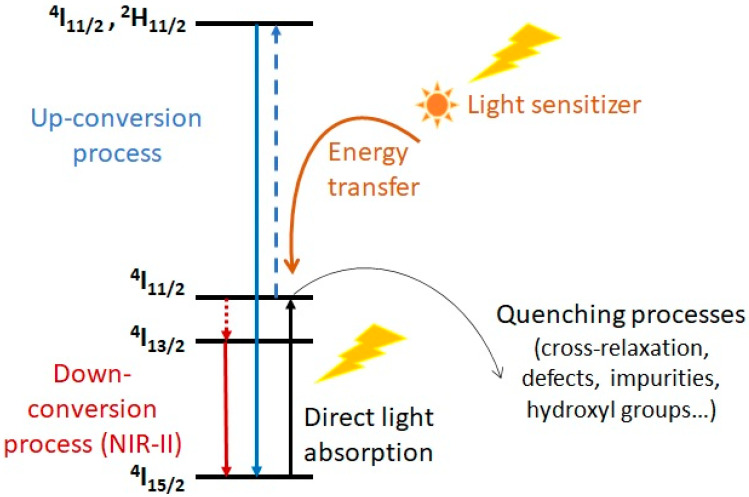
Simplified energy-level diagram depicting the luminescence mechanism of Er^3+^ ion as a typical example.

YF_3_ was also studied given its interest as a Ln^3+^ host matrix to develop up-conversion nanoparticles (UCNP, see Figure 9). Large 5 nm YF_3_ nanocrystals were synthesized in water in the presence of citric acid. They self-assembled into aggregates, the size of which could be adjusted from 30–40 nm to 50–70 nm by fine-tuning the yttrium salt/citrate ratio, reaction temperature, and time. Introduction of a targeting ligand, such as folic acid, helped maintain their size in the 20–30 nm range. Finally, the negative surface charges allowed electrostatic immobilization of doxorubicin, affording nano-objects of total hydrodynamic size of 73 nm and large stability in serum. A good uptake in lymph nodes was reported in mice [62].

The physico-chemical properties of rare earth fluoride nanoparticles can be tuned when doping these host matrices with other Ln^3+^ ions. Two classes of luminescent nanomaterials are distinguished: up-conversion nanoparticles (UCNPs), capable of absorbing light in the NIR region while emitting with a large anti-Stokes shift at much higher energy (e.g., YF_3_ doped with a Ln^3+^ ion such as with Yb^3+^, Tm^3+^, or Ho^3+^, or Er^3+^, KGdF_4_ nanoparticles doped with Yb^3+^ or Tm^3+^ ions [62]), and down-conversion nanoparticles (DCNPs) emitting at lower energy in the NIR-II window (e.g., KGdF_4_ nanoparticles doped with Eu^3+^ ions [59]). The basic principles are exemplified in Figure 9 for Er^3+^ ions.

Recent review articles provide detailed information on UCNPs [60,63] and DCNPs, [64] respectively, as well as on recent developments on nanomaterials exhibiting both properties [65]. These converting properties, associated with narrow emission bandwidths, long fluorescence lifetimes, and photostability, have a wide range of applications in fluorescence biomedical imaging or for the light activation of prodrugs in vivo [66]. This has prompted the study of their pharmacokinetics and biodistribution. It is noteworthy that tracking these nanoparticles in vivo cannot rely on their intrinsic fluorescent properties as depth of penetration at the emitted wavelengths in the tissues is low (<2 cm). This is especially true for nanoparticles with diameters below 10 nm, which is unfortunate as their pharmacokinetics is expected to be more appropriate for bioimaging [67]. Indeed, fluorescence yields decrease with size due to a higher ratio of surface defects and a more efficient quenching by the solvent. As explained above, despite their magnetic properties, monitoring their trafficking in vivo can be performed neither by MRI nor computed tomography (CT) despite their high absorption coefficients because of the low sensitivity of these methods.

Labelling with [^18^F] to perform monitoring by PET imaging is then a good alternative to fluorescence, CT, and MRI imaging to investigate the fate of these nanoparticles in vivo [68].

Based on this approach, the biodistribution of 3.8 nm large [^18^F]-KGdF_4_:Eu nanoparticles, stabilized by oleic acid and coated with polyacrylic acid for hydrophilicity (final hydrodynamic size of 30 nm and ζ potential close to −13 mV in neutral pH), were studied. Efficient uptake in tumours was demonstrated [59]. The low uptake of [^18^F]-fluoride in bones (below 2% ID/g) confirmed that defluorination of the nanoparticles occurred only marginally despite the small size of the nanoparticles that could favour ion exchanges. The same observation (bone uptake below 2% ID/g) was made when NaGdF_4_:Yb,Er nanoparticles (geometrical size 10 nm, coated with citrate ions) were studied [66]. An even lower bone uptake was measured when these nanoparticles were coated with alendronic or nitrilotri(methylphosphonic) acid. Contrarily, a *ca* 10% ID/g in bones was observed for [^18^F]-NaYF_4_:Yb,Er nanoparticles with a Gd^3+^-doped surface aiming at promoting T1-weighted MRI. These nanoparticles displayed a geometrical size of 20 nm, ζ potential of +18 mV, and hydrodynamic size of 25 nm due to their surface coating with acids like folic acid, oleic acid, and aminocaproic acid for specific targeted applications [69]. Large 20 nm NaY_0.2_Gd_0.6_Yb_0.18_Er_0.02_F_4_ nanoparticles with citrate ions coating were also studied in order to develop a nanoprobe for multimodal PET/MR/UCL imaging, with the same result regarding bone uptake [70]. Oleic acid stabilized NaY_0.79_F_4_Yb_0.2_Tm_0.01_ nanoparticles of comparable size, but with a coating of α-cyclodextrin improving hydrophilicity and physiological stability, also showed a similar bone uptake after 2 h of incubation [71]. All these data seem to indicate that radiochemical release may be related to the cation nature (Gd^3+^ versus Y^3+^) or nanoparticle size, rather than to the nanoparticle coating.

A bismuth oxyfluoride matrix was also used to develop UCNPs for multimodal imaging [72]. The [^18^F]-doped bismuth nanoprobe (Na_0.20_Bi_0.80_O_0.35_F_1.91_: 20% Yb, 0.5% Tm) displayed a geometrical size in the range 30–60 nm, ζ potential of ≈0 mV, and a hydrodynamic size around 150 nm. It showed interesting X-ray attenuation characteristics for CT imaging, a good [^18^F]-radiolabelling yield for PET imaging, and UC luminescence properties.

#### 2.2.2. Metal Oxides and Hydroxides

##### Magnetic Nanoparticles

Magnetic nanoparticles (MNPs) are often based on iron oxides, more scarcely on cobalt. Indeed, iron oxide MNPs are non-toxic and cost-effective, and display sufficient magnetization values for biomedical applications. They mainly act as contrast agents for T1- and T2-weighted MRI depending on their size; moreover, they also find applications in thermotherapy, for magnetically driven drug delivery, or to treat anaemia in adults. Here again, as the low sensitivity of MRI does not afford accurate detection, radiolabelling of MNPs is a valued strategy to assess their pharmacokinetics.

**Figure 10:**
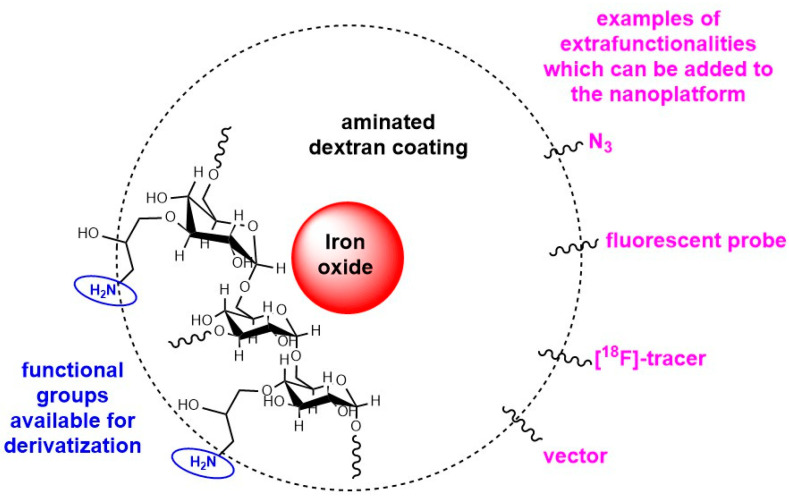
Schematic representation of a CLIO nanoplatform.

Carbohydrate-coated iron oxide nanoparticles have been developed as contrast agents for magnetic resonance imaging of the liver and lymph nodes, and to treat anaemia in adults. They are generally prepared by coprecipitation of Fe^2+^ and Fe^3+^ ions in the aqueous phase in the presence of the carbohydrate polymer. Monocrystalline iron oxide nanoparticles (3–5 nm geometrical size) coated with a cross-linked dextran/epichlorin hydrin layer (final hydrodynamic size *ca* 30 nm) are well-known platforms (referred to as CLIO for Cross-Linked Iron Oxide) that, once treated with ammonia, expose NH_2_ groups ready for derivatization [73] (Figure 10). A first study reported that, once radiolabelled, their detection threshold in vivo by PET was lowered by a factor of 200 in comparison to MRI and 50 in comparison to fluorescence imaging [74]. They were later successfully used to evidence the interest in dual fluorescence-mediated tomography (FMT) and PET when a far-red fluorophore was grafted on the [^18^F]-CLIO platform [75] and to detect macrophages in aortic aneurysms by combining PET and CT imaging modalities [76]. The biodistribution of [^18^F]-radiolabelled CLIO nanoparticles (*ca* 29 ± 3 nm geometrical size) in healthy mice was monitored by micro-PET/CT imaging [77]. It was evidenced that the nanoparticles could be efficiently concentrated to a specific anatomical location by application of an external magnetic field and could also be used for magnetically assisted drug delivery.

[^18^F]-labelled ultrasmall superparamagnetic iron oxide (USPIO) nanoparticles (geometrical size 6.5 ± 0.5 nm), coated by polyacrylic acid and polydopamine layers, were developed as dual-modality PET/MRI nanoprobes. Their derivatization with NH_2_-PEG-C≡CH led to nanoparticles with an average hydrodynamic diameter of 40 nm and ζ potential of −21 mV. It allowed the introduction of the radiolabel, and of an arginine–glycine–aspartic acid (RGD) peptide for targeting by copper-catalyzed alkyne–azide cycloaddition (CuAAC). The final [^18^F]-RGD@USPIO nanoparticles were injected into MDAMB-231-bearing mice showing a three times higher tumour uptake in comparison to the non-vectorized nanoparticles. They were successfully used to monitor in vivo the therapeutic effect of anti-angiogenesis in breast cancer by combining PET and MRI [78]. Dopamine-coated Fe_3_O_4_ nanoparticles, functionalized or not with folic acid for vectorization (geometrical size 6.8 nm, hydrodynamic size *ca* 170 nm, and ζ potential value *ca* + 21 mV) and γ-Fe_2_O_3_ nanoparticles (average geometrical size of 250 nm, ζ potential value −28 mV) coated by PEG_6000_ and dopamine were radiolabelled via derivatization of the NH_2_ groups of the dopamine ligand. The cellular uptake in MCF7 breast tumour cells was studied by measuring the radioactivity level after 1 and 2 h of incubation in vitro. Here again the highest uptake was achieved with the folic acid-modified nanoparticles (e.g., 35% ID/g, 16% ID/g, and *ca* 2% ID/g for folic acid-modified Fe_3_O_4_ nanoparticles, folate-free Fe_3_O_4_ nanoparticles, and free [^18^F]-probe, respectively). These nanostructures showed a dual-modality capability (PET and T2-MRI) [79]. The pendant carboxylic acid functions of a polyacrylic acid layer, used to impart iron oxide nanoparticles (8 nm geometrical size) with high stability in aqueous medium in a large range of ionic strengths (up to 600 mM) and pH values (>4.5), with constant hydrodynamic size of *ca* 30 nm and ζ potential between −47 and −25 mV, were also used for further derivatization and radiolabelling (see Section 3.3.2). PET imaging in mice showed a non-specific accumulation in liver and spleen where significant radioactivity still persisted 4 h post-injection (22.5 ± 1.6 and 9.2 ± 1.5% ID per g, respectively) [80].

MnFe_2_O_4_ and Fe_3_O_4_ nanoparticles produced in organic phase were further coated by an aluminium hydroxide layer to stabilize their magnetic properties, making them efficient MRI contrast agents [81]. Hydrodynamic sizes of 21 nm, and very high positive ζ potential values of +72 mV and +50 mV for MnFe_2_O_4_@Al(OH)_3_ and Fe_3_O_4_@Al(OH)_3_ nanoparticles were reported, respectively. These properties were influenced by both the thickness and the hardness of the Al(OH)_3_ layer, which depend on the synthetic method. The Al(OH)_3_ layer also imparts hydrophilicity to the nanoparticles, highly increasing their colloidal stability in water. It also reacted easily with phosphonate derivatives (e.g., bisphosphonate polyethyleneglycol) and fluoride ions allowing their efficient radiolabelling (reaching 97%). Together, these two properties provide a dual-modality contrast agent for MRI and PET imaging. However, [^18^F]-fluoride leaking was observed in vivo.

Fe_3_O_4_@Al(OH)_3_ nanoaggregates *ca* 250 nm geometrical size, consisting in magnetite cores (average geometrical size of 7 nm) entrapped in a matrix of porous aluminium hydroxide were also reported [82]. Rapid uptake of [^18^F] by the Al(OH)_3_ surface layer allowed their fast and easy radiolabelling.

These [^18^F]-Fe_3_O_4_@Al(OH)_3_ nanoparticles were successfully used to label mouse mesenchymal stem cells and study their biodistribution in healthy C57Bl/6 mice by combining PET and MR imaging [83]. Comparison between labelled and non-labelled nanoparticles showed the interest of combining MRI and PET imaging in order to improve the visualization and quantification of the nanoparticles in cells. Magnetic cores with a luminescent NaYF_4_ (Yb, Tm) shell were also developed and radiolabelled to afford multimodal nanomaterials associating MRI, PET, and fluorescence imaging. For example, core–shell nanoparticles with various cation dopants, notably Co_0.16_Fe_2.84_O_4_@NaYF_4_ (Yb, Er) and Fe_3_O_4_@NaYF_4_ (Yb, Tm), stabilized by bisphosphonate polyethyleneglycol conjugates were prepared. They exhibited high transverse relaxivity for MRI, high affinity for [^18^F]-fluoride for PET imaging, and fluorescent emissions from 500 to 800 nm under excitation at 980 nm [84].

##### Semi-Conducting Nanoparticles

This class of nanomaterials is widely used in many fields, such as electronics, sensors, catalysis, cosmetics, food additives, and medicine owing to their photoactivity and redox properties.

Titanium dioxide (TiO_2_) nanoparticles have been radiolabelled to study their biodistribution in rats both after i.v. and oral administration. The fate of nanoparticles with geometrical size 7–8 nm and hydrodynamic size 12 nm was followed up to 8 h after administration to the animals by combined PET-CT imaging. Preferential accumulation in the liver and a urinary excretion path were reported. Long after i.v. injection, radioactivity could be detected in the bones suggesting [^18^F]-fluoride leakage [85]. After oral administration, the labelled nanoparticles stayed almost quantitatively in the upper gastrointestinal tract. However, a slow and progressive accumulation in other organs (mainly the small intestine, the bones, and the bladder) was also observed. It is noteworthy that only few studies deal with the biodistribution and pharmacokinetics of TiO_2_ nanoparticles, despite the fact that they are now used in many applications owing to their photoactivity [86,87] and are produced industrially on a large scale.

Two kinds of commercially available zinc oxide (ZnO) nanoparticles [88], with indicated geometrical sizes of 20 and 100 nm, respectively, were traced after their oral administration to mice. For this purpose, the nanoparticles were incubated with 6-hexynoic acid. The alkyne group could further undergo a CuAAC with a [^18^F]-labelled azide. After surface modification, the hydrodynamic sizes of the nanoparticles were *ca* 25 nm and 79 nm, for the 20 and 100 nm nanoparticles, respectively. The study showed that the nanoscale ZnO particles were retained longer in the gastrointestinal track in comparison to the sub-micrometer ones. The nanoparticles were not detected in other organs.

Ceria (CeO_2_) nanoparticles functionalized with 3-aminopropyltriethoxysilane (geometrical size 5.6 nm, ζ potential +18.5 mV) were radiolabelled by coupling the amino pendant groups with [^18^F]-SFB. A geometrical size of 5.6 nm is reported, and a ζ potential of +18.5 mV, in agreement with the presence of amino surface groups, partially protonated in water at neutral pH. After i.v. injection in rats, the nanoparticles accumulated mainly in the liver (6% ID/g), lung, spleen, and kidneys (*ca* 2% ID/g) where the radioactivity level stayed constant along the *ca* 2 h monitoring by PET. Interestingly, it was evidenced that the very high level of radioactivity in the bladder and urine was due to soluble metabolites of the nanoparticles, not to the nanoparticles themselves [89].

Efficient radiolabelling of Y_2_O_3_ nanosheets was also reported in the presence or not of F127 as a surfactant but these were not used for in vivo studies [58].

##### Miscellaneous

Naked alumina (Al_2_O_3_) nanoparticles (5.5 nm geometrical size, 9 nm hydrodynamic size) were used without any surface modification to demonstrate the possibility to perform a direct radiolabelling by a ^18^O(p,n)^18^F nuclear reaction (see Section 3.2.1 for details), and explore their in vivo biodistribution in rats [90]. Commercial alumina nanoparticles of various sizes (see Table 2) were also [^18^F]-labelled by the same method [91].

Aluminium hydroxide (Al(OH)_3_) nanoparticles with hydrodynamic size 1211 (±64) nm (Alhydrogel™) have been radiolabelled by incubation with [^18^F]-fluoride [92]. Given the recognized biocompatibility of aluminium hydrogels (e.g., used as adjuvants in vaccines), this prompted the development of aluminium hydroxide shells to facilitate the labelling of magnetic nanoparticles (see Section 2.2.2, refs. [81,82]) for use in MRI. After intramuscular and sub-cutaneous administration of the [^18^F]-Al(OH)_3_ nanoparticles to mice, it was observed that all radioactivity remained at the site of injection over the 4 h period of monitoring. Contrarily, i.v. injection was followed by fast uptake in the liver and spleen, and by renal excretion.

Efficient radiolabelling of Y(OH)_3_ and Gd(OH)_3_ nanoparticles of large sizes and non-spherical shapes was also reported but these were not involved in any in vivo study [58].

Hydroxyapatite (Ca_10_(PO_4_)_6_(OH)_2_, -HA-) nanoparticles of various sizes, shapes, and surface coatings (see Table 3) were radiolabelled by incubation with [^18^F]-fluoride [92]. In vivo study (in mice) showed different biodistributions of the nanoparticles depending on the administration route (i.v., intramuscular or sub-cutaneous) and aggregation state of the nanoparticles (the more aggregated being expectedly more trapped in the lungs). Interestingly, no radioactivity was observed in joints and bones, indicating that leakage of [^18^F]-fluoride was below detection limit. Similarly, HA layers grown on top of MnFe_2_O_4_@Al(OH)_3_ and Fe_3_O_4_@Al(OH)_3_ nanoparticles were radiolabelled [93]. Interestingly, the Al(OH)_3_ layer of the template material was removed during the formation of the hydroxyapatite shell leading to MnFe_2_O_4_@HA and Fe_3_O_4_@HA nanoparticles with rod-like shapes (60.3 nm × 29.7 nm large for Fe_3_O_4_@HA, and almost twice as much for MnFe_2_O_4_@HA nanoparticles). The nanoparticles were stabilized by coordination of a bisphosphonate PEG-Me polymer on the HA layer leading to final hydrodynamic sizes of 50.7 nm and 60.3 nm, respectively. In this case, despite the presence of this surface ligand, high radiolabelling yields could be achieved.

#### 2.2.3. Metal Sulfides, Selenides, and Tellurides

To the best of our knowledge, no metal sulfide nanoparticle has ever been radiolabelled by ^18^F. However, metal sulfide, especially ZnS, is present at the surface of many quantum dots (QDs), such as cadmium selenide or telluride nanoparticles. These well-known inorganic fluorescent nanoparticles find applications in many fields from optoelectronic devices [94] to bioimaging [95,96], owing to their brightness, sharp emission, low blinking, and long photostability. However, to preserve the excellent quantum yield of CdSe and CdTe nanocrystals, a shell of a semi-conducting material with a larger band gap should be present at the surface to confine the excited electrons within the core of the nanoparticle. ZnS has been largely used for this purpose.

Surprisingly, despite the huge amount of QDs produced and used in biomedicine since their discovery in the 1980s [96], few papers report their radiolabelling with ^18^F. One such example concerns commercially available, amine-modified, CdTe/ZnS nanoparticles (QD705, Quantum Dot Corp., Hayward, CA, USA) that were developed as potential dual-targeting, dual-modality imaging probes [97]. Thus, their surface was functionalized with a synthetic peptide combining an RGD motif (cyclo(RGDYK)) and a truncated bombesin sequence (7–14 BBN). This allowed the recognition of both integrin αvβ3, overexpressed in a variety of solid tumour types, and gastrin-releasing peptide receptors (GRPRs). Their hydrodynamic size was 123.5 nm. Radiolabelling with 4-nitrophenyl 2-[^18^F]fluoropropionate (see Section 3.3.2) provided a dual-targeted PET/NIRF imaging probe. Its efficiency was tested in mice. Clear delineation of the tumour site was possible by PET 30 min after injection, and by NIRF 1 h after injection.

**Figure 11:**
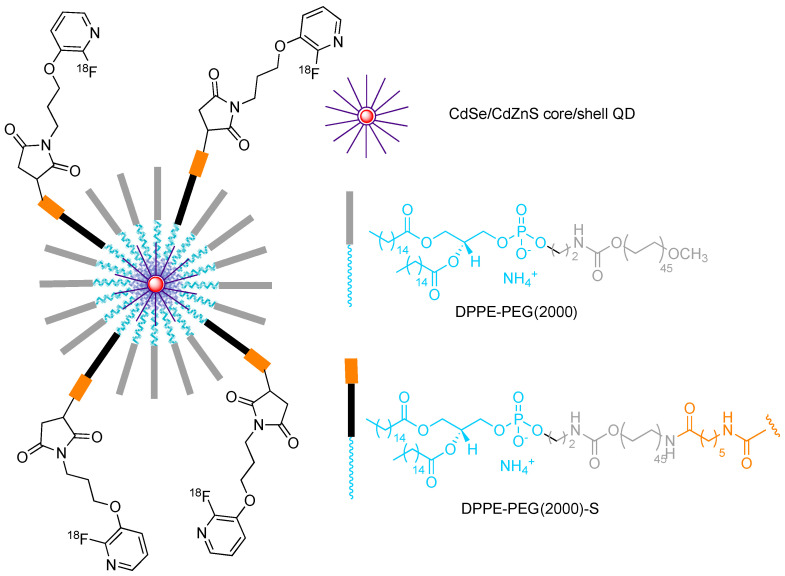
[^18^F]-labelled PEG-phospholipid QD micelle [16].

CdSe/CdZnS nanoparticles (6–8 nm geometrical diameter) stabilized by modified phospholipids, exposing thiol end-groups for further derivatization and radiolabelling were also studied [16] (see Figure 11). This labelling strategy was used to investigate the dynamics of the in vivo biodistribution of non-targeted QDs. No renal clearance was observed, which is in agreement with their large diameter (hydrodynamic size after coating > 20 nm). Interestingly, a few degradation products were observed, which were excreted after 90 min, while the QDs remained in the blood or accumulated in liver and spleen. This study evidenced a prolonged circulation in the blood, with a half-residence time in the order of 2 h.

Presently, a wide range of QDs are commercially available with different pending functions allowing their conjugation with [^18^F]-prosthetic groups such as, e.g., [^18^F]-fluoroethyltosylate [98].

#### 2.2.4. Zerovalent Metal Nanoparticles

Among zerovalent nanoparticles, gold nanoparticles have been the most studied for [^18^F]-radiolabelling. This is in line with the large range of applications offered by these nanoparticles in the biomedical field owing to their biocompatibility [99], ease of synthesis [100], and surface functionalization [101,102] associated with unique physico-chemical properties. Indeed, they display outstanding optical properties that can be tuned by adjusting their size and shape [103], large X-ray extinction coefficient [104], and potentially magnetic properties when their size and surface coating are properly adjusted [105]. Thus, they have been developed as colorimetric sensors [106], optical imaging agents [107,108] for phototherapy [109], and photoactivated antimicrobial applications [110,111]. However, their applications also encompass photoacoustic imaging [112], magnetic resonance imaging [105], radiotherapy (as radiosensitizers) [104,113], and theranostics [114,115]. They can also simply be used as drug delivery vehicles [116,117]. More recently, their promising effect on cell differentiation has also been acknowledged [118].

A first example of gold nanoparticles, [^18^F]-radiolabelling was achieved to study their biodistribution and in vivo pharmacokinetics. For example, 12 nm large gold nanoparticles designed for the treatment of Alzheimer’s disease have been studied. These nanoparticles, prepared by the Turkevich method [119], were functionalized by a combination of CLPFFD and CK peptides that anchored strongly to the gold surface owing to thiol groups of the cysteine residue, while offering available amine functions for further conjugation with a [^18^F]-probe [120]. The final object displayed a hydrodynamic size of *ca* 23 nm and a ζ potential of −40 mV in plasma. A total of 2 h after injection in rats, only traces of the nanoparticles could be detected in the brain (<0.1% ID/g). Rather, a progressive accumulation in liver and spleen was observed and correlated to the size and negative surface charge of these nanoparticles. A biliary excretion path was suggested based on the radioactivity level measured in the intestine, but the highest radioactivity level was measured in the bladder and urine indicating that the main excretion path was urinary (supposing no detachment of the [^18^F]-probe from the nanoparticles). Radiolabelling of much smaller gold nanoparticles (3 nm geometrical size), prepared by a modified Brust–Schiffrin method [121], with a surface decorated by a combination of pegylated thiols to ensure their stability in aqueous phase and promote stealth, showed a slightly higher brain uptake (>0.13% ID/g 2 h after injection in rats) [122].

Radiolabelling was also envisaged to afford theranostic nanoplatforms. Gold nanoparticles with geometrical size between 15 and 40 nm (as determined by scanning electron microscopy) were produced by a modified Brust method [123] in the presence of [^18^F]-FDG-cysteamine adduct, which anchors to the gold nanoparticles surface via the thiol groups of the cysteamine. The OH groups of FDG were then used for conjugation with the anti-metadherin (anti-MTDH) antibody, specific for MTDH, a surface protein overexpressed in breast cancer cells. A slight decrease in ζ potential value from −13 mV for the gold-FDG nanoparticles to −18 mV for the final object is reported, showing an increased stability after conjugation with the targeting antibody. Injection of the gold nanoparticles in rats confirmed their potential for PET/CT imaging and their efficient targeting, prompting their development as radiosensitizers for the treatment of breast cancer.

Gold nanoparticles have also been chosen as representative objects to demonstrate the effectiveness of new labelling techniques [124]. Interestingly, Pd nanosheets have also been used for this purpose [125]. This is, to the best of our knowledge, the sole example of radiolabelling of zerovalent nanoparticles other than gold ones. These examples are discussed in Section 3.3.2.

#### 2.2.5. C- and Si-Based Nanoparticles

Carbon- and silicon-based nanomaterials are attractive owing to their large availability, low cost, and limited toxicity. They display interesting properties for biomedical applications as depicted below.

**Figure 12:**
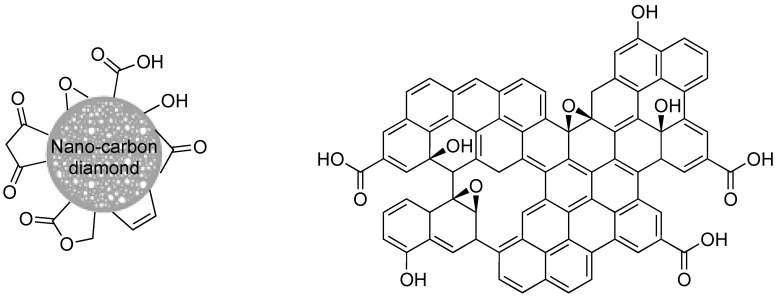
Schematic representation of functional groups at the surface of nanodiamonds and exfoliated n-GO (not at scale).

*Carbon derivatives*. Carbon nanotubes, graphene, fullerenes, or nanodiamonds have been studied as potential platforms for theranostic applications [126], including delivery of radioisotopes for cancer therapy [127]. Being naturally hydrophobic and chemically rather inert, carbon-based nanomaterials are generally partially oxidized to afford surface OH, COOH, and/or epoxy groups to allow their functionalization (Figure 12).

**Figure 13:**
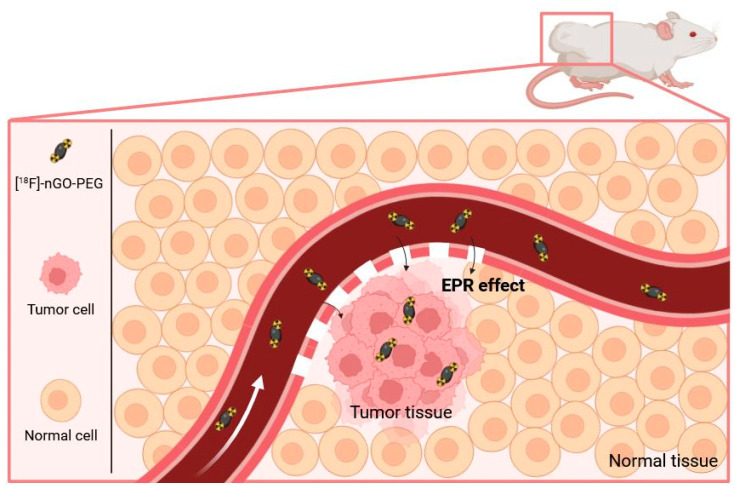
Illustration of the passive accumulation of ^18^F-nGO-PEG in a tumour-bearing murine model, achieved by the extravasation of nanoparticles through the increased permeability of the tumour vasculature (EPR effect). Created with Biorender.

In the case of nanodiamonds, this oxidation step may also be important to remove the sp^2^ carbon derivatives, such as graphite, that may form during their synthesis and in which the nanodiamonds may be embedded. The surface of the nanodiamonds can then be easily modified [128], making them especially interesting for applications in fluorescence and magnetic resonance imaging, as well as in drug delivery [129]. Concerning radiofluorination, taking advantage of the exposed OH groups, 7 nm large (geometrical size) nanodiamonds were reacted with ω-aminopropyltriethoxysilane to finally display amino groups at their surface. The latter were further conjugated with [^18^F]-SFB. Partial aggregation of the nanodiamonds into larger objects (hydrodynamic diameter 680 nm) was observed. These aggregates mainly accumulated in the lungs, and less in liver and spleen after i.v. injection in rats. The addition of 5% Tween80 or 5% PEG_8000_ surfactant had no major effect on their biodistribution, but tended to increase their bioavailability in blood. Contrarily, the smaller nanoparticles or aggregates that could be recovered after filtration (0.45 μm cut-off) were not detected in lungs nor spleen, and only traces could be detected in the liver [130]. This study confirmed the strong impact of the hydrodynamic size of nano-objects on biodistribution, and points out the need for a careful design of surface functionalization to control this parameter in physiological media. Nano-graphene oxide (nGO) was also studied, with the idea of developing a new radiotracer platform [131]. Disk-like nGO platelets were first prepared by exfoliation. They displayed a 50 nm large geometrical diameter in average, and a ζ potential of −70 mV due to the presence of ionised carboxylic acid functions at their surface. These carboxylic acid functions were then used to introduce PEG chains via amidic coupling with NH_2_-PEG-NH_2_ and ensure a good dispersion in aqueous phases such as PBS and DMEM. Accordingly, the ζ potential value increased (−48 mV). The [^18^F]-radiolabelled nanomaterial displayed uptakes of 14% and 8% of the injected dose in MCF7 and CT26 cells after 2 h, respectively. The biodistribution in subcutaneous tumour-bearing CT-26 mice showed a passive accumulation of the pegylated radiotracer in the tumour attributed to EPR effect (Figure 13), but with a low standardized uptake value (SUV) of 0.2109 after 1 h. In comparison, [^18^F]-fluoride and [^18^F]-nGO displayed SUV values of 0 and 0.0912, respectively, pointing out once again the positive role of PEG chains in increasing the blood bioavailability of the nanomaterials. Thus, with proper surface tailoring, nGO sheets could be used for the early detection of tumours in bioimaging or to assist in drug delivery.

An attempt to label active carbon was also briefly reported [58], but to the best of our knowledge, no work reports the [^18^F]-radiolabelling of other important carbon-based nanomaterials, such as fullerenes, carbon nano-onions, and carbon nanotubes, despite their growing interest in the biomedical field [132].

**Figure 14:**
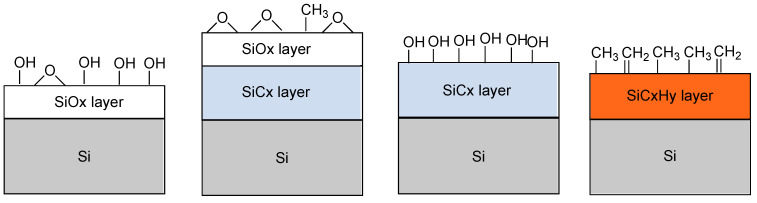
Schematic representation of the chemical groups at the surface of silicon nanomaterials depending on the preparation method.

*Silicon derivatives*. Silicon nanoparticles also present interesting fluorescence properties with long-lived excited states, high brightness, and stability, which make them suitable for the design of fluorescence imaging probes. When porous, they are promising vehicles for the delivery of anti-cancer drugs [133]. To facilitate its functionalization, the silicon surface can be treated to provide a silicon oxide layer with reactive surface OH groups, or a silicon carbide layer with a variety of activable hydroxyl groups, or a silicon hydrocarbide layer with methyl and methylene surface groups (Figure 14) [134]. The main characteristics of the [^18^F]-radiolabelled nanomaterials studied are reported in Table 4.

Porous silicon nanoparticles with SiOx (TOPSi), SiCx (TCPSi), and SiCxHy (THCPSi) surfaces have been compared [135]. After radiolabelling by direct incubation with [^18^F]-fluoride (see details in Section 3.2.2), it was demonstrated that only [^18^F]-THCPSi and [^18^F]-TCPSi displayed sufficient plasma stability (of the radiolabelling) for administration. Finally, only [^18^F]-THCPSi nanoparticles were studied in vivo. Hence, THCPSi nanoparticles of 142 nm geometrical sizes were assessed as potential carriers for oral drug delivery [136]. Indeed, the average pore diameter of the particles (9.0 nm), high specific surface area (202 m^2^/g), and the pore volume (0.51 cm^3^/g) are compatible with drug delivery applications. Their surface was modified with fluorescein isothiocyanate for in vitro studies and [^18^F]-labelled to allow in vivo biodistribution assessment in rats. As THCPSi nanoparticles tend to aggregate in aqueous media (especially in plasma), dispersion of the nanoparticles was ensured by addition of polyethyleneglycol-15-hydroxystearate surfactant. The ζ potential value was close to −30 mV. Oral administration to rats showed that the nanoparticles did not cross the intestinal wall, as only a negligible amount of radioactivity was detected in the blood or organs outside the gastrointestinal tract. Moreover, i.v. injection led to accumulation of the nanoparticles in spleen and liver (*ca* 14% and 7% ID/g after 30 min, respectively). THCPSi nanoparticles with higher specific surface area (323 (±3) m^2^/g) but lower pore volume (0.48 (±0.01) cm^3^/g) and pore diameter (7.4 (±0.2) nm) were also studied [137]. After radiolabelling, these nanoparticles were coated by self-assembly with a fungal protein leading to very stable suspensions both in PBS and plasma. Upon coating the hydrodynamic size increased from 215 to 324 nm and the average ζ potential increased from −33 to −21 mV. After i.v. injection to rats, the highest uptake of the nanoparticles was seen in the liver and spleen (*ca* 10 and 9% ID/g after 30 min, respectively), and in the lungs (*ca* 2% ID/g after 30 min). Less lung uptake was observed in comparison with uncoated THCPSi nanoparticles, indicating that the protein coating efficiently prevented in vivo aggregation of the nanoparticles. It was also noted that this coating prevented [^18^F]-fluoride leakage as no radioactivity could be detected in the bones. Similarly, THCPSi nanoparticles encapsulated into solid lipids frequently used as nano-carriers (glyceryl monostearate and L-α-phosphatidyl choline) were investigated [17]. They displayed a large geometrical size distribution centred on 190 (±40) nm. After coating, the hydrodynamic size was 193 (±30) nm and the average ζ potential −40 mV. When intravenously injected to tumour-bearing mice, the nanoparticles accumulated mainly in the liver and spleen (*ca* 70 and 40% ID/g after 30 min, respectively), precluding their efficiency as drug delivery vehicles based on the EPR effect.

**Figure 15:**
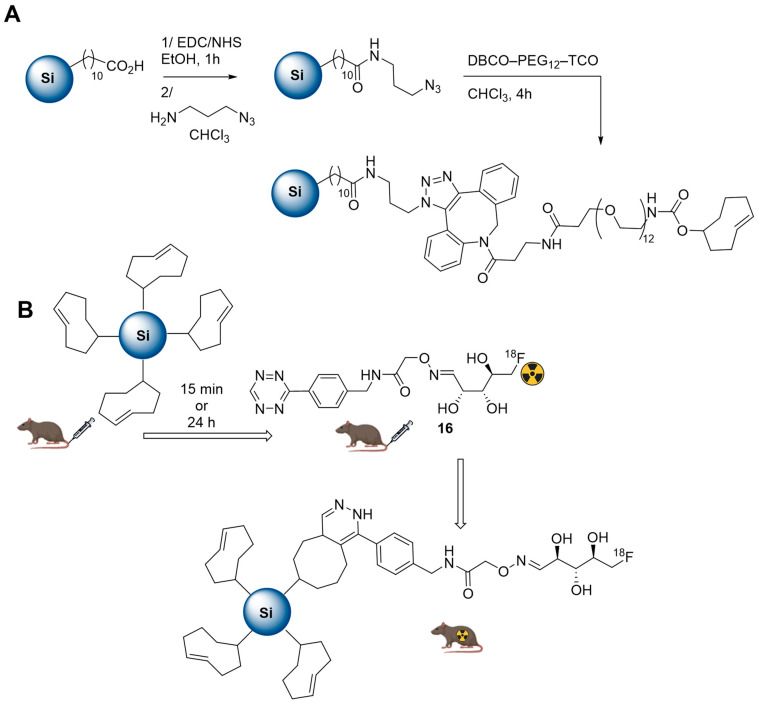
(**A**) Preparation of TCO-THCPSi-nanoparticles and (**B**) description of the pre-targeted methodology developed in [138] for tracing the nanoparticles in healthy mice: TCO-THCPSi-nanoparticles were administered 15 min or 24 h before the i.v. injection of the [^18^F]-probe.

The biodistribution of THCPSi nanoparticles in rats was also studied according to a pre-targeting methodology, were radiolabelling occurs in vivo. For this purpose, THCPSi nanoparticles were surface modified with PEG chains bearing terminal *trans*-cyclooctene functional groups following the strategy outlined in Figure 15. Finally, a treatment with silver nitrate was performed to increase their stability in aqueous phases. Their fast and efficient reaction with a [^18^F]-radiotracer **16** bearing a tetrazine function confirmed the accumulation of the nanoparticles in liver and spleen.

*Silica nanoparticles*. Well-defined silica nanomaterials are present in many fields such agriculture, environmental remediation, and the food industry given their low toxicity. They are also well used in medicine [139] owing to their ease of synthesis and functionalization [140,141]. Mesoporous silica nanoparticles (MSNPs) are particularly interesting as cargo nanomaterials [142].

Nanostructured hydrophilic fumed silica (Aerosil^®^200) was surface-functionalized with 3-aminopropyl-triethoxysilane to impart solubility in the aqueous phase and allow coupling with [^18^F]-SFB. It is noteworthy that fumed silica generally consists in chain-like assemblies of nanoparticles. The dispersion of the nanoparticles improved upon functionalization as the specific surface area increased from 200 m^2^/g to 325 m^2^/g. Nevertheless, the radiolabelled nanomaterial still displayed a broad distribution of geometrical sizes (from 30 to 130 nm). The hydrodynamic size was *ca* 400 (±80) nm and the ζ potential value −21 (±5) mV at neutral pH. After i.v. injection in mice, these nanoparticles accumulated in the spleen and lungs, but mainly in the liver, in agreement with their poor stability, large size distribution, and poor surface functionalization that favoured size exclusion trapping in lungs and their fast recognition by the reticuloendothelial system [143].

More promising, MSNPs with geometrical size in the range 100–150 nm, and displaying regular arrays of pores, were also radiolabelled to study their specific accumulation in solid tumours via the EPR effect, following a pre-targeting strategy [144]. The surface of these nanoparticles was covered by PEG_12_ and PEG_24_ chains to impart stability and stealth, and a sufficient blood circulation time. The end of the PEG_24_ chain was equipped with a dibenzocyclooctyne (DBCO) group, for coupling in vivo with a [^18^F]-labelled alkylazide. A persistently strong tumour signal in the mice was observed in PET images when they were given DBCO-PEG-MSNs 24 h before injecting the [^18^F]-labelled molecular probe. In comparison, the non-pre-targeted mouse that received only the molecular probe had considerably less tumour uptake. This study clearly demonstrated the interest in MSNPs as a pre-targeting tool for the detection of solid tumours with radioelements of short half-life. It also suggests that this method could enable real-time monitoring of MSNPs during drug delivery.

Cylindrical silica nanoparticles doped with Gd^3+^ ions have been functionalized with [^18^F]-FDG to provide a multimodal imaging platform. The surface of these particles was conjugated with L-glutamine to improve their solubility and stability, resulting in a ζ potential of −29.3 mV, an average size of ca 130 nm, and a molecular weight of ca 3.3 kDa. These nanoparticles showed a high uptake in primary breast cancer and brain tumours that could be imaged by both PET and MRI [145].

Other silica nanoparticles with no specific surface engineering were also studied as control nanomaterials for the development of [^18^F]-radiolabelling methods of metal oxide and hydroxide nanoparticles [58,92].

## 3. Methods for Generating Radiofluorinated Nanoparticles

### 3.1. Introduction to the Radiofluorination Methods

**Figure 16:**
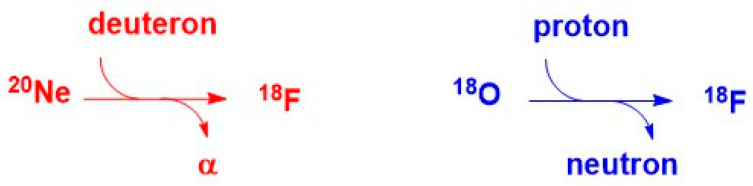
Two modes of ^18^F production: electrophilic fluorine (**left**) vs. nucleophilic fluoride (**right**).

As ^18^F cannot be found in nature, it has to be produced. This occurs using a cyclotron as particle accelerator (Figure 16). This generates high energy particles able to interact with the nucleus of atoms, promoting the nuclear reaction. For ^18^F production, two main approaches are commonly performed. The first one is based on the ^20^Ne(d,α)^18^F reaction. The deuteron bombardment on a target filled with pressurized ^20^Ne gas mixed with a few µmol of F_2_ gas allows (after α emission) for the formation of a [^18^F]-fluorine atom, which provides ^18^F-^19^F diatomic molecules after atomic recombination. This source of electrophilic fluorine thus suffers isotopic dilution and low molar radioactivity.

A second method is based on the ^18^O(p,n)^18^F reaction. In this case, the proton bombardment of a target filled with [^18^O]-water provides (after neutron emission) [^18^F]-fluoride. This nucleophilic source of [^18^F]-fluorine is the obviously most stable chemical form of fluorine, commonly found in nature. It also exhibits significantly higher (at least three orders of magnitude higher) molar radioactivity. Thus [^18^F]-fluoride appears as the generally preferred source of radioactive, positron emitting, fluorine.

#### 3.1.1. Generation of [^18^F]-Fluoride from [^18^O]-H_2_O

As mentioned above, production of ^18^F, under its fluoride form, is carried out in a cyclotron by bombardment of high energy protons onto a target containing ^18^O isotopes. Although [^18^O]-H_2_O is most commonly used, any material containing ^18^O might be involved in the nuclear transmutation. Still, it has to be pointed out that the interaction of the high energy particle beam with the target material involves a significant increase in temperature of the latter (above 1000 °C) [146], thus ruling out the direct involvement of sophisticated [^18^O]-labelled organic molecules. This explains why the ^18^O isotope is introduced via a water molecule. The bombardment thus provides fluoride in [^18^O]-water. Although this [^18^F]-fluoride could potentially be used directly, it should be noted that the process also generates cationic radioisotopes. Those are generally separated from the fluoride by way of an anion exchange column (Quaternary MethylAmine resin or QMA). The nature of these cationic pollutants obviously depends on the nature of the target material and can be characterized by gamma ray spectrum of the QMA [147]. [^18^F]-fluoride is then recovered from the QMA by elution with an aqueous solution, in which the composition sets the pH.

#### 3.1.2. Preparation of Fluoride for Nucleophilic Reaction in Organic Solvents

To promote a nucleophilic reaction of [^18^F]-fluoride on an electrophilic organic partner, a careful preparation of the former is required. Indeed, nucleophilic reaction on organic substrates are hampered by the high hydration energy of fluoride. Moreover, hydrated fluoride, despite its negative charge, fully lacks any nucleophilicity in organic solvents. Thus, an appropriate preparation of the fluoride is not limited to the elimination of the cationic pollutants arising from the target but also requires the complete removal of water. This is usually achieved after elution of the QMA with a moderately basic mixture (CO_3_^2−^ or HCO_3_^−^) of water and acetonitrile containing either a tetraalkylammonium cation or a mixture of cryptand (classically Kryptofix^®^ 222) and potassium salt. Once the radioactive fluoride has been eluted from the resin, an iteration of azeotropic distillations of the mixture is performed by heating under a moderate vacuum or a flow of inert gas with iterative addition of acetonitrile, providing the reactive dehydrated [^18^F]-fluoride, in which the solubility in the organic phase is enhanced by the cation (and its cryptand). Unless noted in the following description of the methods for radiolabelling nano-objects, this process is a common part of all the radiofluorination methods.

### 3.2. Direct Introduction of Fluoride through Interaction with an Internal Element of the Nanoparticle

#### 3.2.1. Bombardment of a Nanoparticle Containing ^18^O

As mentioned previously, high energy proton bombardment on a target is deleterious for the structure of organic materials. Yet it has been shown that it is compatible with some inorganic nano-objects such as naked alumina nanoparticles [90]. Indeed, direct bombardment of [^18^O]-Al_2_O_3_ allowed the formation of radiofluorinated alumina nanoparticles. Interestingly, as high energy proton bombardment promotes both ^16^O(p, α)^13^N and ^18^O(p,n)^18^F reactions, both decay of ^13^N and ^18^F were observed. However, when carried out in ^18^O-enriched water with [^18^O]Al_2_O_3_, the radioactive decay indicates the dominant presence of ^18^F (71%). Furthermore, after 1 h, a complete decay of ^13^N leaves ^18^F as the sole radionuclide. TEM, XRD, and DLS analyses showed no evidence of a modification of the general morphology and structure of the nanoparticles. The same approach has also been performed on ^18^O-enriched titanium dioxide nanoparticles allowing the formation of ^18^F containing TiO_2_ nanoparticles [85]. High resolution gamma ray spectroscopy confirmed the generation of metal isotopes such as ^48^V and ^47^V and ^44g^Sc arising from interaction of low-incident protons with the stable isotopes of Ti (namely ^47^Ti and ^48^Ti, of 7 and 74% natural abundance, respectively). A detailed analysis of all positron isotope emitters was also carried out allowing a precise determination of the relative abundance of all radionuclides. Longer-lived radionuclides are assumed to allow combination of in vivo and ex vivo studies (imaging and dissection/γ counting).

#### 3.2.2. Direct Reaction of [^18^F]-Fluoride with Elements of the Nanoparticle

A first convenient approach to introduce [^18^F]-fluoride within a nanoparticle shall take advantage of the well-known affinity of numerous metal and main group elements for fluoride. Although simple in nature, this approach might be considered potentially deleterious for the physico-chemical properties of the nanoparticle, in which the integrity can hardly be assessed by conventional methods for radiation hazard reasons. Thus, characterizations shall either take advantage of analytical techniques available in radiochemical facilities or be carried out after radioactive decay. The molar amount of radioactive fluoride introduced is very low owing to its high specific activity, and the effect on the physico-chemical properties of the nanoparticle is expected to remain limited. Representative examples of this approach are provided below.

Direct radiofluorination of hydroxyapatite (HA), either untreated thermally, calcinated, or hydrothermally treated, and aluminium hydroxide nanoparticles with ^18^F has been performed by incubation of the aqueous [^18^F]-fluoride solution obtained from the cyclotron at room temperature [92]. The radiolabelling yield did not depend significantly on the preparation method but was impaired by the presence of alendronate or carboxylate-PEG-OMe at the surface. These ligands are indeed supposed to coordinate to surface calcium ions, forming calcium phosphonate or calcium carboxylate bonds, respectively, leaving less calcium ions available for labelling. After a detailed exploration of the reaction conditions, optimized conditions were found in which radiofluorination was shown to occur and reach a plateau after 5 min, exhibiting a high labelling efficiency even at concentrations of nanoparticle below 1 mg/mL. HA-based radiolabelled nanoparticles were also shown to be stable in human serum, whereas the aluminium hydroxide ones were prone to release the radioactivity. In a closely related approach from a practical point of view, radiofluorination of a range of rare earth-based up-converting nanoparticles (UCNPs) has been performed by incubation with 100 µL of an aqueous solution of [^18^F]-fluoride at room temperature with sonication for 1 min [58,70]. Radiochemical conversion rates ranging from 50 to 90% have been reported, establishing the efficiency of the process. Interestingly, silica encapsulated particles, as well as SiO_2_ and ZnO nanoparticles, were poorly to unreactive under the same conditions, indicating a non-expected screening effect of silica. This lack of reactivity in the radiofluorination conditions used indirectly confirmed the rare earth fluoride bonding in the UCNPs. Stability was assessed by incubation in PBS for 2 h, showing a variable dissociation of radioactive fluoride depending on the nature of the anion associated with the rare earth cation (fluoride and hydroxide showing the lowest leakage of [^18^F]-fluoride). This method was more recently applied to UCNPs capped with etidronic acid, alendronic acid, and nitrilotrimethylphosphonic acid, where the former exhibited a low propensity to radiofluorination (RCY < 10%) while the two others were equally efficiently radiolabelled (43%) [66]. The affinity of rare earth for fluoride was also the basis for the radiolabelling of YF_3_ nanoparticles loaded with a range of surface ligands such as citric acid, folic acid, doxorubicin, or PEG linkage [62]. Again, a simple incubation (30 min) at room temperature provided excellent radiochemical yields ranging from 84 to 96%, exhibiting an excellent kinetic profile of the reaction. Interestingly, purified nanoparticles incubated for 30 min in human or mouse serum released only up to 6% of their radioactivity, exhibiting no further release upon prolonged incubation, and thus establishing the good stability of these constructs. This illustrates again a fast establishment of the equilibrium, but leaves open the question of further release of fluoride after injection in a living body. Related core–shell colloidal nanoparticles made of iron oxide surrounded by a NaYbF_4_ or NaErF_4_ shell have been radiolabelled by a simple isotope exchange occurring on incubation with aqueous fluoride at room temperature, under similar reaction conditions, albeit with lower efficiencies (38%) [84]. The stability of the radiolabelling was confirmed by prolonged incubation in human serum, centrifugation, and activity counting of the supernatant and the nanoparticles filtered. Over 85% of the radioactivity was shown to remain on the nanoparticle. An effect of the size of the nanoparticles was somehow observed. Indeed, Sub-5nm KGdF_4_:Eu^3+^ nanoparticles coated with polyacrylic acid have been shown to undergo direct radiofluorination in aqueous solution via a simple 10 min incubation with [^18^F]-KF and removal of the unreacted fluoride by centrifugation [59]. The radiolabelling yield has been estimated to be around 50%, which is somehow lower than for larger (around 25 nm, hence exhibiting a higher surface) rare earth nanoparticles for which RCY reached 80%.

As previously illustrated, the impact of the coating on the radiofluorination of nanoparticles is an obvious matter that has been also illustrated by involving aluminium. Indeed, Fe_3_O_4_ nanoparticles with a layer of Al(OH)_3_ exhibiting a hydrodynamic diameter of 573 nm and a ζ potential of +18 mV have been efficiently radiofluorinated through an incubation with a saline [^18^F]-fluoride solution for 10 min at room temperature [83].

Despite silica coating being shown to be somehow detrimental for radiolabelling UCNPs, silicon is well-known for its affinity to fluoride; thus, silicon-based nanoparticles are also illustrated in this section. Porous silicon nanoparticles either thermally hydrocarbonized (THCPSi), thermally oxidized (TOPSi), and thermally carbonized (TCPSi) have been directly radiofluorinated (generation of a Si–F bond) by reaction with pre-dried [^18^F]-fluoride, Kryptofix^®^ 222, and potassium fluoride in various solvents at various temperatures [135]. This study indicates a moderate interest of adding acetic acid to perform the reaction, although it was previously reported as advantageous for the radiofluorination of Si–H-functionalized surfaces. A reaction carried out in DMF at 120 °C gave an RCY of up to 81% with 1 h of synthesis time. THCPSi nanoparticles similarly radiolabelled by incubation in the presence of acetic acid could be then further coated with functional proteins to modulate their biodistribution. The overall RCY after coating was lower, reaching only 40%, but coating allowed a formulation in saline aqueous solutions [137]. Coating of THCPSi with solid lipids allowed the formation of a solid lipid nanocomposite containing [^18^F]-THCPSi ([^18^F]-THCPSi-SLNCs) in up to 30% RCY [17]. Unsurprisingly, the extra coating occurred with a decrease in the molar radioactivity of the particles.

Finally, the direct radiolabelling of organic nano-objects has also been performed as illustrated for the specific case of nano-graphene oxide, more specifically nGO-PEG derivatives [131]. With this substrate, the radiofluorination was shown to occur upon heating for 1 h in a TFA aqueous solution (pH 5.5) at 80 °C. The reaction was reported as highly efficient (RCY over 96%). FT-IR investigation of [^19^F]-fluorinated nGO indicates a high content of fluoride, which is statistically unlikely for ^18^F. However, it is worth considering that the radiofluorinated nGO-PEG exhibits a high stability in aqueous solution.

#### 3.2.3. Inclusion of a Radiolabelled Fragment during the Preparation of the Nanoparticle

Generation of the nanoparticle in the presence of fluoride, or of a previously fluorinated constituent, is another approach to introduce a radioactive fluoride through a direct interaction with the constituents of the nanoparticle. Relevant illustrations of a such an approach are provided in this section.

A first example where the fluoride anion is directly engaged is related to the synthesis of up-converting nanoparticles labelled with ^18^F by co-precipitation under vigorous stirring of NaNO_3_, Bi(NO_3_)_3_, Yb(NO_3_)_3_, or Tb(NO_3_)_3_ in the presence of NH_4_F and [^18^F]-NaF for 1 min at room temperature in air [72]. The stability of the radiolabelled nanoparticles was evaluated in fetal bovine serum for up to 4 h showing by rTLC no release of free [^18^F]-fluoride.

**Figure 17:**
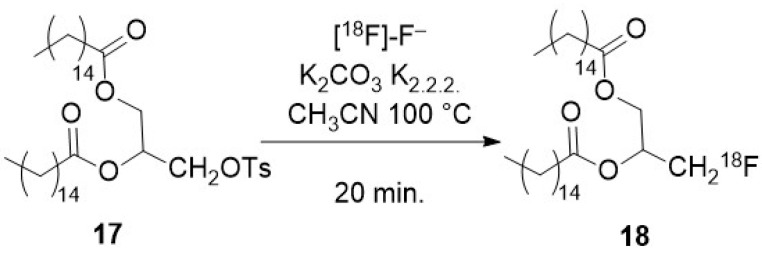
Synthesis of [^18^F]-fluorodipalmitin **18** ([^18^F]-FDP).

A related strategy, albeit involving a covalently bonded fluorine atom, has been applied for the formation of other radiolabelled nano-objects, namely [^18^F]-labelled liposomes. These are based on the radiosynthesis of fluorinated lipids that are then mixed with amphiphilic compounds. As such, [^18^F]-fluorodipalmitin **17** ([^18^F]-FDP) has been synthesized from its corresponding tosylate **17** (Figure 17), from which the radiolabelled liposomes where obtained upon addition of DPPC, DSPE-PEG2000, and cholesterol, followed by evaporation and sonication for 10 min at 50 °C after resuspension in a PBS solution, and finally filtration on polycarbonate membranes [30].

In a similar approach, [^18^F]-FDP **18** has also been engaged in the formation of liposomes featuring arginine-rich peptides functionalized with lipophilic tails [35]. With these constituents, liposome formation occurred upon addition of a suspension of the mixture above with [^18^F]-FDP **18**, sonication at 60 °C for 1 min, filtration through polycarbonate membrane and purification on a sephadex column. Of course, other radiofluorinated lipophilic derivatives have been engaged in related approaches.

**Figure 18:**
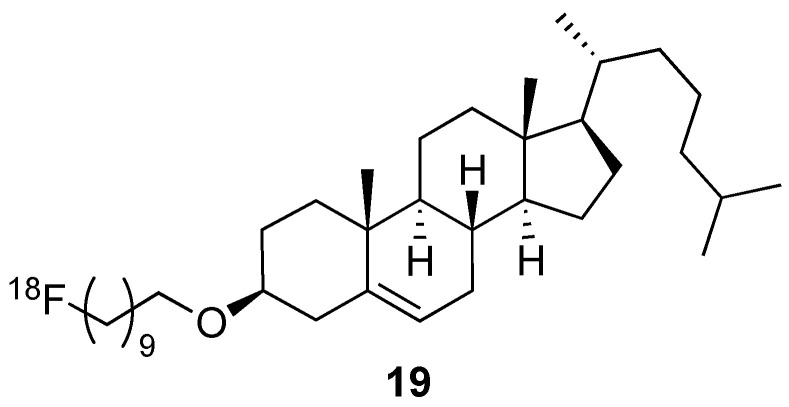
Structure of 10-cholesteryloxy-1-[^18^F]-fluorodecane **19**.

Cholesterol-based liposomes have been obtained by mixing 10-cholesteryloxy-1-[^18^F]fluorodecane **19** with DSPC, DSPE/PEG2000, and cholesterol in chloroform (Figure 18), followed by evaporation and stirring for 30 min at 60 °C in HEPES buffer at pH 7.4 [37].

The radiofluorinated probe has been accessed by a standard S_N_2 reaction of the corresponding mesylate in DMSO for 10 min at 165 °C, and a two-step Sep-Pak (C_18_ and Silica) with a 1% radiochemical yield.

**Figure 19:**
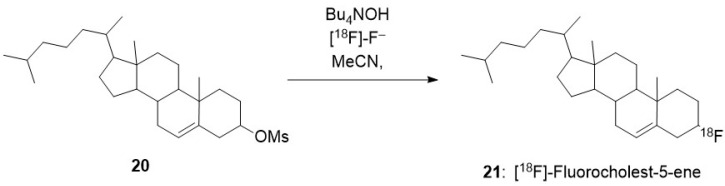
Synthesis of [^18^F]-fluorocholest-5-ene **21**.

**Figure 20:**
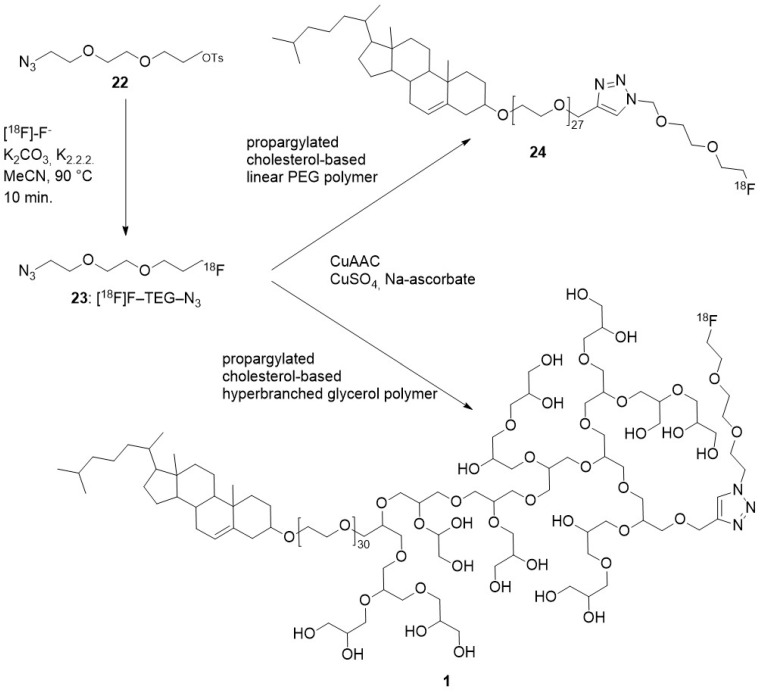
Synthesis and CuAAC reactions of [^18^F]-TEG-N_3_ **23**.

Different approaches of radiolabelling have been followed to access a range of radiofluorinated liposomes constituted of PEG or hyperbranched polyglycerol (hbPG) polymers and cholesterol [18]. The first one involved the preparation of [^18^F]-fluorocholest-5-ene **21** by nucleophilic displacement of the methylsulfonate derivative of cholesterol **20** (Figure 19). The second and the third were based on a CuAAC reaction of alkyne-functionalized PEG- and hbPG-substituted cholesterol with [^18^F]-TEG–N_3_ **23** leading, respectively, to **24** and **1**. Liposomes were then formulated with dioleoyl-*sn*-glycero-3-phosphatidylcholine (DOPC), cholesterol, and the radiolabelled copolymer with a lipid to cholesterol ratio of 60:40 (Figure 20).

Similar radiolabelling has been performed using [^18^F]-TEG–N_3_ **23** on linear and hyperbranched hexadecylbased polymers. The stability of the liposome was assessed, right after decay and after one month, by measuring the hydrodynamic radius of the liposomes. This was achieved at temperatures ranging from 20 to 50 °C by 5 °C increments, showing no evolution of the measured radii [23].

**Figure 21:**
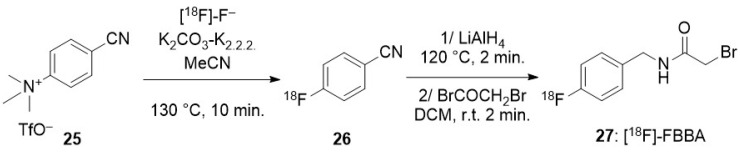
Synthesis of [^18^F]-FBBA **27**.

The use of general purpose radiolabelling prosthetic groups has also been investigated. As such, [^18^F]-Fluorobenzyl-2-bromoacetamide **27** ([^18^F]-FBBA) has been engaged in the radiolabelling of Paclitaxel-loaded polyester nanoparticles [45]. FBBA was obtained by fluorination of trimethylammoniumbenzonitrile **25** through a S_N_Ar displacement followed by LAH reduction of **26** and acylation with bromoacetyl bromide (Figure 21).

**Figure 22:**
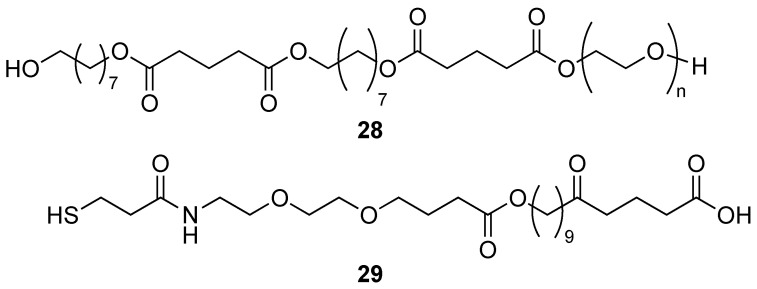
Block-copolymers involved in the formation of Paclitaxel-loaded and [^18^F]-FBBA-labelled nanoparticles.

Formation of the polymer-based nanoparticle was achieved through the following three distinct strategies using different block-copolymers (Figure 22), which were shown to impact the distribution, metabolism, and elimination:

(i) using a mixture of radiolabelled block-copolymer **28** and one copolymer linked to Paclitaxel; (ii) using a mixture of block-copolymer **28**, radiolabelled block-copolymer **29,** and one copolymer linked to Paclitaxel; and (iii) a mixture of copolymer **28**, FBBA, and one copolymer linked to Paclitaxel. Somehow expectedly, the radioactivity was progressively released from the third nano-object, most probably through water diffusion in the polymer core matrix.

**Figure 23:**
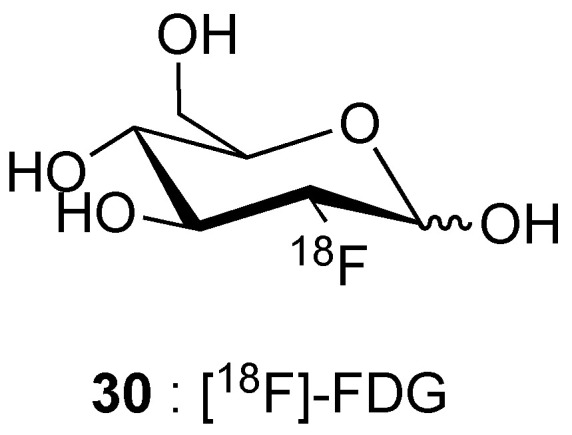
Molecular structure of [^18^F]-FDG **30**.

A less-common approach towards radiolabelled gold nanoparticles has been performed through a double derivatization of [^18^F]-FDG **34** (Figure 23), pre- and post-radiolabelling [12].

**Figure 24:**
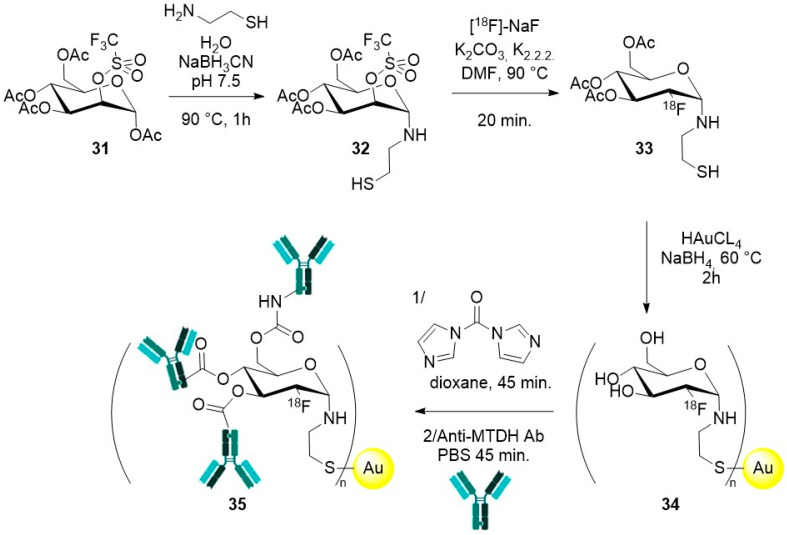
[^18^F]-FDG-based generation of radiolabelled and vectorized gold nanoparticles **35**.

As illustrated in Figure 24, the process is initiated by the reaction of cysteamine with the peracetylated mannose triflate **31**, precursor of the [^18^F]-FDG synthesis, followed by radiofluorination of **32** in DMF to provide **33**. The gold nanoparticles **34** are generated from HAuCL_4_ in the presence of NaBH_4_ and **33**, which is thus bound to gold through its thiol terminus. The acetates are cleaved under these reductive conditions. Upon reaction with carbonyldiimidazole (CDI), the released alcohols offer handles to conjugate antibodies such as in **35** for which anti-MTDH are favouring a vectorization to MCF7 breast cancer cells. Although this use of the [^18^F]-FDG scaffold to link the nanoparticle to an antibody appears highly original, the overall time of synthesis (almost 5 h) questions the global efficiency of the process from a radiochemical perspective.

A simplified but similar strategy has been applied to the synthesis of magnetic iron oxide nanoparticles [148]. Indeed, addition of iron (III) chloride and sodium borohydride, at pH 9 and 60 °C, to a solution of the peracetylated [^18^F]-FDG-cysteamine derivative **37** allowed the isolation, after 2 h, of black magnetite (Fe_3_O_4_) nanoparticles, which could be separated using a magnet.

**Figure 25:**
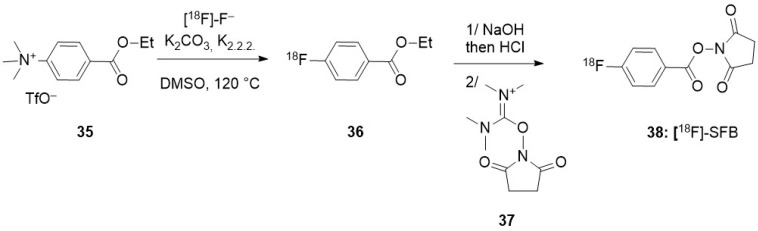
Synthesis of [^18^F]-SFB **38**.

[^18^F]-succinimidylfluorobenzoate ([^18^F]-SFB) **38**, another very common fluoroaromatic prosthetic group, has been used to radiolabel a wide range of nanoparticles. [^18^F]-SFB **38** is generally synthesized from the ammonium **35** after saponification of **36** and further activation using TSTU **37** as illustrated in Figure 25 [149].

Although [^18^F]-SFB **38** has been extensively used for couplings at the surface of preformed nanoparticles (see Section 3.3.2), it was also involved in one example of in vivo formation of radiolabelled nanoparticles. First, **38** was used to radiolabel the amino group of the lysine of an Ac-RVRRC(StBu)K peptide designed for detection of tumour cells exhibiting a furin overexpression. This lysine also featured a 2-cyanobenzothiazole moiety [19]. After injection, in vivo reduction in the disulfide (by GSH inside the cell) was followed by furin cleavage of the cell-penetrating peptide RVRR. This promoted the formation of a dimer via the condensation of the aminothiol of the cysteine terminus with the cyanobenzothiazole moiety of the lysine. Co-injection of the [^19^F]-analog favoured the formation of the radiolabelled dimers. These dimers self-assemble into nanoparticles inside the cells and, as they lack the cell membrane translocating ability of the RVRR fragment, they concentrate the radioactivity inside the tumour cell. Extension of this work has been carried out using the kit-like radiofluorination developed by the Perrin group [150], allowing a simple radiofluorination process based on an isotope exchange on a zwitterionic trifluoroborate (so called AMBF_3_) [151].

### 3.3. Introduction of Fluoride by Modification of the Surface of the Nanoparticle

These various methods consist in preforming the nanoparticles, eventually preparing their surface for the radiolabelling, and then introducing the radioactive fluorine via an organic fragment. The differences can arise from the functionalization of the outer layer of the nanoparticles and from its ability to interact with the radiolabelled probe either through supramolecular-type interactions (including solid-phase transition methods for liposomes), as it will be first exemplified, or through covalent binding. In the latter case, we will first illustrate the common approach that relies on the radiofluorination of the nanoparticle prior to its injection, then we will discuss a more recent process based on bioorthogonal reactions that permits the in vivo labelling of the nanoparticles after their injection, biodistribution, and accumulation in the region of interest.

#### 3.3.1. Direct Introduction of a Radiofluorinated Adduct on the Nanoparticle

##### Supramolecular Interaction

Some specific nanoparticles allow the integration of one radiolabelled constituent after their first generation. This is especially the case of liposomes and of some polymer-based nanoparticles. This differs practically from the “one-pot” generation and radiolabelling of similar nanoparticles described in the previous section by the possibility to isolate and fully characterize the nanoparticles before introduction of the radiofluorinated block. Yet for this post-synthetic approach, one can question the effect of the introduction of another constituent on the nature and properties of the nanoparticles, which might only be assessed after radioactive decay, limiting the exact characterization of the object used for in vivo exploration.

**Figure 26:**
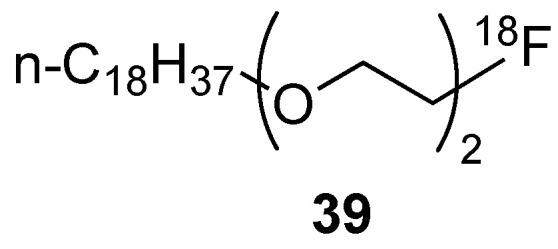
Amphiphilic derivative **39** for solid-phase transition radiofluorination of liposomes.

The radiolabelling of liposomes by solid-phase transition methods has been established as an efficient approach to introduce radiolabelled probes onto pre-formed liposomes [29]. The proof of concept was first established by a thorough identification of amphiphilic compounds (lipids + PEG) of high incorporation efficiency. The obtained liposomes were then incubated at 65 °C for 15 min with few [^18^F]-fluorinated amphiphilic compounds. This process, including repeated vortex mixing, allowed the generation of radiofluorinated liposomes with efficiencies ranging from 60 to 90%. Stability in foetal bovine serum proved highly dependent on the nature of the radiolabelled adduct with **39** (Figure 26) being the most appropriate, both in terms of incorporation efficiency and stability, and far superior to the analogues featuring aromatic groups.

Amphiphilic derivative **39** is obtained by a standard S_N_2-type procedure from its corresponding tosylate. This approach has been applied in, e.g., liposome-encapsulating hemoglobin (LEH) [31]. These have been labelled by incubation of LEH with 1-[^18^F]fluoro-3,6-dioxatetracosane **40** with a labelling efficiency of 46 ± 5% (indicating that half of the radioactivity remained in the supernatant upon incubation). However, the efficiency of the radiolabelling of 3,6-dioxatetracosane tosylate (i.e., prior to the association of the radiofluorinated probe **39** to LEH) should still be considered to evaluate the overall radiolabelling efficiency.

The same authors have reported a similar approach to radiolabel another kind of liposome made of disterearoylphosphatidylcholine, cholesterol, and distearoylphosphatidyletanolaminePEG, with a PEG of 2000 g/mol with no further details than previously reported [33].

**Figure 27:**
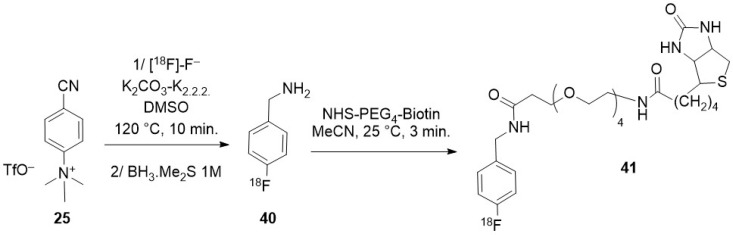
Synthesis of the [^18^F]-radiolabelled biotin derivative **41**.

Alternatively, the use of the biotin–avidin naturally occurring supramolecular bioconjugating process has also been investigated in order to perform the radiofluorination of PLGA nanoparticles [44]. To achieve this coupling, PLGA nanoparticles were functionalized with avidin. On the other side, a biotin radioconjugate **41** was synthesized using a fluorobenzylamine **40** obtained by BH_3_•SMe_2_ reduction of the corresponding 4-fluorobenzonitrile (Figure 27). The radiofluorination of the latter was previously described for [^18^F]-FBBA **27** (Figure 21) to occur by S_N_*Ar* displacement of the trimethylammoniumbenzonitrile **25**.

Confirmation of the stability of the radiolabelling was performed by sonication of the radiolabelled nanoparticles for 30 min and subsequent centrifugation: no remaining radioactivity was detected in the supernatant. Yet one could question the solubility of the fluorinated adduct NPB4 in the aqueous buffer, which could be trapped in the polymer nanoparticle, or on its surface, but could be transferred to lipophilic biomolecules in vivo, thus providing an off-target image.

##### Covalent Labelling via SN2-Type Reaction

In a related fashion, micelles made of a block-copolymer featuring mesylate leaving groups on pendant alkyl chains have been submitted to a direct radiofluorination. This has been achieved with [^18^F]-fluoride under standard S_N_2-type reaction of mesylates introduced at termini of PEG chains in the presence of BHT with a radiochemical conversion of 61% [50]. These radiofluorinated polymers, obtained from ROMP polymerization of norbornene-imides and cross-linked with cinnamoyl moieties, were shown to undergo a full displacement of the mesylate termini by further addition of [^19^F]-fluoride to avoid undesired reactivity of the mesylates in vivo.

#### 3.3.2. Coupling of a Radiofluorinated Prosthetic Group at the Surface of a Nanoparticle

An alternative approach to perform the radiofluorination of nanoparticles consists in incorporating the prosthetic group to a pendant chemical function displayed at the surface of the nanoparticle.

##### *Ex Vivo Radiolabelling* 

C-F-Based Derived Prosthetic Groups

**Figure 28:**
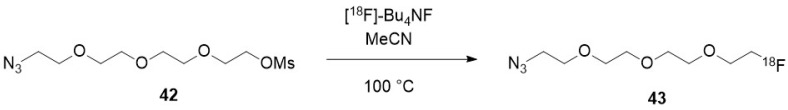
Synthesis of [^18^F]-fluoroethyl-diethyleneglycol-ethylazide **43**.

A short [^18^F]-fluoroethyl-diethyleneglycol-ethylazide **43** has been involved in the CuAAC coupling with the alkyne termini of zinc oxide nanoparticles (Figure 28) [75,88]. The radiofluorinated adduct was classically obtained from **42** by displacement of a mesylate in acetonitrile at 100 °C in the presence of 37 MBq (1 mCi) of [^18^F]-tetrabutylammonium fluoride. It was purified by a short path silica gel chromatography. The stability of the obtained nanoparticles has been established by incubation both in saline and HCl solution, which led to no radioactivity release for up to 7 h, as identified by radio-TLC.

**Figure 29:**
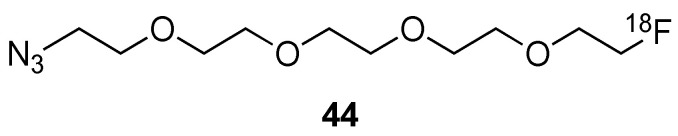
Elongated [^18^F]-fluoroethyl-PEG-azide **44**.

The related [^18^F]-fluoroethyl-triethyleneglycol-ethylazide **44** (Figure 29) has been engaged after common radiofluorination in the CuAAC (in the presence of copper sulfate and sodium ascorbate) to label dextran-coated iron oxide nanoparticles featuring alkyne termini [76].

This same approach had been previously explored with success for the generation of dextran-coated iron oxide nanoparticles [74].

**Figure 30:**
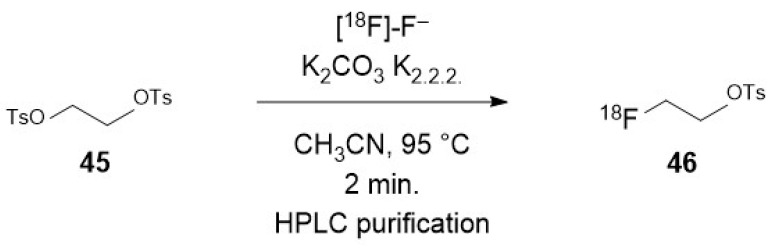
Synthesis of [^18^F]-fluoroethyltosylate **46**.

A shorter prosthetic group such as [^18^F]-fluoroethyltosylate **46** has also been engaged in the radiolabelling of low and high molecular weight methacrylate-based polymers featuring derivatizations with folic acid and tyramine residues [40]. [^18^F]-Fluoroethyltosylate **46**, generated under conventional radiofluorination methods from **45** (Figure 30), was reacted with the phenol moiety of tyramine residues in the presence of sodium hydroxide, providing a 5 and 10% radiochemical yield for high and low molecular weight polymers, respectively.

This [^18^F]-fluoroethyltosylate **46** was also engaged in the radiofluorination of polymeric nanoparticles. In this scenario, block-copolymers featuring one phenol fragment are alkylated by **46**. One early example of radiofluorination of a biocompatible N-(2-hydroxypropyl)-methacrylamide (HPMA) polymer has been achieved by modification of the polymer with tyramine [41]. This also allowed the radiofluorination by alkylation of the phenol moiety of the tyramine with **46**, obtained by S_N_2-type reaction between [^18^F]-fluoride and ethylene ditosylate **45** (Figure 30). The obtained polymer particles showed excellent stabilities, minimal metabolism, and an exclusive renal clearance evidenced by their accumulation in kidneys and the bladder. Extension of this work to HPMA polymer particles doped with lauryl methacrylate (random or as block-copolymer) demonstrated a linear variation in tumour uptake and molecular weight of the polymers. Thus, it illustrated the need to individualize polymer-based radiotherapy as tumour uptake is strongly related with the tumour model [42].

**Figure 31:**
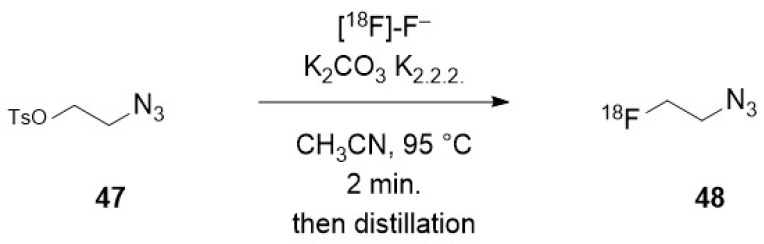
Synthesis of [^18^F]-fluoroethylazide **48**.

Another short prosthetic group, namely [^18^F]-fluoroethylazide **48**, has been obtained from its corresponding tosylate **47**, using the standard S_N_2-type radiofluorination method (Figure 31) [78].

This radiolabelled fragment was then engaged in a CuAAC reaction (in the presence of copper sulfate and sodium ascorbate) with the alkyne termini of ultrasmall superparamagnetic iron oxide (USPIO) nanoparticles coated with a polyacrylic polymer, then with polydopamine, and also featuring a cyclic RGDfK terminus for vectorization. The key alkyne functionalization of the USPIO was achieved by incubation with an amino–PEG–alkyne, in which the amine reacts via a Michael reaction with the quinone moiety of the polydopamine coating.

**Figure 32:**
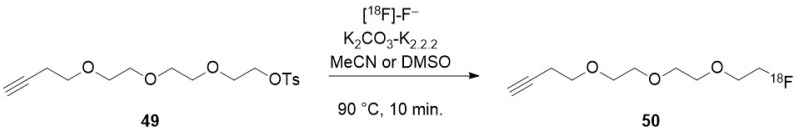
Synthesis of propargylated [^18^F]-fluoroethoxydiethylene glycol **50**.

Reversing the partners of the CuAAC reaction, organic nanoparticles composed of 40% polyglucose and 60% L-lysine functionalized with azide termini have been radiolabelled with a propargylated [^18^F]-fluoroethoxydiethylene glycol **50** [152]. The latter was classically prepared from its corresponding tosylate **49** and purified by preparative HPLC to provide 56% RCY of the radiolabelled alkyne intermediate (Figure 32), which after CuAAC coupling provided the organic nanoparticles in 38% decay-corrected RCY in almost 2 h. When performed from 37 GBq of [^18^F]-fluoride in a hot cell, the method gave access to the radiofluorinated nanoparticles in lower yield (13%), probably related to the lower yield achieved for the alkyne intermediate, which was then carried out in DMSO in 23% RCY.

A related method has been employed to perform the radiofluorination of iron oxide nanoparticles featuring surface functionalization with catechols bearing either a folic acid or an azido acetyl group [79]. In this case, two paths were investigated. One was based on a late radiolabelling strategy, with a coupling occurring between the azide and 5-tosyl-pentyne, followed by nucleophilic displacement of the tosyl by [^18^F]-fluoride under conventional conditions. The other, in a two-step process, required the preliminary formation of 5-[^18^F]-fluoropentyne from the corresponding 5-tosyl-pentyne, followed by the CuAAC to the azide carried by the nanoparticle catechol ligand. The former proved more efficient, which could be related to the known low boiling point of 5-[^18^F]-fluoropentyne [153].

**Figure 33:**
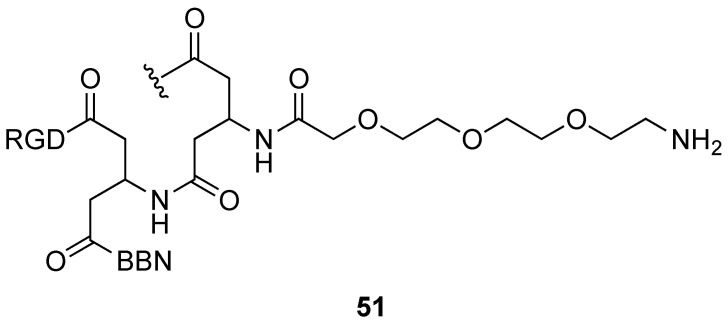
Structure of tertiary linker for vectorization and radiofluorination of QDs.

In order to take advantage of the facile formation of amide bonds, NFP ([^18^F]-fluoropropionate *p*-nitrophenol), an activated [^18^F]-fluorinated ester, has been involved in the overall labelling of QDs [97]. For that purpose, amine-modified quantum dots have been submitted to multiplex functionalization through a tertiary linker **51**, thus allowing the conjugation of bombesin (to target Gastrin-releasing peptide receptor) and RGD (to target integrin α_V_β_3_) (Figure 33), as discussed in Section 2.2.3, and a further reaction with [^18^F]-NFP **54**.

**Figure 34:**
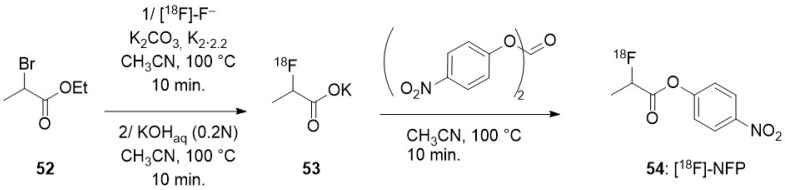
Synthesis of [^18^F]-NFP **54**.

[^18^F]-NFP **54** has been obtained through a three-step route starting with standard fluorination (Figure 34), followed by saponification and ester activation with bis-4-nitrophenylcarbonate at 100 °C for 10 min.

**Figure 35:**
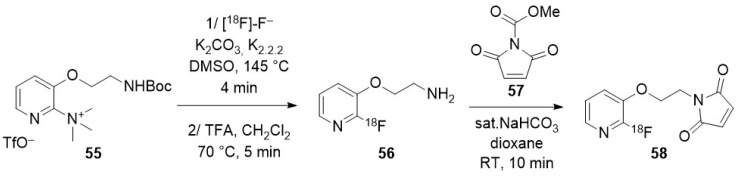
Synthesis of [^18^F]-FPyMe **58**.

Prosthetic groups featuring [^18^F]-(het)aryl fluorides are also frequently reported. Among the examples available, (2-fluoropyrid-3-yloxy)propylmaleimide, FPyME **59**, in which three-step synthesis was previously described for protein radiofluorination (Figure 35) [154], has been engaged in the labelling of thiol termini containing QDs. These thiol termini were obtained by converting the amino groups present in the PEG phospholipid micelles, which encapsulated the QDs [16]. Fluorination was carried out on **56**, followed by Boc deprotection yielding **57**. Introduction of the imide moiety was performed by exchange with **58** providing, after purification by HPLC and evaporation of the solvents, [^18^F]-FPyME **59**.

**Figure 36:**
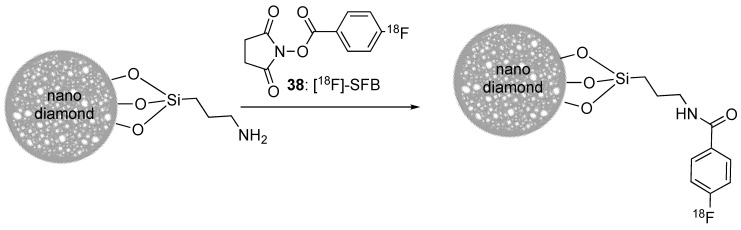
[^18^F]-radiolabelling of nanodiamonds.

Among the array of prosthetic groups, [^18^F]-SFB **38** has been the most employed one. A first example reported the radiofluorination of nanodiamonds. These nanodiamonds were first treated by Fenton oxidation and surface-modified with aminopropyltriethoxysilane. Coupling of the surface amino groups with [^18^F]-SFB **38** afforded the radiolabelled nanoparticles (Figure 36) [130].

[^18^F]-SFB **38** was also found to demonstrate successful application for the radiolabelling of gold nanoparticles functionalized with cysteine–lysine (CK) dipeptide and a CLPFFD peptide for brain penetration [120]. Once classically synthesized and purified by semipreparative HPLC, [^18^F]-SFB **38** was dried and resuspended with a DMSO/sodium citrate solution of the gold nanoparticles, with stirring prolonged for 1 h at 50 °C. This yielded the radiolabelled nanoparticles with 0.8% RCY, thus exhibiting a limited efficiency. Their stability was assessed from incubation in plasma followed by dialysis and UV–Vis measurements showing a perfect stability over a 2 h period and a slight decrease over 1 d. This was somehow confirmed using the high sensitivity of the radioactivity detection combined with centrifugation and counting of the supernatant and the solid, which showed a release of 25% of the radioactivity in the supernatant.

[^18^F]-SFB **38** has also been engaged for the radiolabelling of mesoporous silica nanoparticles functionalized with amino groups by way of sonication of colloidal silica nanoparticles with aminopropyltriethoxysilane [143]. The combination of the [^18^F]-SFB **38** probe with the amino groups of these nanoparticles has been achieved by reaction of the two partners in a mixture of DMSO and sodium citrate buffer at pH 11 for 45 min, followed by a dilution with water and three iterative centrifugations providing an overall radiochemical yield of 14%. Similarly, [^18^F]-SFB **38** has also been used as a radiofluorinated probe to label polymeric nanoparticles made of acryloylated BSA coated with 2-methacryloyloxyethyl phosphorylcholine (MPC) and N-(3-amino-propyl) methacrylamide hydrochloride (APM) [155]. The amino residue of the latter was used to both introduce an RGD-A7R peptide and [^18^F]-SFB **38**, at room temperature for 30 min in a borax buffer (pH 8.5). Incubation of the radiolabelled nanoparticles in PBS and calf serum exhibited a lower stability in the latter as identified by thin layer chromatography. The efficient reactivity of [^18^F]-SFB **38** with the amino termini of (aminopropyl)triethoxysilane-functionalized surfaces of nanoparticles has also been applied for the radiofluoration of ceria nanoparticles [89]. The reaction was shown to occur at room temperature within 1 h in a 3:2 mixture of DMSO and phosphate buffer (pH = 7.4). After dilution and centrifugation, the radiofluorinated nanoparticles were obtained with 18% RCY.

[^18^F]-SFB **38** has also been involved in the radiolabelling of a range of polymers featuring polyester grafted with chitosan, polylysine, or phosphatidylserine and calcium phosphate by incubation at 37 °C for 30 min [47]. Although the description of the formation of the polymer nanoparticles remains elusive, their labelling allowed a detailed in vivo investigation of their tumour-targeting ability.

**Figure 37:**
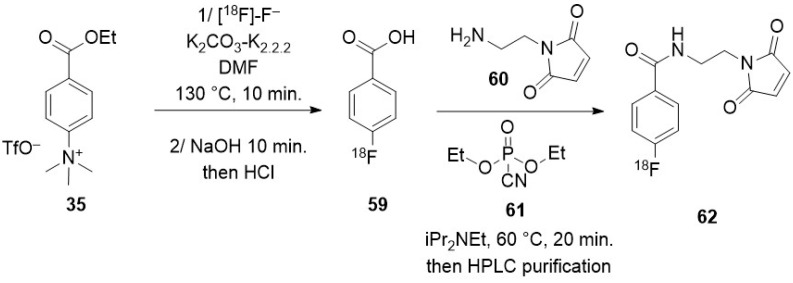
Synthesis of [^18^F]-FBEM **62**.

4-[^18^F]-fluorobenzamido-N-ethylmaleimide ([^18^F]-FBEM **62**) previously described for labelling thiol-containing biomolecules (Figure 37) [156], has been engaged in the radiolabelling of sphingomyelin nanoemulsions functionalized with a thiol group [12]. The synthesis of [^18^F]-FBEM **62** was achieved from **35** through three steps via the intermediate **59**, which reacts with maleimide derivative **60** and the DEPC coupling agent **61**, in an automatized procedure. The synthesis required HPLC purification followed by solvent evaporation as previously performed for biomacromolecules, illustrating a direct parallel between the labelling of biomacromolecules and nano-objects.

Yet the multistep character of this process limits its wide availability and might account for its scarce use for the radiofluorination of nanoparticles.

**Figure 38:**
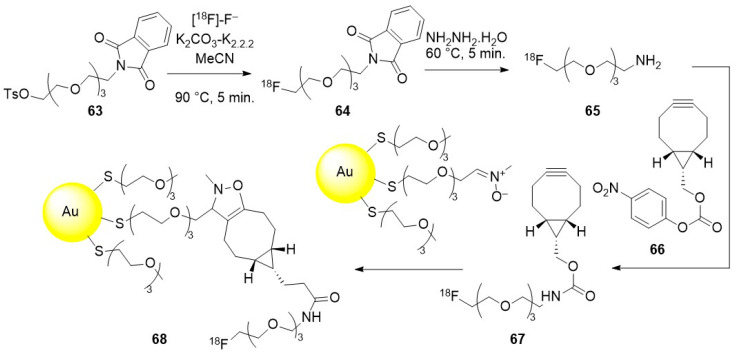
SPANC-based [^18^F]-radiolabelling of gold nanoparticles.

A more innovative approach based on the recent developments of conjugation involving the cycloaddition of strained multiple carbon–carbon bonds is nitrone functionalized gold nanoparticles, which have been radiofluorinated by way of strain-promoted alkyne–nitrone cycloaddition (SPANC) (Figure 38) [124]. The counterpart of the coupling reaction was a fluorinated cyclooctyne **67** obtained in a three-step post-radiofluorination fashion from **63** with an overall 22% RCY (decay corr.). After SPANC coupling, the radiofluorinated gold nanoparticles **68** were obtained with 6% RCY (decay corr.) after purification by size-exclusion chromatography.

Another, even more straightforward route, makes use of the [^18^F]-FDG scaffold. It takes advantage of the high reactivity of the aldehyde of the sugar with hydroxylamino groups to form oxime under aqueous conditions [77]. Thus, for the radiolabelling of iron oxide nanoparticles coated with dextran and functionalized with amino-oxyacetate termini, a simple incubation at 60 °C in PBS for 10 min with routinely prepared and clinically available [^18^F]-FDG allowed the formation of the radiolabelled nanoparticles in 24% radiochemical yield (decay corrected) in 40 min. Although stable for more than 1 h in water, these radiolabelled nanoparticles were found to be rather unstable in plasma as 50% of the radioactivity was released from the nanoparticles in 30 min, which might be due to dextran-coating fragmentation.

Al-F-Derived Prosthetic Groups

Moving away from C–F bond-based prosthetic groups, chelated Al–F derivatives have also been employed for the ex vivo radiofluorination of nanoparticles. One relevant application concerns the radiolabelling of iron oxide nanoparticles coated with oleylamine-branched polyacrylic acid conjugated with a NOTA macrocycle [80]. A simple radiofluorination procedure was performed by incubation of the NOTA-conjugated nanoparticles in a sodium acetate buffer with a pre-heated solution of [^18^F]-NaF and AlCl_3_ at 95 °C for 5 min. Purification was achieved by size-exclusion chromatography. The stability of the construct was assessed on ^19^F isotopologous nanoparticles by measuring via ICP-MS the amount of aluminium released upon incubation in PBS buffer. A high stability was determined as only 5% of the aluminium was released, and this occurred only during the first 2 h.

Si-F-Based Derived Prosthetic Groups

**Figure 39:**
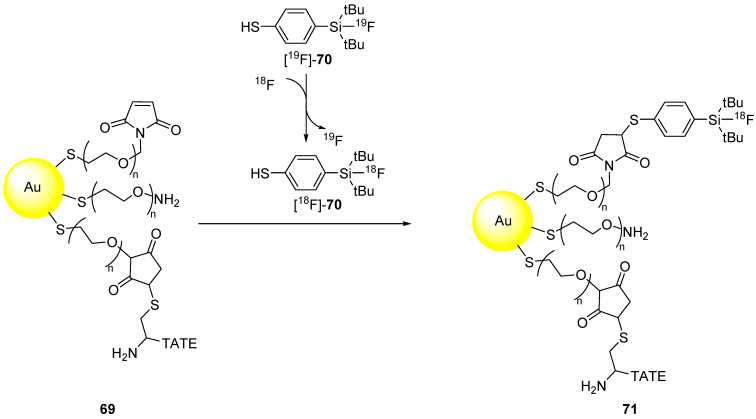
SiFA-based radiofluorination of gold nanoparticles.

The radiolabelling of silicon-based prosthetic groups, previously described for large biomolecule radiofluorination [157], has also been applied for the radiofluorination of gold nanoparticles (Figure 39) [122]. This has been achieved by addition of the thiophenol of SiFA derivative **70** to the maleimide termini on the gold nanoparticle **69** while cysteine-containing TATE was added to other maleimide on the surface to perform the vectorization of the nanoparticle **71**. Fluorination of the SiFA group **70** occurred through a kit-like isotope exchange reaction.

The same SiF- based strategy was more recently applied for the radiofluorination of polymeric core–shell nanoparticles [49]. Here again the isotope exchange method proved efficient as radiofluorinated nanoparticles were obtained in 53 to 72% RCY.

P-F-Based Derived Prosthetic Groups

**Figure 40:**
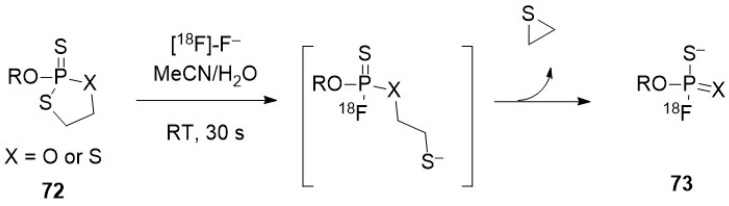
Synthesis of phosphorofluoridodithioate **73**.

Following on isotope exchange-based radiofluorination of phosphorous compounds [158], a nicely innovative approach to radiofluorination of phosphorus-based prosthetic groups has been recently reported and adapted for the radiofluorination of PEGylated Pd nanosheets (Pd PEGs) [125]. The method is based on the nucleophilic ring opening of **72** by [^18^F]-fluoride under non-conventional aqueous conditions (MeCN/H_2_O: 9/1) (Figure 40). This provides, after release of thiirane, the radiofluorinated phosphorofluoridodithioate **73**, in which sulfhydryl groups form stable coordination bonds with Pd-PEGs. However, stability investigations were not thoroughly described.

To conclude, coupling a radiofluorinated probe at the surface of a nanoparticle shares numerous common features with the labelling of biomacromolecules. Conventional C–F-based prosthetic groups, as more innovative approaches have been taking advantage of the fast an efficient labelling of main group elements, have been involved in the radiofluorination of nanoparticles. It clearly and obviously appears that the most straightforward methods to achieve the synthesis of radiolabelled prosthetic groups are the most appropriate, and that multistep approaches shall be difficult to implement in a systematic fashion. Once the radiolabelling technique is chosen, numerous coupling methods can be adapted as soon as their kinetics and chemical efficiencies are high (to avoid long reaction times and achieve high radiochemical yields).

This parallel between biomacromolecules and nanoparticles is similarly observed by the development of highly innovative in vivo labelling techniques as illustrated below.

##### *In Vivo Radiolabelling* 

**Figure 41:**
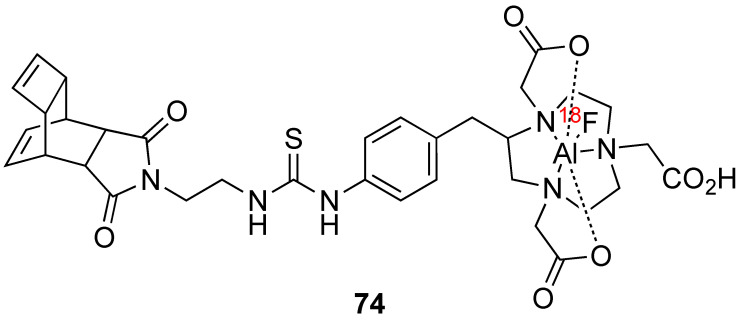
[^18^F]-FAl-NOTA Reppe anhydride adduct for in vivo coupling.

A non-canonical radiolabelling method was engaged for in vivo radiofluorination of cancer cells pre-treated with GdF_3_-HSA-tetrazine nanoparticles. In this case, addition of [^18^F]-FAl-NOTA complexes featuring a Reppe anhydride **74** (Figure 41) allowed the in vivo orthogonal bioconjugation [61].

Another in vivo application of the strained promoted reactions, in this case a alkyne–azide cycloaddition, has been reported to occur between a radiofluorinated azide and aza-dibenzocyclooctyne functionalizing the surface of mesoporous silica nanoparticles [144]. Indeed, after the latter were injected and passively accumulated in tumours, they were shown to react with radiofluorinated azido–PEG **44** based on both PET imaging and necropsy data. The fluorination is achieved classically but in this case **44** is injected (2 h after the nanoparticles) and reacts in vivo.

**Figure 42:**
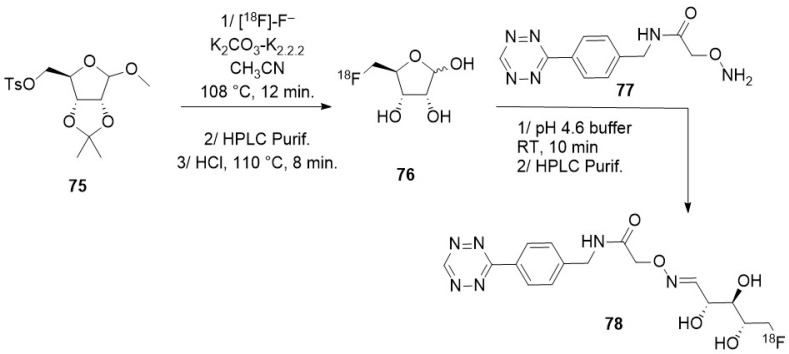
Synthesis of [^18^F]-labelled tetrazine **78** from [^18^F]-5-fluoro-5-desoxyribose **75**.

A related approach has been applied for the in vivo radiofluorination of porous silicon nanoparticles [138] pre-targeted with a *trans*-cyclooctene (TCO) moiety. In vivo, the nanoparticle-carried TCO reacted with **78** made of a [^18^F]-5-fluoro-5-deoxy-ribose equipped with a tetrazine terminus. The synthesis of **78** is described in Figure 42. In a first step, [^18^F]-5-fluoro-5-deoxy-ribose **76** is obtained by a classical tosylate displacement from **75** in the presence of [^18^F]-fluoride, K_2_CO_3_, and K_2.2.2_, followed by HPLC purification and acidic hydrolysis. In a second step, a tetrazine decorated with an aminoxy function **77** allowed a straightforward oxime linkage with **76** to form **78**. This oxime was then purified again by HPLC. Although the production of the tetrazine derivative appears somehow cumbersome, this approach represents an illustration of the pre-targeting concepts for in vivo labelling of nanoparticles.

To conclude on this overview of the methods involved for the radiolabelling of nanoparticles, as for most radiofluorinations, the smartest approach will consist in application of the “keep it simple” principle. Thus, late radiofluorinations, avoiding tedious purification of any [^18^F]-containing intermediates, shall be preferred. In this perspective, one can forecast an increasing use of the radiolabelling techniques based on main group elements such as alumina, silicon, and boron. The radiofluorination of metal-based nanoparticles featuring oxygen atoms might also take advantage of their high thermal stabilities. The generation of such nanoparticles incorporating [^18^O]-oxygen atoms can allow the formation of ^18^F through the nuclear ^18^O(p,n)^18^F by direct bombardment of the nanoparticles. Yet in this case the nature of the metal has to be taken into account to avoid the generation of long-lived isotopes through nuclear reaction of the metal itself.

## 4. Purification Methods and Characterization of Radiolabelled Nanoparticles

Most articles devoted to radiopharmaceuticals are generally built around three main components: the preparation/synthesis of the compound to be radiolabelled, the radiolabelling method (also including the purification stage), and its biological evaluation (in vitro and/or in vivo). While in the case of a molecular radiocompound, the purification and/or characterization stages of the radioproduct formed are generally provided and detailed (i.e., purification by various chromatography techniques, confirmation of the structure of the radioproduct by HPLC in comparison with its cold analogue, etc.), in the case of radiolabelled nano-objects, the information is often less precise and/or very heterogeneous from one study to another. In other words, in-depth characterization of radiolabelled nanoparticles is generally sorely lacking. This lack of information is also found in the majority of reviews devoted to nanoparticles for SPECT and PET imaging, where no specific item on the purification/characterization of the radioproduct is discussed [22,159,160,161,162]. However, there are several recent review articles highlighting the various methods for purifying and characterizing nanoparticles in the field of radiopharmaceuticals/radiolabelled compounds [163,164,165], confirming the growing need for purification/characterization processes for these radio nano-objects. Readers can refer to recent reviews for further details on the various purification procedures for radiopharmaceuticals and/or the techniques available for the physico-chemical characterization of radiolabelled nanoparticles (including their advantages, disadvantages, and limitations) [163,164].

While the radiolabelling and purification process must be as fast as possible and above all compatible with the short-life of the given radioisotope (110 min for fluorine-18, as mentioned in the introduction), it is clear that the intrinsic nature of the nano-object and the radiolabelling method chosen (direct or indirect) have a strong impact on this process. Due to these radioprobes having a high molecular weight, the purification method of choice is size-exclusion chromatography (SEC) or (ultra)centrifugation and the general use of TLC (or radio-TLC) is the simplest and most widely used technique used to assess the radiochemical purity (RCP) and radiochemical yield (RCY) of radiolabelled nanoparticles. However, alternative purification techniques could be used depending on the nature of the nanoparticles. Thus, considering that the nature of the nanoparticle has more impact on the purification/characterization techniques than the radiolabelling stage, we will categorize the purification stages of various radiofluorinated nano-objects by type of nanoparticles (organic or inorganic platforms as described in the first section). With the help of selected examples, the differences between each process and/or the information provided will be highlighted, and the characterization of either the radioproduct obtained or the cold analogue based on fluorine-19 will be discussed.

### 4.1. Organic Platforms

#### 4.1.1. Liposomes, Micelles, and Derivatives

Various [^18^F]-liposomal drug delivery systems were studied [27]. Depending on the radiolabelling approach chosen, i.e., surface or intraliposomal radiolabelling (see radiolabelling section), purification methods may vary.

Bioorthogonally labelled [^18^F]-liposomes were developed by mixing 3-[^18^F]fluoro-1,2-dipalmitoylglycerol ([^18^F]-FDPG), as the radioactive tracer, with a lipid formulation [34]. After sonication during 30 min, then filtration through a 0.22 μm filter, the [^18^F]-labelled liposomes were suspended in phosphate-buffered saline and obtained with a >95% RCP. More interestingly, the sizes and polydispersity indexes (PDI) of the [^18^F]-labelled liposomes and of their cold non-labelled surrogates, measured by DLS, were very close (d = 142.9 nm and PDI = 0.207 for ^18^F-compounds vs. d = 130.3 nm and PDI = 0.081 for [^19^F]-analogues). In a similar strategy published previously, the [^18^F]-FDPG-labelled liposome mixture was sonicated and then extruded through a series of filters (the authors indicated 11 passes through 200 μm diameter pores, then 11 passes through 100 μm diameter pores). It was then purified on gel chromatography (Sephadex G-75, ORTG, Inc. (Oakdale, TN, USA)) using PBS as eluent to remove non-incorporated [^18^F]-FDPG [30]. The size distribution of [^18^F]-radiolabelled liposomes, measured over one day using a particle size analyzer Nanotrac, was stable over time and comparable to the size of the non-radiolabelled liposomes (mean diameter: 29 ± 9 nm).

An alternative approach called “solid-phase transition method” was used for the one-step [^18^F]-labelling of synthesized and pre-formulated liposomes [29]. After incubation of fluorinated amphiphilic intermediates (previously synthesized, purified by semi-preparative HPLC column (C18-column) and obtained with a 100% RCP) with the liposomal solution, the mixture was purified by ultracentrifugation (20 min at 320,000× *g*). The efficiency of incorporation of radiofluorinated amphiphilic compounds into liposomes was then determined by HPLC. Interestingly, the authors observed no significant difference in stability between the [^18^F]-liposomes and the non-radioactive analogues, confirming the similar structuration of the nano-objects and the possibility of carrying out an initial screening with non-radiolabelled liposomes before developing radiofluorinated analogues of the most promising compounds.

Radiofluorinated liposomes can also be obtained by encapsulating radioactive drugs in the aqueous core of the liposome (intraliposomal radiolabelling). Incorporation of [^18^F]-FDG into different negatively, positively, or neutrally charged liposome formulations was achieved by repeated freeze–thaw cycles in liquid nitrogen [166]. The liposomes were extruded and then purified by centrifugation to remove untrapped [^18^F]-FDG. Three passes through a 100 nm pore-size polycarbonate filter were required before centrifugating the liposome saline solution (180,000× *g* for 20 min). A similar process to purify fluorine-labelled dasatinib nanoformulations (micelles and liposomes) was used [14]. Dasatinib was loaded into PEGylated water-soluble nano-carriers. The dasatinib was chemically modified prior to radiofluorination which was performed with no carrier–added [^18^F]-KF- Kryptofix^®^ 222complex. After HPLC purification, the pure radioactive intermediate was suspended in NaCl 0.9% and then filtered through a 45 μm filter. It was encapsulated in a liposome (or micelle) using a conventional method. The [^18^F]-liposomal formulation was obtained using a Mini Extruder (19 passes through a 100 nm pore-size polycarbonate membrane) and did not require any additional purification. Interestingly, a DLS study of the radiolabelled liposomes (and micelles) was carried out and the loading densities were evaluated. A mean particle size of 120 nm and a drug/lipid molar ratio of 1:2000 were determined for [^18^F]-labelled dasatinib liposomes.

Recently, lipid–PEG derivatives with a thiol function or including a peptide bearing a thiol function were incorporated to sphingomyelin nanometric emulsions (SNs) and then radiolabelled with 4-[^18^F]-fluorobenzamido-N-ethylmaleimide ([^18^F]-FBEM) [12]. Both radiolabelled nanometric emulsions (with or without peptide) were purified by PD-10 desalting column and exhibited hydrodynamic sizes, polydispersity indexes, and ζ potentials (around 140 nm, 0.2 and +38 mV, respectively) similar to those of the non-radioactive analogues, confirming that the radiolabelling approach had no dramatic impact on the nanosystems stability. It should be noted that an extensive HPLC study of the [^18^F]-radiolabelled lipid-based nano-carriers degraded in ethanol demonstrated a highly specific radiolabelling through a maleimide-thiol coupling by comparison with the non-radioactive analogue [^19^F]-compounds. Interestingly, no trace of the [^18^F]-FBEM precursor was observed in any of the chromatograms, thus validating the efficiency of the purification method.

#### 4.1.2. Polymers

Most of the [^18^F]-radiolabelled polymers were generally purified by (ultra)centrifugation or SEC setup. Radioactive [^18^F]-radiolabelled polyester-based nanoparticles were obtained by the nano coprecipitation method using [^18^F]-radiolabelled block-copolymers. Whatever the method used to prepare the radiofluorinated nanoparticles, the suspension was purified by centrifugation (4000× *g* during 45 min at room temperature) with ultracentrifugal filters [45]. The efficiency of the radiolabelling process (and by extension the purification process) was validated by comparing the amount of radioactivity after centrifugation in the precipitate and the supernatant. Surprisingly, the size measurement (diameter < 200 nm, PDI ranging from 0.05 to 0.28) and the ζ potential (around −30 mV) were determined on the radiofluorinated compounds after the complete decay of fluorine-18. The authors concluded that the incorporation of the radioisotope did not modify the physical properties of the nanoparticles.

Modified PLGA nanoparticles were radiolabelled on the basis of the strong non-covalent interaction between avidin and biotin. Specifically, avidin-modified PLGA nanoparticles were [^18^F]-labelled with the freshly synthesised biotinylated [^18^F]-prosthetic group (purified by semi-preparative HPLC) in a buffer solution. After the radiolabelling step, the efficiency of PLGA radiolabelling was monitored. The resulting radioactive suspension was sonicated, centrifuged (10,000× *g* for 10 min), and the amount of radioactivity was measured in the filtrate and the precipitate. The amount of radioactivity being at background level in the supernatant, the authors demonstrated that a 5 min sonication of the radiolabelled nanoparticles suspension was sufficient to obtain pure radiolabelled polymer nanoparticles that can be injected into animals [44].

Size-exclusion chromatography, to separate the smaller compounds from the labelled polymer, is an alternative to centrifugation for purifying radiofluorinated polymers. For example, after the [^18^F]-labelling of folate-pHPMA conjugates, the radioactive mixture was purified by SEC using three Hitrap desalting columns (Sephadex G-25 resin) in series with 0.9% NaCl solution as solvent [40]. Previously published examples of radiofluorinated HPMA-based polymers also used similar SEC purification. Pure solutions of [^18^F]-labelled polymers, ready for in vivo evaluation, were obtained after purification using Hitrap desalting column (Sephadex G-25 Superfine) with 0.9% NaCl solution and a flow rate of 0.5 mL/min [42,167] or with PBS buffer solution and a flow rate of 1 mL/min [41]. Very recently, the radiolabelling of an anionic amphiphilic teroligomer using a CuAAC strategy was studied [168]. Due to the radiolabelling strategy and the anionic nature of the polymer, size-exclusion chromatography (semi-preparative SEC) was performed after a first purification step by solid-phase extraction (to remove the excess of copper salts). The radioactive teroligomer mixture was successively loaded on an anionic exchange cartridge (Chromafix SB cartridge, size M, Macherey-Nagel GmbH & Co., KG, Düren, Germany) and eluted using aqueous 1M HCl solution as eluent, and then the resulting solution was neutralized before being purified using two Hitrap desalting columns in series with phosphate buffer as eluent. Although the polymer was obtained with excellent radiochemical purity and a total reaction time (around 2 h) compatible with the half-life of fluorine-18, the authors indicated a 30% loss of radioactivity during purification by SEC, which explains the rather low radiochemical yield (around 15%). It should be noted that no information was provided by the authors on any loss of radioactivity during the initial purification by solid phase extraction.

In the case of the first tumour-targeting nanoparticles reported with fluorine-18, the purification step was a little different [50]. Using selected amphiphilic block-copolymers, nanoparticles and micelles were characterized by DLS (in solution) prior fluorination (hydrodynamic diameters from 47 to 142 nm). After reaction with [^18^F]-fluoride in the presence of Kryptofix^®^ 222and K_2_CO_3_, the radiofluorinated nanoparticles were diluted with water and passed through a column containing Dowex strongly acidic ion exchange resin, followed by a short plug of alumina. These conditions removed all of the Kryptofix^®^ 222 and most of the unreacted fluoride ion. The radiochemical purity measured by radio-TLC was 61% (i.e., 31% of the fluoride was incorporated into the nanoparticles). No control of the size after labelling was performed.

#### 4.1.3. Dendrimers

A few examples of radiofluorinated dendrimers have been reported as functionalized PAMAM derivatives (half dendrimers) [53]. Aryltrifluoroborates functionalized dendron-biotin conjugates (4, 6, and 16 branch dendrons) were radiolabelled with [^18^F]-FF^−^ before the resulting solution was purified via a silica cartridge using an acetonitrile/water mixture as eluent. The purification step was monitored using TLC (ACN/water 7/3) and no trace of free or unreacted [^18^F]-fluoride was detected in the solution. PAMAM (G6) dendrimer derivatives [54], radiolabelled via a [^18^F]-prosthetic group, were purified using a Sephadex G-25 column. The radiochemical purity was then checked using analytical HPLC (C18-column, eluent: water 0.1% TFA/ACN). More recently, various works based on the radiosynthesis and preclinical evaluation of a G4 PAMAM hydroxyl dendrimer (called OP-801) as a [^18^F]-PET tracer were reported [55,56]. A click chemistry strategy (CuAAC bioconjugation) was used to radiofluorinate the dendrimer, named [^18^F]-OP-801. It was first purified by semi-preparative HPLC (Phenomenex Biosec-SEC-s-2000) and the resulting solution was then passed through a Sep-Pak C18 plus cartridge (washed with sterile water before elution using dehydrated ethanol (1 mL) and then 0.9% sodium chloride solution (10 mL)). The RCP was >95% and the purity of the radiocompound was checked by analytical HPLC (Phenomenex Jupiter 5 mm C18). More interestingly, the radiosynthesis and purification steps were optimized and automated using a purification procedure identical to that mentioned above (HPLC followed by trapping on a Sep-Pak C18). Clinical grade [^18^F]-OP-801 suitable for human use was thus produced under “Good Manufacturing Practices” conditions, and enhanced quality control was implemented to meet the criteria of the food and drug administrations. Thus, several batches of radioactive compounds were prepared, purified, analysed, and identified. A calibration curve (developed with the cold analogue [^19^F]-OP-801) was used to determine the total carrier mass of non-radioactive [^18^F]-OP-801 and derivatives. The structure of [^18^F]-OP-801 was confirmed by a comparative HPLC study (similar retention times for [^18^F]-OP-801 compared with [^19^F]-OP-801).

#### 4.1.4. Nanodroplets

Perfluorocabon (PFC) nanodroplets were radiolabelled by adding a radiofluorinated monomer ([^18^F]-CF_3_(CF_2_)_7_(CH_2_)_3_F) in the presence of an emulsifier (perfluorohexane with a fluorosurfactant (Zonyl-FSO) or perfluorooctylbromide with a lipid solution) [10]. The mixture was sonicated for 10 min, and then centrifuged for 20 min at 4000 rpm. The resulting precipitate was redispersed in distilled water to a volume concentration of 6% radioactive droplets. The [^18^F]-PFC droplets were stable (loss of [^18^F]-F^−^ below 2.4% after 2 h) and the determination of their classical properties (hydrodynamic diameters and ζ potentials) showed no change compared to their non-radioactive analogues.

### 4.2. Inorganic Platforms

Although the nanoparticle size is also the main criterion for the purification steps of radiofluorinated inorganic nanoparticles, depending on their nature (e.g., magnetic nanoparticles) and composition, other purification and/or characterization techniques shall be used, such as X-ray diffraction (XRD) or X-ray photoelectron spectroscopy (XPS) for the determination of structure/crystallinity or surface speciation, respectively.

#### 4.2.1. Rare Earth Nanoparticles

Numerous examples of radiofluorination of rare earth nanoparticles (REs) have been reported over the last decade [58,59,62,66,69,70,84]. As said above, this growing interest is based on the strong binding between [^18^F]-F^−^ and the rare earth cations as well as on the unique 4f electronic structure of rare earth ions, which gives them peculiar optical and/or magnetic properties, and could therefore lead to bi- or trimodal (optical and/or magnetic and nuclear) imaging nanoprobes.

Whatever the system studied (naked REs or coated REs or REs functionalized by a biological vector), the nanoparticles (and their eventual surface coating) were characterized before radiofluorination using the standard analytical techniques (TEM, XRD, XPS, IR, TGA, etc.). Then, radiolabelling was carried out by incubating the REs with [^18^F]-FF^−^ (from 1 to 10 min at room temperature) and the purification process consisted of a first centrifugation step followed by several water washes with sonication and centrifugation to remove the unreacted [^18^F]-F^−^. Radiopurity was monitored by radio-TLC. It is suggested that long-time and high-speed centrifugation of ultrasmall REs during the purification stage could induce aggregation of the nanoparticles. This hypothesis was put forward to explain the high uptake of radiolabelled sub-5 nm KGdF_4_ REs in the lungs (45% ID/g) [59].

**Figure 43:**
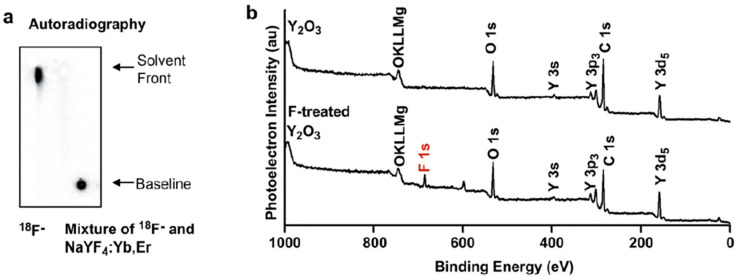
^18/19^F-REs characterization. (**a**) [^18^F]-labelling monitory of REs (autoradiography performed in saline buffer; (**b**) rare earth nanoparticles before and after incorporation of a fluorine source (tetra-n-butylammonium fluoride) in CHCl_3_ at ambient temperature and purification by washing and centrifugation (reprinted from Biomaterials, 32, Sun, Y.; Yu, M.; Liang, S.; Zhang, Y.; Li, C.; Mou, T.; Yang, W.; Zhang, X.; Li, B.; Huang, C.; Li, F. Fluorine-18-labeled rare earth nanoparticles for positron emission tomography (PET) imaging of sentinel lymph node, 2999–3007, Copyright 2011, with the permission from Elsevier).

Interestingly, in one case, a model study of the radiolabelling/purification process with cold nanoparticles ([^19^F]-material) was reported [58]. To establish the reaction between fluoride ions and RE nanoparticles, tetra-*n*-butylammonium fluoride (as a source of [^19^F]-F^−^) was incubated with F127-modified Y_2_O_3_ nanoparticles for 1 min at room temperature in CHCl_3_ before purification (centrifugation + washings), and the incorporation of FF^−^ was investigated by XPS. In contrast to the starting material, the F_1s_ peak at 685.6 eV was observed after ^19^F incorporation (Figure 43b, XPS spectra). Using the same protocol, [^18^F]-radiolabelled REs were easily obtained, as shown in the Figure 43a, autoradiography, with no trace of residual [^18^F]-F^−^ detected, thus validating the purification process.

#### 4.2.2. Metal Oxides

One example of ceria (CeO_2_) nanoparticles [^18^F]-radiolabelling was reported in 2012 [89]. The amino groups of the functionalized CeO_2_ nanoparticles were incubated with [^18^F]-SFB, 1 h at room temperature. The radioactive mixture was purified by successive centrifugation/washing cycles until no radioactivity was detected in the supernatant (4000 rpm for 10 min/PBS). Pure [^18^F]-CeO_2_ nanoparticles were suspended in PBS/tween 80 and sonicated 5 min prior to in vivo injection. Structural analysis (elemental analysis, thermogravimetry, and IR spectroscopy) of CeO_2_ nanoparticles was carried out before the radiolabelling to demonstrate their successful functionalization and the presence of 3-aminopropyl groups anchored on the ceria surface (the density of aminopropyl groups was approximately 4.4 mmol g^−1^).

Various works described [^18^F]-radiolabelling of metal oxides using the click chemistry strategy such as that on [^18^F]-nanoparticles based on cross-linked dextran iron oxide ([^18^F]-CLIO) [74]. In this work, the [^18^F]-propargyl PEG_3_ synthon was readily clicked onto azido–PEG-modified CLIO in PBS buffer. Microcentrifugation filtration of the resulting mixture yielded pure [^18^F]-CLIO. The radiochemical purity determined by analytical HPLC (C18 column) was >98%. More recently, RGD peptide and fluorine-18 were coupled onto ultrasmall iron oxide nanoparticles (USPIO) by click chemistry [78]. The resulting solution was purified by ultrafiltration using 100kDa molecular weight cut-off filters to remove the unreacted [^18^F]-prosthetic moiety ([^18^F]-FEA). Filtration was carried out until the amount of radioactivity was at background level in the filtrate. The nanoparticle solution was then concentrated before being used. The radiolabelling and purification process were completed in just 1 h. The first in vivo biodistribution of [^18^F]-RGD@USPIO revealed a high uptake in bones that was attributed by the authors to the incomplete elimination of unreacted [^18^F]-FEA during the purification process. However, whether this bone uptake could be linked to an in vivo defluorination reaction is questionable. It is noteworthy that, in this case, the isotopologue [^19^F]-RGD@USPIO was prepared in the same conditions and its physico-chemical features determined: core size of 6.5 ± 0.5 nm and average hydrodynamic diameter of 40.44 nm.

**Figure 44:**
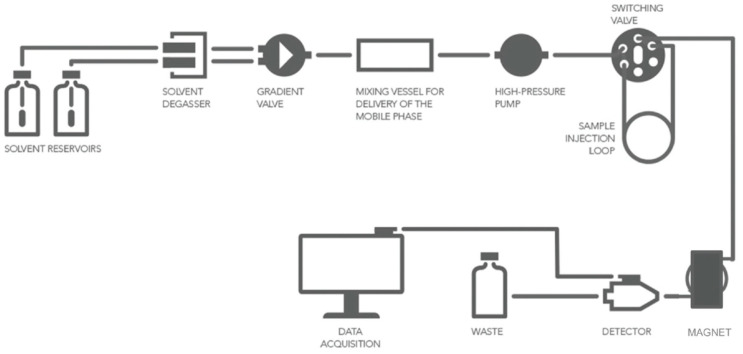
Magnetic separation strategy: schematic construction of the HPLC system including a magnet (Used with permission of Biomaterials Science, from ^18^F-labeled magnetic nanovectors for bimodal cellular imaging, Schütz, M.B.; Renner, A.M.; Ilyas, S.; Lê, K.; Guliyev, M.; Krapf, P.; Neumaier, B.; Mathur, S., 9, 2021, Copyright 2013, permission conveyed through Copyright Clearance Center, Inc.).

Unlike for molecular species, HPLC is rarely used to purify inorganic nanoparticles due to uncontrolled aggregation that could definitely clot the capillaries. However, modification of a HPLC system was recently reported as an innovative strategy for the purification of [^18^F]-radiolabelled Fe_3_O_4_ nanoparticles [79]. The HPLC system was modified by removing the column, and coiling the capillary tube, through which the solution circulates, around a magnet (Figure 44). Thus, during purification of the solution, the non-magnetic particles (unreacted materials, side-products, etc.) are rapidly eliminated during elution/washing of the solution, while the radiolabelled nanoparticles are retained by the magnet. Once the magnet is removed, the pure nanoparticles can be collected. Interestingly, this magnetic separation process could be extended to other radiolabelled magnetic nanoparticles.

This strategy was applied to purify radiolabelled aluminium hydroxide-coated magnetite nanoparticles with [^18^F]-F^−^. Indeed, after Fe_3_O_4_@Al(OH)_3_ and [^18^F]-F^−^ were incubated for 10–15 min at room temperature in water, the solution was centrifugated at 3000–4000 rpm for 20 min and the residue washed several times with water until no radioactivity was collected in the filtrate, before being re-suspended in saline solution. It was further shown that magnetic separation using a strong permanent magnet (NdFeB magnet) could replace the purification by centrifugation. It should be noted that the radiolabelling and purification steps were validated upstream with [^19^F]-nanoparticles [82,83].

An alternative route towards [^18^F]-labelled water-soluble functionalized iron oxide (IO) nanoparticles is to stabilize [^18^F]-aluminium fluoride ([^18^F]-AlF) using a chelator such as NOTA (1,4,7-triazacyclononane-1,4,7-triacetic acid) [80]. [^18^F]-AlF labelling and [^19^F]-AlF chelation of NOTA-IO nanoparticles were performed. In both cases, the desired product (([^18/19^F]-AlF)-NOTA-IO nanoparticles) was purified using a size-exclusion column (PD-10) with PBS as eluent then concentrated with a centrifugal filter (MWCO = 30 kDa). The number of AlF units per nanoparticle determined by ICP-MS, at different pH values, ranged from 1243 ± 294 at pH = 4 to 624 ± 137 at pH = 4.5, this last value (and consequently the associated radioactivity) being sufficient for the authors to carry out radiolabelling at pH 4.5.

Syntheses of [^18^F]-labelled metal oxide nanoparticles by direct proton beam irradiation of ^18^O-enriched aluminium oxide or ^18^O-enriched titanium oxide were also studied [85,90]. For example, after irradiating ^18^O-enriched aluminium oxide with 16 MeV protons, the radioactive nanoparticles were stored until the ^13^N (produced during the proton irradiation) had completely decayed (t_1/2_ = 9.97 min). The radioactive nanoparticles were suspended in NaCl solution, sonicated for 2 min then centrifuged at 8000× *g* before being used. This elegant radiolabelling method requires no real purification and has no dramatic impact on the main features of the nanoparticles, as XRD and DLS measurements taken before and after irradiation showed no relevant changes.

The last example of radiofluorination of metal oxide nanoparticles concerns zinc oxide nanoparticles [88]. [^18^F]-ZnO nanoparticles were obtained by CuAAC reaction. The radioactive nanoparticles were purified by centrifugation (10,000 rpm for 5 min) and the precipitate was washed with HEPES buffer three times prior being resuspended in HEPES buffer. The radiochemical purity determined by TLC was >95%. In vivo experiments confirmed the efficiency of the purification step, as no bone uptake was observed. So, no unreacted [^18^F]-F^−^ or [^18^F]-PEGazide **44** (see Figure 29) intermediate were present in the injected [^18^F]-ZnO nanoparticles solution.

#### 4.2.3. Gold Nanoparticles

While gold nanoparticles have been widely used in biological applications [169], the first biological evaluation of [^18^F]-labelled gold nanoparticles was published in 2012 [120]. They were obtained by conjugation reaction between [^18^F]-SFB **38** (see Figure 25) and the amino group of the lysine residues of the peptide (peptide CK) anchored to the nanoparticles. The crude solution was centrifugated at 4200 rpm (*ca.* 1980 g) for 15 min and the resulting precipitate was washed three times until no amount of radioactivity was detected in the supernatant, then the solid was suspended in sodium citrate/tween80 mixture. The nanoparticles were fully characterized by DLS, XPS and UV-Vis prior [^18^F]-labelling. Based on the radioactivity/mass ratio, an average of 27 [^18^F]-atoms per nanoparticle was determined. In addition, stability studies were carried out with [^19^F]-gold nanoparticles and with [^18^F]-labelled gold nanoparticles, according to UV-Vis spectra. [^18^F]-labelled nanoparticles were stable for at least 5 h in PBS solution.

**Figure 45:**
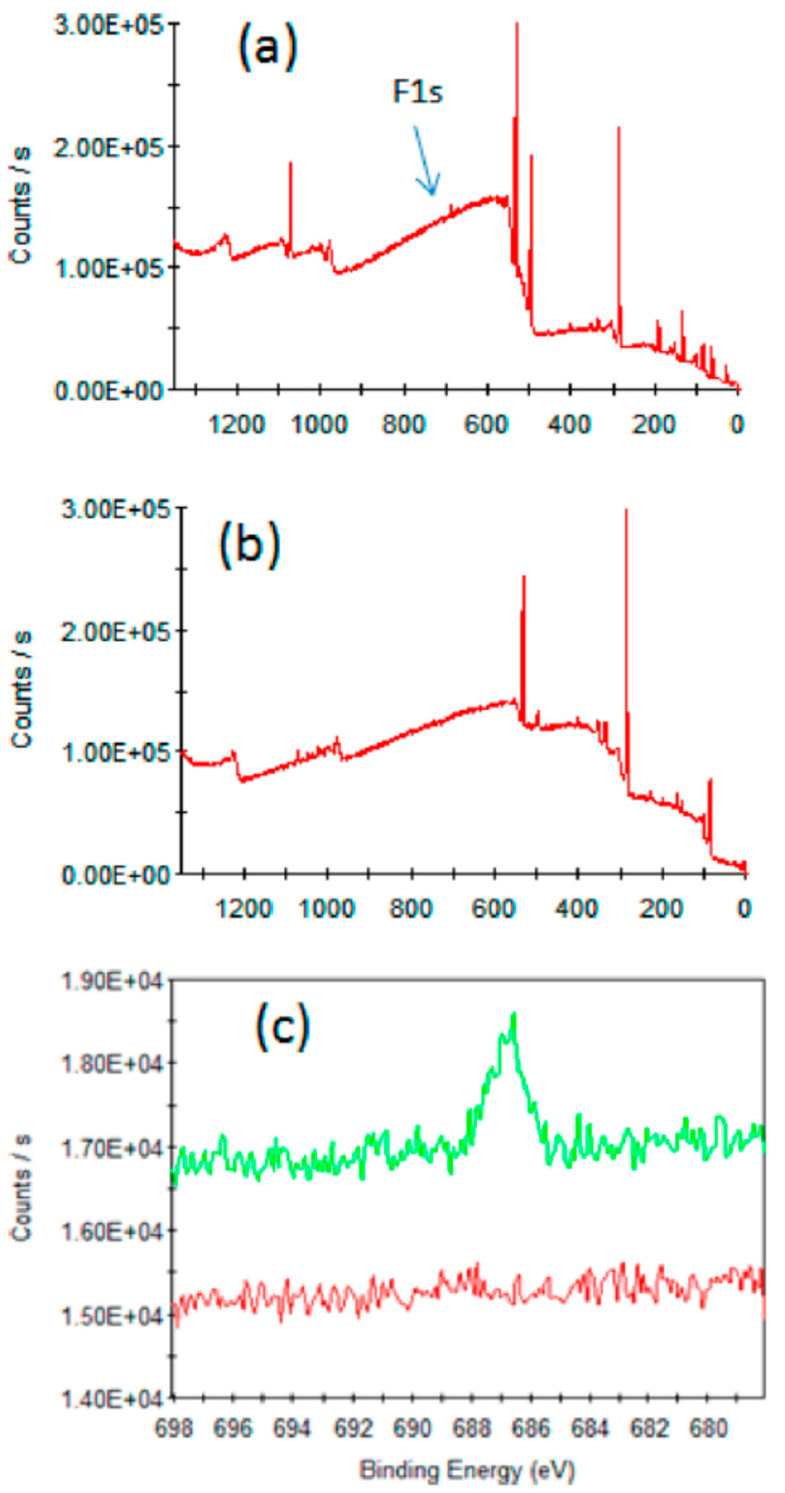
XPS spectra proving the [^18^F]-radiolabelling of gold nanoparticles. (**a**) [^18^F]-SiFA-maleimide-nanoparticles; (**b**) maleimide-nanoparticles; (**c**) offset high resolution scans of F1s of [^18^F]-SiFA-maleimide-nanoparticles (green) and maleimide-nanoparticles (red) (analysis carried out with partially decayed [^18^F]-SiFA-maleimide-nanoparticles) (Reprinted with permission from Rapid ^18^F-labeling and loading of PEGylated gold nanoparticles for in vivo applications, Zhu, J.; Chin, J.; Wängler, C.; Wängler, B.; Lennox, R.B.; Schirrmacher, R., Bioconjugate Chemistry 2014, 25, 1143–1150, Copyright 2014, American Chemical Society).

After rapid production of [^18^F]-PEGylated gold nanoparticles using SiFA radiolabelling strategy [122], size-exclusion gel purification (NAP-10 column with 0.1M sodium buffer as eluent) yielded pure [^18^F]-labelled gold nanoparticles. The purity was confirmed by radio-TLC (R_f_ = 0 for all [^18^F]-labelled nanoparticles). Based on stoichiometry, only three silicon-fluorine prosthetic groups [^18^F]-SiFA-SH (and therefore three fluorine-18) were grafted onto each maleimide-gold nanoparticle. The XPS data provided additional proof of successful Michael addition reaction by the appearance of a new peak related to the fluorine at 686.8 eV (corresponding to F_1s_) for the radiofluorinated gold nanoparticles (see Figure 45). As indicated by the authors, the radioactive nanoparticles were stored in a shielded container for 24 h until they had decayed sufficiently to be analysed by XPS. Given the decay of fluorine-18 over 24 h, one can consider that the fluorine peak observed by XPS is due to [^19^F]-nanoparticles and not to [^18^F]-nanoparticles.

Similar work in terms of purification and characterization was focused on the [^18^F]-radiolabelling of nitrone-gold nanoparticles using a bioorthogonal strain-promoted alkyne–nitrone cycloaddition (SPANC reaction) [124]. The radiofluorinated nanoparticles obtained from the SPANC reaction were passed through a PD-10 desalting column using 0.1M PBS buffer as eluent, leading to a pure [^18^F]-labelled gold nanoparticles solution ready to use. An extensive structural study (^1^H NMR, IR, TEM and thermogravimetric analysis (TGA)) was carried out on non-radioactive nanoparticles (nitrone-gold nanoparticles and [^19^F]-labelled gold nanoparticles). Briefly, the nitrone-gold nanoparticles were spherical with a gold core of 2.6 ± 0.5 nm wide and perfectly monodisperse. Remarkably, the authors determined the raw formula of the nanoparticles (Au_500_(MeO−EG_3_−S^−^)_200_(nitrone−EG_4_−S^−^)_40_, EG = ethylene glycol) as well as the amount of nitrone moieties per nanoparticle (0.840 μmol.mg^−1^). The feasibility of the SPANC reaction was mainly demonstrated by NMR and XPS analyses on the isotopologous ^19^F model. The ^19^F NMR spectrum of [^19^F]-labelled gold nanoparticle recorded in D_2_O showed only one peak at −222.91 ppm and comparison of the XPS spectra before and after conjugation exhibited the appearance of new peaks at 289.2 and 686.7 eV corresponding to C_1s_ and F_1s_ (C-F bond) for the [^19^F]-labelled gold nanoparticle, respectively. An average of 40 ^19^F atoms per nanoparticle were determined.

Finally, a less conventional nanoparticle architecture in which a [^18^F]-FDG derivative is conjugated to gold nanoparticles and anti-metadherin antibodies ([^18^F]-FDG-AuNP-Anti-MTDH) was described [170]. Radiolabelling was carried out in three steps but little information on the final purification of the nano-objects is given. [^18^F]-FDG-AuNP-Anti-MTDH were centrifuged, filtered (0.2 μm) before being dissolved in pure water. X-Ray analysis confirmed the presence of fluorine-18 on the [^18^F]-FDG-gold nanoparticles (FDG-AuNPs). The size and the ζ potential of the non-radioactive nanoparticles, [^19^F]-FDG-AuNP and [^19^F]-FDG-AuNP-Anti-MTDH, ranged from 15 to 40 nm and −13.30 to −18.06 mV, respectively, showing that antibody bioconjugation has no dramatic impact on the size and stability of the system.

#### 4.2.4. Pd Nanosheets

An universal one-pot synthetic approach for the radiofluorination of biomolecules (HSA or nanobodies) as well as palladium nanosheets using [^18^F]-fluorothiophosphate (FTP) synthons was very recently developed [125]. In the latter case, [^18^F]-FTP reacted with PEGylated Pd nanosheets (Pds-PEG) at room temperature for 15 min. The resulting mixture was purified by successive ultracentrifugations (13,000 rpm for 10 min, 3 times) until no radioactivity (due to unreacted [^18^F]-FTP and/or [^18^F]-F^−^) was detected in the supernatant. Radiochemical purity > 99% was monitored by radio-TLC analysis. No structural characterization of the [^18^F]-Pd nanosheets was given.

#### 4.2.5. Silicon and Carbon-Based Nanoparticles

Different works on [^18^F]-labelled mesoporous silica nanoparticles (MSNPs) were also reported. Using the same synthesis and purification methodologies as those used for CeO_2_ nanoparticles (vide supra), [^18^F]-MSNPs were prepared by grafting [^18^F]-SFB **38**) onto amino-functionalized MSNPs (30–130 nm) [143]. More recently, functionalized MSNPs (DBCO-PEG-MSNPs, DBCO = aza-dibenzocyclooctyne) were radiofluorinated using a pre-targeted strategy via a Cu free click conjugation (SPAAC reaction) [144]. The functionalized MSNPs were fully characterized. TEM showed that MSNPs were roughly spherical in shape, with an average diameter of 100–150 nm, and IR and ζ potential values confirmed the functionalization of the silica nanoparticles (ζ-potential value of −7.63 mV for DBCO-PEG-MSNPs vs. +32.63 mv for NH_2_-MSNPs). In this approach, as click conjugation (and therefore the radiolabelling of the nano-object) is carried out in vivo, the purification steps are limited to that of the radiofluorinated azide prosthetic group which was classically purified by reverse-phase HPLC.

[^18^F]-labelling of thermally hydrocarbonised porous silicon nanoparticles (THCPSi) is an interesting/unconventional example in that the nanoparticles are first radiolabelled before being coated with a fungal protein [137]. This two-step procedure required two (similar) purification processes. Thus, THCPSi were firstly incubated with [^18^F]-F^−^ for 10 min at 120 °C, followed by several cycles of centrifugation at 1500× *g*—wash in water/ethanol mixture using sonication, each lasting 20 min. Pure [^18^F]-THCPSi were collected in absolute ethanol, then coated with hydrophobin HFBII for 30 min at 80 °C. The final HFBII-[^18^F]-THCPSi particles were also purified by centrifugation followed by 3 washes in ultrapure water before being conditioned in sterile 0.9% NaCl. The coating obviously has an impact on the diameter of the [^18^F]-nanoparticles (average hydrodynamic size: 324 nm for HFBII-[^18^F]-THCPSi vs. 215 nm for [^18^F]-THCPSi), and on the ζ potential values, which remains increases slightly (from −33 mV to −21 mV). Still, the fungal protein coating of the HFBII-[^18^F]-THCPSi acted as a shield, resulting in a lower loss of [^18^F]-F^−^ ions in vivo (and therefore lower bone uptake) than for uncoated [^18^F]-THCPSi (0.04% for HFBII-[^18^F]-THCPSi vs. 0.21% for [^18^F]-THCPSi ID %/g at 60 min).

[^18^F]-nanodiamonds were studied by using radiolabelling and purification methods identical to those previously reported by the same group for ceria [89] or mesoporous silica nanoparticles [143] (purification by successive centrifugation/washing cycles until no radioactivity was detected in the supernatant) [130]. Remarkably, the authors proposed (in the supplementary data of [130]) a structural study of these nano-objects with fluorine-19. [^19^F]-nanodiamonds were prepared and purified in the same way as [^18^F]-nanodiamonds, then analysed in detail by AFM, TEM, IR and ^19^F NMR. Using an NMR internal standard [^19^F]-NaF, a percentage of 4.3% of fluorine-labelled groups in [^19^F]-labelled nanodiamond was assessed (NMR study carried out in deuterated methanol).

Radiofluorinated PEG-functionalized nano-graphene oxides ([^18^F]-nGO-PEG) were synthetized and studied as PET probes [131]. nGO-PEG were easily radiolabelled by being incubated with [^18^F]-F^−^ for 1 h at 80 °C in acidic conditions. Unreacted [^18^F]-F^−^ was classically removed by filtration using 0.2 μm filter and centrifugation/washes in saline solution cycles until no radioactivity was detect in the filtrate. The efficiency of the radiolabelling was monitored using radio-TLC (eluent: EtOAc/*n*-hexane; no migration for [^18^F]-nGO-PEG, R_f_ = 90 mm for [^18^F]-F^−^). Non-fluorinated nGOs were characterized by XRD profiles, ζ potential values and TEM images. More interestingly, the efficiency of radiolabelling was validated on [^19^F]-nGO-PEG by XPS (F_1s_ peak at approximately 725 eV) and also by IR. Indeed, the presence of fluorine is confirmed by (i) a sharp decrease in the intensity of the band at 3429 cm^−1^ due to the disappearance of hydroxyl groups (substituted by fluorine) and (ii) in the 1100–1150 cm^−1^ region, the disappearance of the C-O band and the appearance of the C-F band.

These last two examples perfectly illustrate the importance of conducting in-depth structural studies on non-radioactive nanoparticles ([^19^F]-labelled) in parallel with the radiofluorination investigation.

In conclusion, due to the relatively short lifetime of fluorine-18, the purification step, like the radiolabelling method, needs to be simple, efficient, and as fast as possible to obtain a [^18^F]-radiolabelled compound of high radiochemical purity. On the basis of the examples cited above, it seems unarguable that whatever the intrinsic nature of the nanoparticle, the main purification criterion taken into account is its size/molecular weight, making steric exclusion chromatography or (ultra)centrifugation the two gold-standard methods for purifying radiolabelled nano-objects. However, for magnetic nanoparticles, magnetic separation with a strong NdFeB magnet can be an advantageous alternative to steric exclusion chromatography or centrifugation (in which aggregation of nanoparticles can occur).

In addition, as mentioned in a recent and very comprehensive review on nanoparticles characterization techniques in radiopharmaceutical sciences [164], the following statement shall be strongly considered. “In general, there is a drastic lack of extensive characterization of nanoparticles used in radiopharmaceuticals sciences. In some works, these characterizations have not even been indicated”. While it is true that in many studies, the structural characterization of nanoparticles was more or less complete, or even minimalist, and very often focused on the nanoparticle before radiolabelling, more substantial studies (carried out on [^18^F]-labelled and/or cold [^19^F]-labelled nanoparticles) highlighted the (radio)fluorination (by XPS study in particular) and/or the number of fluorine atoms/ions coupled to the nanoparticle. On the other hand, the integrity of the nanoparticles structure is often little discussed/controlled, whereas the addition of fluorine-18 or a radiofluorinated prosthetic group can have a real impact on the structure of the nanoparticle itself, as does the radiofluorination method used. This criterion therefore remains to be better understood in the design of future radiofluorinated nano-objects. Finally, it seems essential to characterize all the new radiolabelled nanoparticles as precisely as possible because the better the structural characterization of the object is, (i) the easier it will be to understand the structure/in vivo uptake relationship, (ii) the more controlled the radiolabelling stage will be (minimum quantity of radioactivity to be introduced into the nanoparticle, rapid purification, etc.), and (iii) the faster the translation from the bench to the bedside can be achieved.

## 5. Discussion

As illustrated above, a large number of publications concern the radiofluorination of nanoplatforms, spanning all types of nanoparticles from organic nanoplatforms such as micelles, emulsions, liposomes, and polymers, to inorganic nanoparticles. The latter are either inert, such as silica, or display intrinsic physical properties such as some carbon or silicon-based nanoplatforms, gold, iron oxide and rare earth nanoparticles, or even quantum dots. Their sizes and shapes are diverse as well as their surface coatings, hence surface charges. Given the non-invasive character of radio imaging, the radiolabelling of these nanoplatforms was used for monitoring them in vivo and understanding their behaviour or predicting their therapeutic effectiveness. Nanoparticle radiofluorination was also envisaged to develop contrast agents for in vivo and in vitro imaging, as nanoparticles offer significant advantages over small molecules, such as the generation of a stronger and longer-lasting contrast. Different approaches have been developed aiming at efficient labelling and purification methods to reduce the overall time to the target, which is key in radio imaging.

As illustrated in the Section 3 of this manuscript, the main approaches to radiofluorinated NPs are:

- direct radiofluorination either by bombardment of [^18^O] containing nanoparticles, reaction of [^18^F]-fluoride with the inorganic core of the nanoparticles or incorporation of radiolabelled constituents during their synthesis (see Section 3.2) and,
- introduction of an organic based radiofluorinated compound by supramolecular interaction or covalent bonding, this last conjugation being achieved either classically in vitro or more recently using in vivo pre-targeting approaches (see Section 3.3).

Each approach obviously presents its pros and cons. For example, methods involving the direct generation of the nanoparticles with radiolabelled materials (as described in Section 3.2.3) are synthetically classical from the point of view of nanoparticles generation. Yet, due to the short half-life of [^18^F]-fluorine, they mainly exhibit moderate radiochemical yields (especially not decay corrected). Still, they do represent interesting approaches to generate new tools and evaluate their ability to perform the intended imaging. However, concerns may appear for routine preclinical, and even more for clinical, investigations. This is due not only to the production process leading to low radiochemical yields but also to the difficulty to perform extensive analyses to assess the quality of the radiofluorinated probes, due to obvious radioprotection issues.

From that perspective, radiofluorination of pre-formed and well-characterized nanoplatforms, can appear more promising. Indeed, their absence of toxicity can be assessed prior to radiolabelling (even though a different behaviour of radiolabelled nanoparticle formulations compared to their unmodified analogues cannot be ruled out).

An interesting case of nanoparticles [^18^F]-radiolabelling is illustrated by the direct bombardment of inorganic nanoparticles incorporating ^18^O atoms. This method has been described to proceed with a good efficiency. Its main issue remains the generation of a range of radioactive elements (other than ^18^F, such as ^13^N, ^44g^Sc, etc.) depending on the architecture of the nanoparticle. As the elements described are short lived positron emitters, the impact on dosimetry should be limited, but imaging might be affected by the different decays making the quantitative analysis less straightforward.

Late-stage introduction of a radiolabelled motif represents a common alternative. Different routes have been explored. Direct introduction of [^18^F]-fluoride appears especially valuable for inorganic based nanoparticles as it represents a short path towards radiolabelled nanoparticles (Section 3.2.2). It is of obvious interest in terms of production time, especially when the reaction can be carried out under aqueous conditions. Still the kinetics of the radiolabelling can be highly dependent on the nature of the nanoparticle and its coating. Moreover, the impact of the radiofluorination conditions on the physico-chemical characteristics of the nanoparticles, even if low molar amounts of fluoride are engaged, needs to be established in a repeated number of experiments (to obtain meaningful statistics).

An alternative method consists in the preparation of a radiofluorinated prosthetic group, that is further coupled to a properly surface engineered nanoparticle to display reactive functional groups (Section 3.3.2). This approach parallels the radiolabelling of biomacromolecules such as peptides or proteins. It is an obvious valuable strategy when a highly efficient synthesis of the radiofluorinated prosthetic group is available from the production site. It is also unavoidable when the common radiofluorination by C–F bond formation (namely significant heating in polar solvents and basic conditions) can be deleterious for the nanoparticles (as it is for biomacromolecules).

Lastly, another essential factor associated with [^18^F]-fluorine labelling for in vivo imaging is that the time constraint related to the half-life time of this isotope shall not be exclusively seen from the point of view of the preparation and purification of the radiolabelled material. It is also important to consider the time devoted to its analysis prior to in vivo injection, and most importantly for large species, the time of biodistribution to the desired target and clearance from the blood stream. These considerations seem to have restrained the development of nanoparticles in [^18^F]-PET imaging. Indeed, ex vivo prepared radiofluorinated nanoparticles described in Section 3.3.1 and Section 3.3.2 have an imaging efficiency that strongly depends on their time to target. Nevertheless, an upcoming development shall now arise from the latest trends of in vivo labelling relying on bio-orthogonal chemical ligation (see Section 3.3.2). These methods are appearing as highly appropriate approaches. Indeed, they consist in a first injection of a pre-targeted nanoparticle, allowing the biodistribution of a perfectly characterized material. This is then followed (after the time required for the nanoparticle to reach the biological target) by the injection of a small molecule carrying the radioactive fluorine and the functional group complementary to one carried by the pre-targeted nanoparticle. It is noteworthy that contrarily to nanoprobes, very efficient analysis and purification techniques are available for molecular probes. Thus, this approach combines the best of both worlds in terms of synthesis, characterization and biodistribution.

However, two main limitations remain. The bio-orthogonal conjugation needs to present an extremely fast kinetic profile, and the accumulation of the radiolabelled probes needs to be driven only by the pre-targeting functions, and not by non-specific interactions. Advantageously, these latter issues have been circumvented in the related topic of pre-targeted antibodies for PET imaging which will benefit to further developments for nanoparticles radiofluorination [171].

Similar to the radiolabelling step, the purification of radiofluorinated nanoparticles must be rapid and efficient, and the process must be transposable to automated radiosynthesis. Most [^18^F]-nanoparticles are purified using size-exclusion chromatography or (ultra)centrifugation. Although a few elegant alternatives have been identified (direct proton beam irradiation requiring no purification except waiting for the decay of radioactive by-products produced during the proton irradiation, or magnetic separation), they are unfortunately very nanoparticle-dependent and cannot be transposed to the vast majority of those.

The characterization of fluorine-18 radiolabelled nanoparticles is undoubtedly the step that needs to be optimised/worked on for a future clinical routine usage, in order to meet the requirements of international authorities such as the European Medicines Agency or the Food and Drug Administration. To optimize radioprotection, and because a large number of analytical techniques cannot be performed on radioactive materials, the optimization of this stage necessarily involves a detailed structural study of [^19^F]-analogues using a set of numerous analytical techniques, as well as structural comparisons between [^19^F]-labelled and non-labelled systems (supposing first that the nanoparticle to be labelled has been carefully characterized, and that its stability in biological media, such as cell culture or plasma, has been ascertained). Care should be taken to generate the [^19^F]-analogue in as identical conditions as possible to those of [^18^F]-radiolabelling. This raises technical issues to bridge the low concentrations used in radiolabelling processes and the low sensitivity of the characterization techniques needed to ascertain the physico-chemical integrity of the nanoparticles, especially inorganic ones. Ensuring that data are transposable is thus a huge challenge. The methodology used for the structural characterization of generation 4 PAMAM dendrimers radiolabelled with fluorine-18 [55,56] is certainly an example to follow. However, this example surely represents the ideal case, given that a dendrimer is a macromolecular system which is easier to characterize than other types of nanoparticles.

Once purity assessed, lack of stability is the next potential bottle-neck. Indeed, release of the radiolabel in vivo would alter the biodistribution of the radioactivity which would no longer reflect the sole fate of the radiolabelled nanoparticles. Nuclear imaging’s relevance to nanotherapeutic translation thus depends on developments in radiolabelling strategies to obtain a high radiolabelling stability. First evaluations in vitro could point out a potential lack of stability. It could arise from the release of [^18^F]-fluoride, prosthetic group, or of the nanoparticle coating used to graft this prosthetic group, or of the fluorinated tracer in case of labelling via encapsulation. Such studies are scarce. For example, the [^18^F]-fluoride loading stability of superparamagnetic iron oxide nanoparticles, coated with polyacrylic acid and aluminium hydroxide, was tested in vitro in longitudinal experiments up to 4 h using water, buffer, and cell culture media at 37 °C [82]. After efficient synthesis of novel [^18^F]-labelled imaging agents based on YF_3_ nanoparticles, [^18^F]-labelled YF_3_ nanoparticles stability was studied in mouse and human serum [62]. In addition to study stability in buffer or storage medium, studies in biological medium as cell culture media, or best in plasma, are necessary because of the large number of interfering molecules in blood. These studies must be performed at 37 °C and over a period in accordance with future in vivo studies to be predictive of what will happen in vivo. This type of studies is particularly necessary for labelling strategies that rely on non-covalent binding of ^18^F.

Importantly in vitro studies need to be supplemented by cellular and in vivo studies to evaluate metabolism (e.g., hepatic) that could lead to radionuclide release. It could thus be interesting to perform blood and post-mortem tissue analysis to evaluate radiolabelling stability. On the reverse, as free fluoride is efficiently taken up in bones, quantitative in vivo PET imaging allows an indirect evaluation of the radiolabelling stability (but only as far as [^18^F]-fluoride release is concerned). As well the integrity of the nanoparticle needs to be ascertained. Some studies indeed report the detection of degradation products [16,89].

Then, even if the doses injected are very low (some mCi representing few pmol), the toxicity of the radiolabelled nanoplatform should be assessed. It is generally admitted that radiolabelling is unlikely to alter toxicity, and that it is rather the toxicity of the nanoparticle that must be evaluated. This can be done easily on the non-labelled nanoparticles (in case of labelling strategies that involve pre-formed nanoplatforms). Cell survival after exposure to different concentrations of nanoparticles and cell proliferation at different days post-exposition to nanoparticles could be studied as done by González-Gómez et al. to study superparamagnetic iron oxide nanoparticles [82]. However, one should keep in mind that the radiolabelling step may change the physico-chemical characteristics of the nanoplatform such as hydrodynamic size, surface charge, hydrophilicity balance…all parameters that may alter their interaction with cells and change their metabolism. Study of the toxicity of the [^19^F]-analogue, prepared under identical conditions, would be the best approach.

## 6. Conclusions and Future Directions

Our study of the literature shows a renewed interest for the radiofluorination of nanoplatforms in the past decade, flowering out of a continuously evolving radiofluorination ground (especially in the field of user-friendly radiofluorination of small molecules leveraging the labelling of nanoplatforms). These technologies are now mature to translate to highly sophisticated health-related uses. It appears that based on these evolutions, radiofluorinated nanoplatforms are especially suited when their local injection is envisaged, as reported in a recent work [172]. The interest in radiofluorination of nanoplatforms is also prompted by the development of pre-targeting approaches. This two-step technology consists first in the administration of the nanoplatform. Once the nanoplatform has disseminated in the body, and accumulated in the region of interest, a molecular radiotracer is administrated. The elegance of the process lies in the fact that both the nanoplatform and the molecular radiotracer are equipped with functional groups that will selectively react with each other, leading to their specific recognition in vitro and in vivo. Radioactivity thus accumulates in the region of interest, which can be imaged with high accuracy.

Provided that optimal in vivo bioconjugation conditions are found (proportion of the two components, time interval between the two injections, stable bioconjugation, neglectable non-specific interactions, etc.), the advantages should be numerous: (i) a range of effective and rapid bioorthogonal conjugation methods is available, (ii) characterization is simplified to purification of the radiofluorinated prosthetic group (using well-mastered chromatographic techniques) and that of the modified nanoparticle, and finally (iii) the technology can be applied to a large number of nanoparticles and is compatible with the short-lived radionuclides and relatively slow pharmacokinetics of nanoparticles (maximum tumour uptake after a few hours/days).

The implementation of this technology has emerged owing to the tremendous progress made in the surface engineering of nanoparticles, and taking advantage of the methodologies developed for macromolecular systems [173,174]. If properly performed, it should allow access to a wide range of diagnostic (bi- and multimodal imaging) or theranostic combinations, especially in the case of intrinsically active nanoparticles leading to improved monitoring of treatments. Based on imaging results, the treatment strategy can be adapted, with a direct impact on patient management, by protecting patients from unnecessary side effects and costs.

Thus, radio imaging of nanoparticles can play an important role in the management of the individual patient and novel radiofluorination methods can help to achieve this goal.

## Figures and Tables

**Table 1:** Selected examples of [^18^F]-labelled liposomes and purposes of the studies (NR: data not reported).

Constituents	Approximate Hydrodynamic Diameters (nm)	ζ Potential (mV)	Radiolabelling Location (Bilayer, Core, or Surface)	Objective of the Study	Ref.
PC: cholesterol	120	NR	Core	Evaluation of [^18^F]-dasatinib uptake in glioblastoma as a function of formulation to optimize dosage	[14]
DOPC: cholesterol plus surface coating (PEG or hyperbranched PG)	408, 92, and 168 for liposomes non-modified, modified with PEG, and modified with hyperbranched PG, respectively	NR	Bilayer with cholestene surface with PEGylated and hyperbranched polyethers	Comparison of the effect of hyperbranched PG surface coating to the one of linear PEG on the biodistribution and pharmacokinetics of liposomes	[18,23]
DOPC: cholesterol modified with hyperbranched PEG_38_ and trimannose	109	NR	Surface	PET assessment of trimannose as a liposome vector to target dendritic cells	[28]
DSPC: cholesterol	90, 170, and 570 depending on formulation	NR	Bilayer	Study of labelling method efficiency and liposomal trafficking	[29]
DPPC: cholesterol: DSPE-PEG_2000_	45	NR	Bilayer	Study of liposome trafficking	[30]
Hydrogenated soybean phosphatidylcholine: cholesterol: stearic acid, surface modified by PEG_5000_-DSPE	200–250	NR	Bilayer	Study of the delivery of oxygen into ischematic brain by liposome-encapsulated hemoglobine	[31]
DOTPA: cholesterol	218	+36	Core	Study of the effect of liposome encapsulation on the biodistribution of siRNA	[32]
DSPC: cholesterol, surface modified by DSPE- PEG_2000_	100	−2.9 ± 1.4	Bilayer	Study of liposomal drug delivery systems and if they could enter the ischemic brain region and be a therapeutic strategy for ischemic stroke treatment	[33]
DSPC: cholesterol, surface modified by DSPE- PEG_2000_, DSPE-PEG-TCO, and pHLIP	143	NR	Bilayer	Study of targeting by pHLIP and if it improved accumulation into tumours	[34]
DPPC: DSPE-PEG_2000_ targeted by arginine-rich peptides	From 70 to 200, depending on peptide loading and formulation	From −48 to +42, depending on peptide loading and formulation	Bilayer	Study of the effect of the presence and structure of targeting peptides on the adhesion of the formulation on blood vessel walls in the heart	[35]
DSPC: cholesterol, surface modified by PEG and targeted by a peptide (APRPG)	100	NR	Bilayer	Radiotracer delivery to image brain cancer tumours	[36]
DSPC: cholesterol surface modified by DSPE-PEG_2000_	NR	NR	Bilayer	Validation of an automated preparation method of radiolabelled liposomes, and study of liposome trafficking (tumour accumulation)	[37]
Cholestero l: sphingomyelin: mal-PEG-PE functionalized by phosphatidic acid or a lipidic derivate of TEG-curcumin	133 or 237 for liposomes with phosphatidic acid or lipidic derivate of TEG-curcumin, respectively	NR	Bilayer with radiofluorinated lipids Core with radiolabelled curcumin analogs	Evaluation of ability of functionalized liposomes to cross the blood–brain barrier and bind to the amyloid β plaque	[38]
DPPC: cholesterol: DSPE-PEG2000 surface modified by DPPE-DTPA-[^111^In]	168	−2.3	Core	Development of a dual [^18^F] (PET)/^111^In (SPECT) radio imaging tool	[39]

DOPC = 1,2-dioleoyl-sn-glycero-3-phosphatidylcholine; DPPC = 1,2-dipalmitoyl-sn-glycero-3-phosphocholine; DPPE-DTPA = 1,2-dipalmitoyl-sn-glycero-3-phosphoethanolamine-N diethylenetriaminepentaacetic acid; DOTPA = 1,2-dioleoyl-3-trimethylammonium-propane; DSPC = distearoylphosphatidylcholine; mal-PEG-PE = 1,2-distearoyl-sn-glycero-3-phosphoethanolamine-N-[maleimide(polyethylene glycol)-2000] ammonium salt;; PEG = polyethyleneglycol; DSPE-PEG = N-[monomethoxy-polyethyleneglycol-carbamyl] distearoylphosphatidyl-ethanolamine; PG = polyglycerol; pHLIP = pH (Low) Insertion Peptide; SiRNA = small interfering RNA; TCO = trans-cyclooctene; and TEG = triethyleneglycol.

**Table 2:** Main characteristics of the alumina nanoparticles studied (NR: data not reported).

Geometrical Size (nm)	Hydrodynamic Size (nm)	ζ Potential (mV)	Reference
5.5	9	NR	[90]
15	29.2	+3.3	[91]
35	35.1	+20.9	[91]
187	266	+9.3	[91]
	2528	−5.7	[91]

**Table 3:** Main characteristics of the hydroxyapatite nanoparticles studied in [92] (NR: data not reported).

Post-Synthetic Treatment	Geometrical Size (nm)	Shape	Hydrodynamic Size (nm)	ζ Potential (mV)
Calcination	41.6 ± 1.8	Spherical nanocrystals/aggregated	2784 ± 158	−15.8 ± 0.2
Hydrothermal	107.5 ± 6.2 × 25.8 ± 0.9	Rod-like	829 ± 135	−6.0 ± 0.6
None	132.0 ± 30.3 × 12.6 ± 2.2	Needle-like	2046 ± 470 (main peak)	NR
Alendronate	Supposed to be identical to the above		169 ± 33 (main peak)	NR
MeO-PEG-COOH			214 ± 68	NR

**Table 4:** Main characteristics of the [^18^F]-radiolabelled nanomaterials (NR: data not reported).

Material	Surface Coating/Stabilizer	Geometrical Size	Specific Surface Area (m^2^/g)	Pore Size (nm)	Pore Volume (cm^3^/g)	Hydrodynamic Size (nm)	ζ Potential (mV)	Ref.
TOPSi	None	1–10 μm	311	13	1.04	NR		[135]
TCPSi	None	1–10 μm	262	16.8	1.10	NR		[135]
THCPSi	None	1–10 μm	260	16.3	1.10	NR		[135]
THCPSi	Fluorescein Isothiocyanate/polyethylene glycol-15-hydroxystearate	142 nm	202	9	0.51	NR	−30	[136]
THCPSi	HFBII		323	7.4	0.48	234	−21	[137]
HCPSi	Glyceryl monostearate + L-α-phosphatidyl choline	190 (±40) nm	NR	NR	NR	193 (±30)	−40	[17]
THCPSi	PEG_12_ chains with trans-cyclooctene end groups	NR	NR	NR	NR	165	−40	[138]

## Nomenclature

Radiochemical Purity (RCP): the proportion of the total radioactivity in the sample that is present as the desired radiolabelled species (definition from the International Atomic Energy Agency).

Molar radioactivity: the ratio of the amount of radiofluorinated, [^18^F]-containing, to the amount of fluorinated, ^19^F and ^18^F containing, compound.

Specific radioactivity: the activity per unit mass of a radionuclide is a physical property of the specified radionuclide and is given by the equation: a[Bq/g] = $\frac{N_{A}ln2}{Mt_{1/2}..}$, where N_A_ is the Avogadro constant, M is the molar mass of the nuclide, and $t_{1/2}$ the half-life time of the radionuclide. For ^18^F, a = 1.71 × 10^9^ Ci.mol^−1^, thus the mCi to Ci range commonly used represents pmol to nmol quantities of fluoride.

Radiochemical yield: the amount of radiolabelled compounds divided by the initial amount of radioactive fluorine.

## Abbreviations

**Abbreviation**	**Meaning**
ACN	Acetonitrile
AMBF_3_	Alkylammoniomethyl-trifluoroborate
APM	N-(3-amino-propyl) methacrylamide hydrochloride
AT1	Angiotensin II receptor type 1
BFCA	Bifunctional chelating agent
BHT	Butylated hydroxytoluene
BSA	Bovine serum albumin
c.m.c	Critical micellar concentration
CaP	Calcium phosphonate
CDI	Carbonyldiimidazole
CLIO	Cross-Linked Iron Oxide
CT	X-ray computed tomography
CuAAC	Copper catalyzed alkyne–azide cycloaddtion
DBCO	Dibenzocyclooctyne
DCNP	Down-conversion nanoparticle
DLS	Dynamic light scattering
DMEM	Dulbecco’s Modified Eagle Medium
DMSO	Dimethyl Sulfoxyde
DOPC	1,2-dioleoyl-sn-glycero-3-phosphatidylcholine
DOPC	dioleoyl-*sn*-glycero-3-phosphatidylcholine
DOTPA	1,2-dioleoyl-3-trimethylammonium-propane
DPPC	1,2-dipalmitoyl-sn-glycero-3-phosphocholine
DPPE-DTPA	1,2-dipalmitoyl-sn-glycero-3-phosphoethanolamine-N diethylenetriaminepentaacetic acid
DSPC	Distearoylphosphatidylcholine
DSPE	Distearoylphosphatidylethanolamine
EPR effect	Enhanced Permeability and Retention effect
FBBA	Fluorobenzyl-2-bromoacetamide
FBEM	4-fluorobenzamido-N-ethylmaleimide
FDG	Fluorodéoxyglucose
FDP	Fluorodipalmitin
FDPG	Fluoro-1,2-dipalmitoylglycerol
FEA	2-fluoroethyl azide
FMT	Fluorescence molecular tomography
FPyME	(2-fluoropyrid-3-yloxy)propylmaleimide
FSB	N-succinimidyl 4-fluorobenzoate
FT-IR	Fourier-transform infrared spectroscopy
FTP	Fluorothiophosphate
GO	Graphene oxide
GRPR	Gastrin-releasing peptide receptor
GSH	Glutathione
HA	Hydroxyapatite
hbPG	Hyperbranched polyglycerol
HEPES	N-2-hydroxyethylpiperazine-N′-2-ethanesulfonic acid
HFBII	Class II hydrophobin
HPLC	High-performance liquid chromatography
HPMA	N-(2-hydroxypropyl)-methacrylamide
HSA	Human serum albumin
i.v.	Intravenous
ICP-MS	Inductively coupled plasma mass spectrometry
ID	Injected dose
IONP	Iron oxide nanoparticle
K_2.2.2_	Kryptofix^®^ 222
LAH	Lithium aluminium hydride
LEH	Liposome-encapsulating hemoglobin
LMA	Lauryl methacrylate
mal-PEG-PE	1,2-distearoyl-sn-glycero-3-phosphoethanolamine-N-[maleimide(polyethylene glycol)-2000] ammonium salt
MNP	Magnetic nanoparticle
MPC	2-methacryloyloxyethyl phosphorylcholine
MRI	Magnetic resonance imaging
MSC	Mesenchymal stem cell
MSNP	Mesoporous silica nanoparticle
MTDH	Metadherin
MWCO	Molecular weight cut-off
NFP	4-nitrophenyl 2-[^18^F]fluoropropionate
NFP	Fluoropropionate *p*-nitrophenol
nGO	Nano-graphene oxide
NIR	Near-infrared
NIRF	Near-infrared fluorescence
NMR	Nuclear magnetic resonance
NOTA	2-[4,7-Bis(carboxymethyl)-1,4,7-triazonan-1-yl]acetic acid
NP	Nanoparticle
NPB4	Fluorobenzylamide-poly(ethylene glycol)_4_-biotin
PAMAM	Poly(amido amine)
PBS	Phosphate-buffered saline
PDI	Polydispersity indexes
PDT	Photodynamic therapy
PEG	Polyethylene glycol
PEG-DSPE	N-[monomethoxy-polyethyleneglycol-carbamyl] distearoylphosphatidyl-ethanolamine
PET	Positron emission tomography
PFC	Perfluorocabon
PFCD	Perfluorocarbon droplets
PG	Polyglycerol
pHLIP	pH (Low) Insertion Peptide
pHPMA	Poly-N-(2-hydroxypropyl) methacrylamide
PLA	Polylactic acid
PLGA	Poly(lactic-co-glycolic acid)
PLL	Polylysine
pLMA	Poly(lauryl methacrylate)
POX	Polyoxazolines
PS	Polysarcosine
QD	Quantum dot
QMA	Quaternary MethylAmine resin
RCP	Radiochemical purity
RCY	Radiochemical yield
RE	Rare earth nanoparticle
ROMP	Ring-opening metathesis polymerization
ROP	Ring-opening polymerization
rTLC	Radio thin-layer chromatography
SEC	Size-exclusion chromatography
SFB	N-succinimidyl 4-[^18^F]fluorobenzoate
SiFA	Silicon-fluoride-acceptor
SiRNA	Small interfering RNA
SLNC	Solid lipid nanocomposite
SN	Sphingomyelin nanometric emulsions
S_N_2	Nucleophilic substitution second order
S_N_Ar	Nucleophilic aromatic substitution
SPAAC	Strain-promoted alkyne–azide cycloaddition
SPANC	Strain promoted alkyne–nitrone cycloaddition
SPECT	Single-photon emission computed tomography
SUV	Standardized uptake value
TATE	Octreotate
TCO	Trans-cyclooctene
TCPSi	Thermally carbonized porous silicon
TEG	Triethyleneglycol
TEM	Transmission electron microscopy
TFA	Trifluoroacetic acid
TGA	Thermogravimetric analysis
THCPSi	Thermally hydrocarbonized porous silicon
TLC	Thin-layer chromatography
TOPSi	Thermally oxidized porous silicon
UC	Up-conversion
UCNP	Up-conversion nanoparticle
USPIO	Ultrasmall superparamagnetic iron oxide
UV-Vis	Ultraviolet–visible spectroscopy
XPS	X-ray photoelectron spectroscopy
XRD	X-ray diffraction
